# Mapping the viruses belonging to the order *Bunyavirales* in China

**DOI:** 10.1186/s40249-022-00993-x

**Published:** 2022-07-07

**Authors:** Ai-Ying Teng, Tian-Le Che, An-Ran Zhang, Yuan-Yuan Zhang, Qiang Xu, Tao Wang, Yan-Qun Sun, Bao-Gui Jiang, Chen-Long Lv, Jin-Jin Chen, Li-Ping Wang, Simon I. Hay, Wei Liu, Li-Qun Fang

**Affiliations:** 1 State Key Laboratory of Pathogen and Biosecurity, Beijing Institute of Microbiology and Epidemiology, 20 Dong-Da Street, Fengtai, Beijing, 100071 People’s Republic of China; 2 Department of Research, Qilu Hospital, Cheeloo College of Medicine, Shandong University, Jinan, 250012 People’s Republic of China; 3 Division of Infectious Disease, Key Laboratory of Surveillance and Early-Warning on Infectious Disease, Chinese Center for Disease Control and Prevention, Beijing, 102206 People’s Republic of China; 4 Department of Health Metrics Sciences, School of Medicine, University of Washington, Seattle, WA USA; 5 Institute for Health Metrics and Evaluation, University of Washington, Seattle, WA 98121 USA

**Keywords:** *Bunyavirales*, Crimean-Congo hemorrhagic fever virus, Rift Valley fever virus, Ecological niche model, Risk assessment

## Abstract

**Background:**

Viral pathogens belonging to the order *Bunyavirales* pose a continuous background threat to global health, but the fact remains that they are usually neglected and their distribution is still ambiguously known. We aim to map the geographical distribution of *Bunyavirales* viruses and assess the environmental suitability and transmission risk of major *Bunyavirales* viruses in China.

**Methods:**

We assembled data on all *Bunyavirales* viruses detected in humans, animals and vectors from multiple sources, to update distribution maps of them across China. In addition, we predicted environmental suitability at the 10 km × 10 km pixel level by applying boosted regression tree models for two important *Bunyavirales* viruses, including Crimean-Congo hemorrhagic fever virus (CCHFV) and Rift Valley fever virus (RVFV). Based on model-projected risks and air travel volume, the imported risk of RVFV was also estimated from its endemic areas to the cities in China.

**Results:**

Here we mapped all 89 species of *Bunyavirales* viruses in China from January 1951 to June 2021. Nineteen viruses were shown to infect humans, including ten species first reported as human infections. A total of 447,848 cases infected with *Bunyavirales* viruses were reported, and hantaviruses, *Dabie bandavirus* and Crimean-Congo hemorrhagic fever virus (CCHFV) had the severest disease burden. Model-predicted maps showed that Xinjiang and southwestern Yunnan had the highest environmental suitability for CCHFV occurrence, mainly related to *Hyalomma asiaticum* presence, while southern China had the highest environmental suitability for Rift Valley fever virus (RVFV) transmission all year round, mainly driven by livestock density, mean precipitation in the previous month. We further identified three cities including Guangzhou, Beijing and Shanghai, with the highest imported risk of RVFV potentially from Egypt, South Africa, Saudi Arabia and Kenya.

**Conclusions:**

A variety of *Bunyavirales* viruses are widely distributed in China, and the two major neglected *Bunyavirales* viruses including CCHFV and RVFV, both have the potential for outbreaks in local areas of China. Our study can help to promote the understanding of risk distribution and disease burden of *Bunyavirales* viruses in China, and the risk maps of CCHFV and RVFV occurrence are crucial to the targeted surveillance and control, especially in seasons and locations at high risk.

**Graphical abstract:**

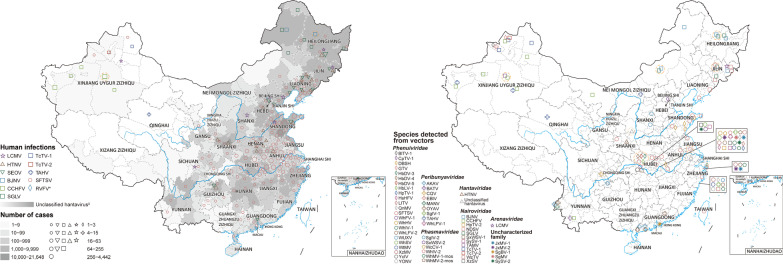

**Supplementary Information:**

The online version contains supplementary material available at 10.1186/s40249-022-00993-x.

## Background

The *Bunyavirales* is a large RNA virus order and comprises more than 560 viruses divided into twelve families[1], among which most members could infect humans and animals, thus posing a great potential threat to public health[2]. For example, hantaviruses usually cause two clinical syndromes when infecting humans, including hemorrhagic fever with renal syndrome (HFRS) and hantavirus cardiopulmonary syndrome (HCPS). HFRS cases were mainly reported in Eurasia, with case fatality rates (CFR) from ranging less than 1.0 to 15.0% [3], while HCPS cases were mainly reported in the Americas, with CFRs from 21.4 to 35.0% [4–6]. With recently increasing number of emerging and re-emerging *Bunyavirales*-related infectious diseases [7], viruses belonging to the order *Bunyavirales* have attracted much attention.

In China, related investigations were focused on the spatial distribution and ecological niches of HFRS [8–10], along with severe fever associated with thrombocytopenia syndrome (SFTS) [11–13]. Focus on these two severest *Bunyavirales*-related infectious diseases, resulted in little attention being paid to other diseases caused by *Bunyavirales* viruses. Crimean-Congo hemorrhagic fever (CCHF), also known as Xinjiang hemorrhagic fever (XHF), was first reported in 1965 in the Xinjiang Uygur Autonomous Region in China. The outbreak killed 10 of 11 infected patients after a short incubation period, which showed the acute onset and high fatality rate characteristic of CCHF [14]. However, two studies drew different conclusions for high-risk areas of CCHF occurrence, one in Xinjiang and the southwestern border of Yunnan, and the other in the central region of China [15–17]. In addition, the first imported case of Rift Valley fever (RVF), another infectious disease related to *Bunyavirale* mainly distributed in Africa and expanding to the Arabian Peninsula, was identified in Beijing in 2016 [18, 19]. With frequent international travel and global climate change, China possesses advantageous conditions for Rift Valley fever virus (RVFV) transmission, denoting the necessity for risk assessment of RVFV invasion and indigenous transmission [20]. Due to increasing variability and expansion of *Bunyavirales* viruses, a systematic understanding of nationwide distribution, epidemiological characteristics, and ecological niches of *Bunyavirales* viruses is still lacking. Such an understanding is needed for developing diagnostic and treatment guidelines and control programs.

Here we mapped the spatial distribution of *Bunyavirales* viruses detected from humans, vectors and animals at the city level in China. In addition, we assessed the environmental suitability of Crimean-Congo hemorrhagic fever virus (CCHFV) and RVFV at the 10 km × 10 km pixel level by applying boosted regression tree (BRT) models. Based on the model-projected risks and volume of air travel, we also estimated the imported risk of RVFV from its endemic areas to the cities in China. Our study will help to guide future surveillance and control of the *Bunyavirales* viruses from an ecological perspective.

## Methods

### Data on *Bunyavirales* viruses

**Fig. 1:**
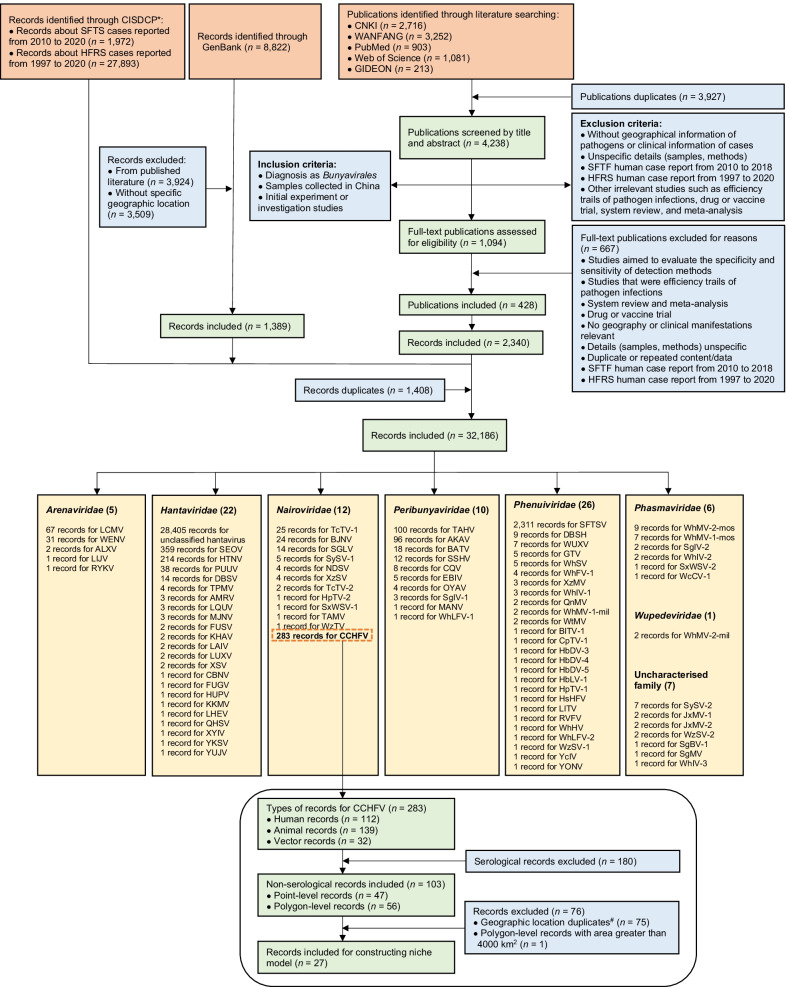
The flow diagram of data collection for *Bunyavirales* viruses in China. *China Information System for Disease Control and Prevention (CISDCP); the report data of SFTS and HFRS from CISDCP including laboratory-confirmed and clinically diagnosed cases, were both annual data at the county level. A record represents the number of SFTS or HFRS cases reported in a year at the county level. ^#^Geographic location duplicates included between point-level records and point-level records, between polygon-level records and polygon-level records, between polygon-level records and point-level records (the record about polygon corresponding to the point-level record was removed). The full name of each pathogen was shown in Table 1

We established a database of *Bunyavirales* virus occurrence in China from a variety of sources, including (1) the report data of human SFTS cases (2010–2020) and HFRS cases (1997–2020) from China Information System for Disease Control and Prevention (CISDCP), (2) literature reporting the occurrence of *Bunyavirales* viruses, published between January 1951 and June 2021, (3) and unpublished *Bunyavirales* virus data from the GenBank database (Fig. 1). For the report data from CISDCP, a record showed the number of SFTS or HFRS cases reported in one year at the county level. For the literature review, we collected information pertaining to *Bunyavirales* viruses detected in humans, vectors, and animals in China, based on PubMed (https://pubmed.ncbi.nlm.nih.gov/), Web of Science (http://www.webofscience.com/), Global Infectious Diseases and Epidemiology Network (GIDEON) (https://www.gideononline.com/) and two Chinese databases [Wanfang Data (http://www.wanfangdata.com.cn/) and China National Knowledge Infrastructure (CNKI, http://www.cnki.net/)] using data collected up to June 30, 2021 (Additional file 1: Table S1). Search terms used were predefined for viruses, including the order *Bunyavirales* and the families, genus, and species of *Bunyavirales* viruses and related diseases (Additional file 1: Table S2). Additional studies were identified by manually searching for references from the primary studies and previous reviews.

Studies were eligible if they described laboratory detections of *Bunyavirales* viruses in humans, vectors, and animals resulting from natural infections rather than laboratory challenges. However, we excluded studies that (i) lacked geographical information of viruses or clinical information of cases, (ii) had unspecified details (samples, methods), or (iii) other irrelevant studies including efficiency trails of virus infections, drug or vaccine trials, system reviews, or meta-analysis (Fig. 1). A record was defined as one laboratory-confirmed or clinically diagnosed human infection by any *Bunyavirales* virus species or viruses detected from vectors or animals, occurring at a specific time and location (defined by the administrative district or point with exact coordinates). Two authors (AYT and YYZ) conducted the initial searches independently and screened the retrieved studies following the inclusion and exclusion criteria as defined above. All conflicts of opinion and uncertainties were discussed and resolved by consensus with a third author (TLC). Some literature only identified the viruses in a broad scope, such as using “hantavirus” in lieu of reporting the specific genospecies, and thus we referred to these viruses as unclassified (e.g., unclassified hantavirus). The detailed procedures of viral data extraction and management were shown in the Additional file 1 (Fig. 1, Additional file 1: Table S3).

In addition, we supplemented another database of RVFV occurrence in RVF endemic areas in Africa and the Arabian Peninsula [19] from the Global Animal Disease Information System (EMPRES-i) database and literature, and the details were shown in the Additional file 1: Fig. S1, Table S1).

### Data on socioenvironmental, ecoclimatic and biological covariates

Forty-two explanatory covariates were chosen, mainly including meteorological data, volume of water in soil, surface runoff, minimum distance to the nearest bodies of water, elevation, land cover, Normalized Difference Vegetation Index (NDVI), mammalian richness, livestock density, and population density on global range. These covariates were used due to their potential effect on the environmental suitability for CCHFV and RVFV transmission based on the previous studies [15, 17, 21–28]. Predicted probability range of *Hyalomma asiaticum* presence in China was also included, because *H. asiaticum* was a major vector of the CCHFV in China (Additional file 1: Fig. S2) [29, 30]. Data sources of all the explanatory covariates involved in this study were introduced in detail in Additional file 1: Methods and Table S4). To assess the imported risk of RVFV, monthly data of international flights and passenger flow from RVF endemic countries to China in 2016 was collected from the International Air Transport Association (IATA, http://www.iata.org/).

### Spatial mapping

We summarized and mapped the distribution of *Bunyavirales* viruses at the city level if data were available or at the province level otherwise. Data included the overall number of human confirmed cases, the overall positive rate of humans with serological evidence, and the distribution of vectors and both livestock and wild animals. We also summarized information regarding the first identification of *Bunyavirales* virus infections in China and drew the matrix diagram of these *Bunyavirales* viruses and their infected vectors and animal hosts. It should be mentioned that PCR detection technology was not popularized in China until the twentieth century and the detection results of viruses during this period were mostly obtained by serological detection. As such, there is the possibility of false positive results resulting from immune cross-reactions. We have flagged the records obtained by serological testing on the results of this study.

### Modelling the CCHFV environmental suitability in China

The BRT models were used to examine environmental suitability of CCHFV occurrence in China. The algorithm of logistic structure was used to fit the presence/absence of CCHFV at the 10 km × 10 km pixel level in the BRT models. We divided all CCHFV records in humans, animals or vectors into point data with exact coordinates or polygon data with non-exact coordinates. The former was located by a 10 km × 10 km pixel for points, and the latter by administrative districts, such as counties, cities or provinces. The point data was directly connected with the explanatory covariates at the 10 km × 10 km pixel level, and the polygon data was connected with mean value of covariates at all 10 km × 10 km pixels in this polygon. Polygon records larger than 4,000 km^2^ were excluded in the modeling analysis due to the potential bias caused by uneven spatial scale. The locations of human cases and infections with CCHFV detected in animals and vectors by molecular or isolation assay records were included as presence data, but serological records in humans and animals were excluded due to the unguaranteed specificity of serological testing. The pseudo-absence data was generated by random sampling, the number was set to 15 times the presence data, and a weight of 1/5 was assigned.

To reduce potential overfitting and improve predictive accuracy of the models, we first evaluated pairwise Pearson correlations to reduce multicollinearity among the 36 variables including 19 ecoclimatic, 10 environmental, and 7 biological covariates (Additional file 1: Fig. S3, Table S5). For each group of variables with an absolute Pearson correlation coefficient higher than 0.7, we calculated the mean absolute value of correlation coefficients with all other variables (excluding this group of variables) for each variable in the group respectively, and the variable with the lowest mean was included for modeling. We then fitted an initial model and the variables whose relative contributions were in the top 10 were retained for the formal model-building. Finally, 10 screened variables were chosen, including 4 biological, 3 ecoclimatic and 3 environmental covariates (Table 1). The details of the BRT models were provided in Additional file 1: Methods.

**Table 1:** Mean (standard deviation) relative contributions of major explanatory covariates to the spatial distribution of CCHFV and RVFV, estimated by BRT models

Variable	CCHFV	RVFV
*Environmental*		
Shrubland	2.61 (1.77)	–
Agriculture	1.92 (1.16)	–
Grassland	1.33 (1.10)	14.51 (1.54)
Forest	–	6.12 (0.49)
Sparse vegetation	–	3.58 (0.20)
Elevation	–	9.31 (0.76)
Minimum distance to nearest bodies of water	–	5.54 (0.19)
Normalized difference vegetation index	–	4.81 (0.38)
*Ecoclimatic*		
Annual mean temperature	7.92 (2.48)	–
Precipitation seasonality	6.24 (2.43)	–
Temperature seasonality	4.00 (1.72)	–
Mean precipitation in previous month	–	21.17 (0.45)
Mean temperature in previous month	–	8.06 (0.64)
*Biological*		
Population density	2.28 (1.61)	5.31 (0.34)
Livestock density^a^	–	21.58 (1.13)
Goat density	3.23 (1.56)	–
Sheep density	1.89 (1.26)	–
Presence possibility of *H. asiaticum*	68.58 (4.22)	–

^a^Livestock density was the sum of the densities of buffalo, cattle, goat and sheep

“–” covariates were not included in the final model

### Modelling the seasonal dynamics of environmental suitability of RVFV in China

We established the native presence/absence niche model using occurrence records and explanatory covariates extracted from RVFV endemic areas (Africa and the Arabian Peninsula), and made projections using explanatory covariates from China to examine the environmental suitability in comparison. The occurrence of RVF was closely tied to meteorological conditions, such as the impact of El Niño on the RVF outbreak, and the impact of mosquitoes as important vectors of RVF. Thus, we established the BRT models on a month-year scale from 2000 to 2020, and included the monthly mean in temperature, precipitation, volume of water in soil, and surface runoff as covariates in the model (Additional file 1: Methods). In addition, for covariates usually calculated annually such as elevation, land cover, livestock and population density, the annualized indicators were included as the proxy for each month in the modeling analysis.

In total, we aggregated 18 variables over 20 years and performed multicollinearity screening (Additional file 1: Table S5, Fig. S4). Finally, 10 variables were chosen for the formal modeling analysis, including 6 environmental, 2 ecoclimatic, and 2 biological covariates (Table 1). Except for serological records, all locations with RVFV occurrence records in humans, animals, or vectors from the database of RVFV occurrence in Africa and the Arabian Peninsula were considered as presence data, and their annual and monthly information was extracted. For records with unspecific dates, we randomly assigned months from the available time range if the time span was less than 4 months, and excluded them otherwise. The pseudo-absence data was generated by random sampling with the number set to 3 times the presence data with randomly selected points on both the time scale and the spatial scale (the time and spatial range were the same as the occurrence records). Finally, we used the monthly environmental covariates of China from 2000 to 2020 to create 252 month-year prediction maps and averaged these maps by month to obtain the final monthly prediction maps. The details of the BRT models were provided in Additional file 1: Methods.

### Risk assessment

We converted the probability maps of CCHFV and RVFV occurrence into binary (at risk or non-risk) maps and calculated the area and population at risk for both viruses. The thresholds of CCHFV and RVFV models were determined by the cut-off values at which the Youden index of the test set was the maximum. We also calculated and mapped the number of months with environmental suitability for RVFV per year at the 10 km × 10 km pixel level. Considering that the suitable temperature for mosquitoes' survival from eclosion to adult emergence is at least 15 ℃, we adjusted the risk maps to prevent overestimation by converting all pixels with monthly temperatures of the previous month below 15 ℃ to no-risk [31]. In addition, the imported risk index per city in China was measured based on the model-projected risks and volume of air travel from RVFV endemic areas (Additional file 1: Methods). We only included the RVFV infected countries with evidence by molecular or isolation assay records, excluding measurements taken from serological records.

All analysis was performed in R 3.6.3 (R Foundation for Statistical Computing, Vienna, Austria) and ArcGIS 10.7 (ESRI Inc., Redlands, CA, USA).

## Results

### Distribution of *Bunyavirales* viruses in China

The literature search identified a total of 8165 publications, 428 of which were included in this study after duplicate removal, screen of title, and abstract and full-text review. These yielded 2340 records of *Bunyavirales* virus occurrence. An additional 1972 records of SFTS with 16,339 cases and 27,893 records of HFRS with 431,010 cases were collected from CISDCP, and 1389 unpublished records of viral sequences with geographic information were extracted from GenBank. In sum, 32,186 records were identified for this analysis (Fig. 1).

**Fig. 2:**
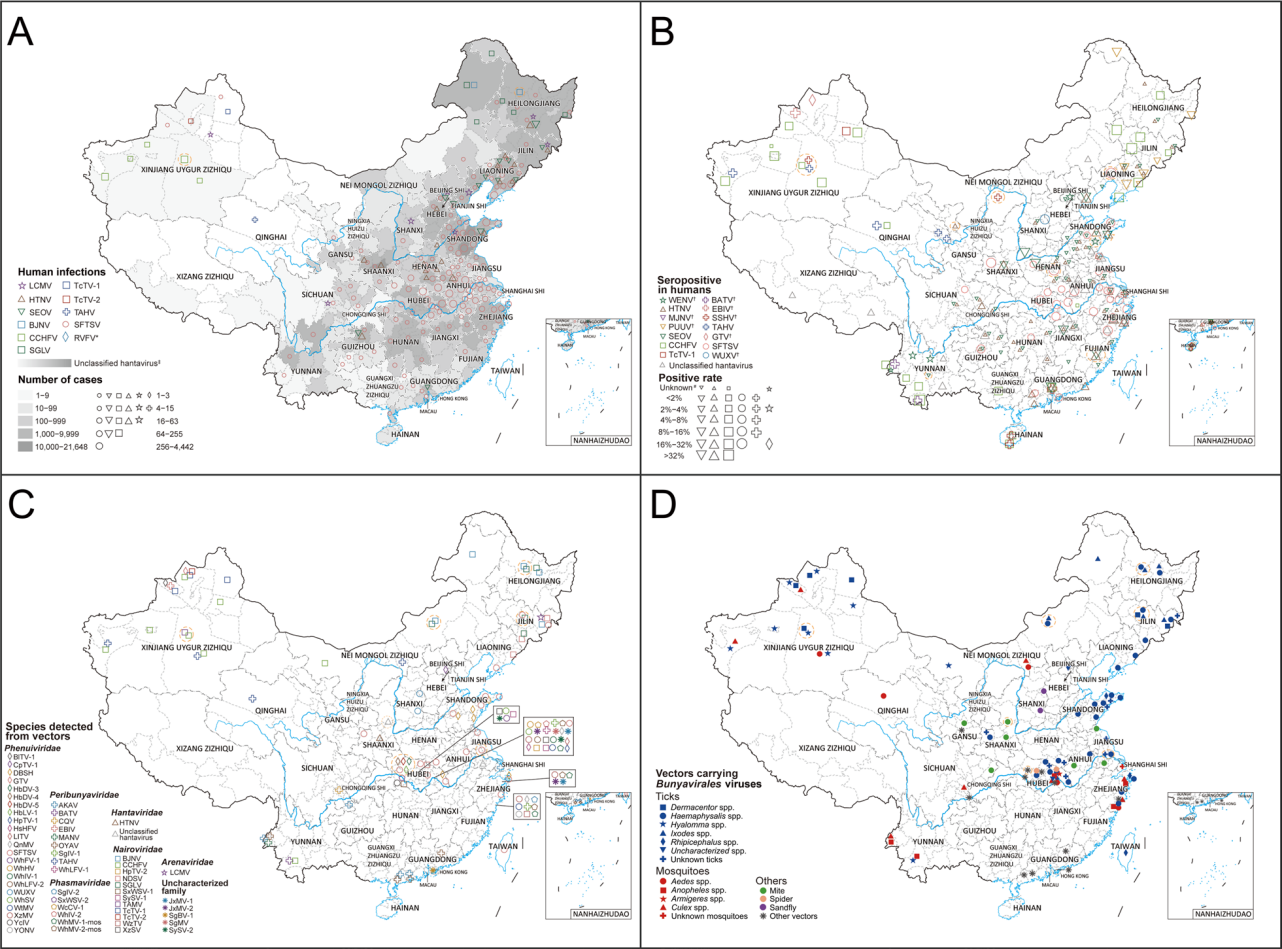
Geographical distribution of *Bunyavirales* viruses detected in humans and vectors. **A** Distribution of human cases infected with *Bunyavirales* viruses in China. *The RVFV case was imported. The number of human cases of unclassified hantavirus and SFTSV was calculated from the data of China Information System for Disease Control and Prevention. ^‡^Unclassified hantavirus was a general designation for the *Orthohantavirus* members of the *Hantaviridae* family including HTNV and SEOV in China, shown as polygon data, and the numbers represents different shades of gray. **B** The distribution of seroprevalence (infection rate of specific antibody/antigen) for *Bunyavirales* viruses in humans. ^†^Human beings infected with EBIV, GTV, SSHV, BATV, PUUV, WENV and WUXV were only reported with serological evidences. ^#^Positive rate was not calculated for the data with detections less than 10 or not provided. **C** The distribution of *Bunyavirales* virus species determined in vectors. **D** The distribution of vector species that carrying *Bunyavirales* viruses. *Bunyavirales* viruses in vectors were all detected by molecular methods or pathogen isolation. Locations with positive records were positioned at the center of either provinces or cities depending on data availability, among which the data at the province level were circled with dashed lines. The full name of each pathogen was shown in Table 1

Eighty-nine viral species belonging to the order *Bunyavirales* were reported in China from January 1951 to June 2021, consisting of 7 families, including 26 species of *Phenuiviridae*, 22 species of *Hantaviridae*, 12 species of *Nairoviridae*, 10 species of *Peribunyaviridae*, 6 species of *Phasmaviridae*, 5 species of *Arenaviridae* and one species of *Wupedeviridae*, as well as 7 viruses of uncharacterized family (Fig. 1). The number of newly-identified viruses of the order *Bunyavirales* (67/89, 75.3%) increased dramatically since 2010, and Hubei (36 species), Zhejiang (17 species), Yunnan (10 species) and Xinjiang (8 species) were the 4 provincial level administrative divisions (PLADs) with the largest number of newly-identified viruses reported (Table 2, Additional file 1: Fig. S5). The virus richness was consistent with the number of first identified viruses, and the highest richness of viruses occurred in Hubei province of central China (42 species), followed by Zhejiang province in southeastern China (25 species), Yunnan province in southwestern China (17 species) and Xinjiang Autonomous Region in northwestern China (14 species) (Fig. 2, Additional file 1: Fig. S6). *Phenuiviridae* was the predominant family in Hubei province, *Hantaviridae* in Zhejiang and Yunnan provinces, and *Nairoviridae* in Xinjiang Autonomous Region.

**Table 2:** The first identification site of 89 viruses belonging to the order *Bunyavirales* in China from January 1951 to June 2021

Pathogen name (abbreviation)	First identified origin (diagnosis methods^a^)	First identified prefecture (year)	References
*Arenaviridae*			
Lymphocytic choriomeningitis virus (LCMV)	Patients (A)	Beijing (1951)	[69]
Ryukyu virus (RYKV)	*Mus caroli* (B)	Yunnan (2013)	–
Wenzhou virus (WENV)	*Niviventer niviventer*; *Rattus flavipectus*; *Rattus losea*; *Rattus norvegicus*; *Rattus rattus*; *Suncus murinus* (B)	Wenzhou, Zhejiang (2013)	[70]
Alxa virus (ALXV)	*Dipus sagitta* (B)	Alxa League, Inner Mongolia (2014)	[71]
Lijiang virus (LIJV)	*Apodemus chevrieri* (B)	Lijiang, Yunnan (2015)	–
*Hantaviridae*			
Hantaan virus (HTNV)	*Apodemus agrarius* (A)	Xi'an, Shaanxi (1980)	[72]
Seoul virus (SEOV)	*Rattus norvegicus* (C)	Luoyang, Henan; Yuncheng, Shanxi (1981)	[73]
Xinyi virus (XYIV) ^b^	*Anourosorex yamashinai* (B)	Nantou, Taiwan (1989)	[74]
Dabieshan virus (DBSV)	*Niviventer confucianus* (A, B and C)	Anqing, Anhui (1992^c^)	[75]
Fusong virus (FUSV)	*Microtus fortis* (A and B)	Baishan, Jilin (2002^c^)	[76]
Yuanjiang virus (YUJV)	*Microtus fortis* (B)	Yiyang, Hunan (2002^c^)	[77]
Puumala virus (PUUV)	*Myodes rufocanus* (A)	Fushun, Liaoning; Baishan, Jilin (2003^d^)	[78]
Khabarovsk virus (KHAV)	*Microtus maximowiczii* (A and B)	Hulunbuir, Inner Mongolia (2004^c^)	[76]
Amur virus (AMRV)	*Apodemus peninsulae* (B)	Yanbian, Jilin (2005^c^)	[79]
Qian Hu Shan virus (QHSV)^b^	*Sorex cylindricauda* (B)	Diqing, Yunan (2005)	[80]
Cao Bang virus (CBNV)	*Anourosorex squamipes* (B)	Zunyi, Guizhou (2006)	[74]
Luxi virus (LUXV)	*Eothenomys miletus* (B and C)	Honghe Hani, Yunnan (2006)	[81]
Yakeshi virus (YKSV)	*Sorex isodon* (B)	Hulunbuir, Inner Mongolia (2006)	[82]
Thottapalayam virus (TPMV)	*Suncus murinus* (B)	Wenzhou, Zhejiang (2009)	[83]
Lianghe virus (LHEV)	*Anourosorex squamipes* (B)	Dehong, Yunnan (2010^c^)	[82]
Longquan virus (LQUV)	*Rhinolophus monoceros*; *Rhinolophus affinis*; *Rhinolophus sinicus* (B)	Lishui, Zhejiang (2011)	[82]
Imjin virus (MJNV)	*Crocidura lasiura* (B)	Ningbo, Zhejiang (2011^c^)	[84]
Fugong virus (FUGV)	*Eothenomys eleusis* (B and C)	Nujiang, Yunnan (2012)	[85]
Huangpi virus (HUPV)	*Pipistrellus abramus* (B)	Wuhan, Hubei (2012)	[82]
Kenkeme virus (KKMV)	*Sorex roboratus* (B)	Jiamusi, Heilongjiang (2012)	[86]
Laibin virus (LAIV)	*Taphozous melanopogon* (B)	Laibin, Guangxi (2012)	[87]
Xuan son virus (XSV)^b^	*Hipposideros pomona* (B)	Laibin, Guangxi; Puer, Yunnan (2012^c^)	[88]
*Nairoviridae*			
Crimean-Congo hemorrhagic fever virus (CCHFV)	Patients (D)	Kashi, Xinjiang (1965)	[39]
Nairobi sheep disease virus (NDSV)	*Haemaphysalis Iongicornis* (B)	Tonghua and Yanbian, Jilin; Dandong, Liaoning (2013)	[89]
Huangpi tick virus 2 (HpTV-2)	*Haemaphysalis* spp. (B)	Wuhan, Hubei (2015^d^)	[90]
Tacheng tick virus 1 (TcTV-1)	*Dermacentor marginatus* (B)	Tacheng, Xinjiang (2015^d^)	[90]
Tacheng tick virus 2 (TcTV-2)	*Dermacentor marginatus* (B)	Tacheng, Xinjiang (2015^d^)	[90]
Sanxia water strider virus 1 (SxWSV-1)	Unidentified *Gerridae* (B)	Yichang, Hubei (2015^d^)	[90]
Shayang spider virus 1 (SySV-1)	*Neoscona nautica*; *Parasteatoda tepidariorum*; *Plexippus setipes* (B)	Wuhan, Hubei; Jingmen city, Hubei (2015^d^)	[90]
Wenzhou tick virus (WzTV)	*Haemaphysalis hystricis* (B)	Wenzhou, Zhejiang (2015^d^)	[90]
Xinzhou spider virus (XzSV)^b^	*Neoscona nautica*; *Parasteatoda tepidariorum* (B)	Wuhan, Hubei; Jingmen city, Hubei (2015^d^)	[90]
Beiji nairovirus (BJNV)^b^	Patients (A and B)	Hulunbuir, Inner Mongolia (2017)	[52]
Songling virus (SGLV)^b^	Patients (A and B)	Songling, Heilongjiang (2017)	[53]
Tamdy virus (TAMV)	*Hyalomma asiaticum* (A)	Xinjiang (2018)	[91]
*Peribunyaviridae*			
Snowshoe hare virus (SSHV)	Healthy residents (C)	Shanghai (1982)	[92]
Akabane virus (AKAV)	Mosquitoes (A)	Shanghai (1998)	[93]
Batai virus (BATV)	*Anopheles philippinensis* (A)	Puer, Yunnan (1998)	[94]
Cat Que virus (CQV)	*Culex tritaeniorhynchus* (A)	Neijiang, Sichuan (2006^c^)	[95]
Tahyna virus (TAHV)	*Culex pipiens pallens* (A)	Kashi, Xinjiang (2006)	[96]
Manzanilla virus (MANV)	*Culex tritaeniorhynchus* (A)	Dehong, Yunnan (2010)	[97]
Oya virus (OYAV)	*Culex quinquefasciatus* (A)	Dehong, Yunnan (2010)	[98]
Ebinur Lake Virus (EBIV)^b^	*Culex modestus* (B)	Bo'ertala, Xinjiang (2012)	[99]
Shuangao insect virus 1 (SgIV-1)	Unidentified *Chrysopidae*; *Psychoda alternata* (B)	Wuhan, Hubei; Wenzhou, Zhejiang (2015^d^)	[90]
Wuhan louse fly virus 1 (WhLFV-1)^b^	Unidentified *Hippoboscoidea* (B)	Wuhan, Hubei (2015^d^)	[90]
*Phasmaviridae*			
Shuangao Insect Virus 2 (SgIV-2)^b^	*Abraxas tenuisuffusa*; *unidentified Diptera* (B)	Wenzhou, Zhejiang (2015^d^)	[90]
Sanxia water strider Virus 2 (SxWSV-2)	Unidentified *Gerridae* (B)	Yichang, Hubei (2015^d^)	[90]
Wuchang cockroach virus 1 (WcCV-1)	*Blattella germanica* (B)	Wuhan, Hubei (2015^d^)	[90]
Wuhan insect virus 2 (WhIV-2)	*Hyalopterus pruni* or *Aphelinus* spp. (B)	Wuhan, Hubei (2015^d^)	[90]
Wuhan mosquito virus 1 (WhMV-1-mos)	*Culex tritaeniorhynchus*; *Anopheles sinensis*; *Culex quinquefasciatus* (B)	Wuhan, Hubei; Wenzhou, Zhejiang; Ningbo, Zhejiang (2015^d^)	[90]
Wuhan mosquito virus 2 (WhMV-2-mos)	*Culex tritaeniorhynchus*; *Anopheles sinensis*; *Culex quinquefasciatus*; *Aedes* spp. (B)	Wuhan, Hubei; Wenzhou, Zhejiang; Ningbo, Zhejiang (2015^d^)	[90]
*Phenuiviridae*			
Dabie bandavirus (SFTSV)	Patients (A, B and C)	Xinyang, Henan (2009)	[100]
Hubei diptera virus 3 (HbDV-3)	Diptera (B)	Hubei (2013)	[101]
Hubei diptera virus 4 (HbDV-4)	Diptera (B)	Hubei (2013)	[101]
Hubei diptera virus 5 (HbDV-5)	Diptera (B)	Hubei (2013)	[101]
Hubei lepidoptera virus 1 (HbLV-1)	*Lepidoptera* (B)	Hubei (2013)	[101]
Guertu virus (GTV)	*Dermacentor nuttalli* (A and B)	Tacheng, Xinjiang (2014)	[102]
Bole tick virus 1 (BlTV-1)^b^	*Hyalomma asiaticum* (B)	Boertala, Xinjiang (2015^d^)	[90]
Changping tick virus 1 (CpTV-1) ^b^	*Dermacentor* spp. (B)	Beijing (2015^d^)	[90]
Dabieshan tick virus (DBSH)	*Haemaphysalis Iongicornis* (B)	Huanggang, Hubei (2015^d^)	[90]
Huangpi tick virus 1 (HpTV-1)	*Haemaphysalis doenitzi* (B)	Wuhan, Hubei (2015^d^)	[90]
Huangshi humpbacked fly virus (HsHFV)^b^	Unidentified *Phoridae* (B)	Wuhan, Hubei (2015^d^)	[90]
Lihan tick virus (LITV)	*Rhipicephalus microplus* (B)	Wuhan, Hubei (2015^d^)	[90]
Qingnian mosquito virus (QnMV)^b^	*Culex quinquefasciatus* (B)	Wuhan, Hubei; Wenzhou, Zhejiang (2015^d^)	[90]
Wuhan fly virus 1 (WhFV-1)	*Atherigona orientalis*; *Chrysomya megacephala*; *Sarcophaga* spp.; *Musca domestica* (B)	Jingmen, Hubei (2015^d^)	[90]
Wuhan horsefly virus (WhHV)	Unidentified *Tabanidae* (B)	Wuhan, Hubei (2015^d^)	[90]
Wuhan insect virus 1 (WhIV-1) ^b^	*Asellus* spp.; Unidentified*Nepidae*; *Camponotus japonicus* (B)	Wuhan, Hubei (2015^d^)	[90]
Wuhan louse fly virus 2 (WhLFV-2)^b^	Unidentified *Hippoboscoidea* (B)	Wuhan, Hubei (2015^d^)	[90]
Wuhan millipede virus 1 (WhMV-1-mil)^b^	Unidentified *Polydesmidae* (B)	Beijing; Wuhan, Hubei (2015^d^)	[90]
Wuhan spider virus (WhSV)^b^	*Neoscona nautica*; *Parasteatoda tepidariorum*; *Plexippus setipes* (B)	Wuhan, Hubei; Jingmen, Hubei (2015^d^)	[90]
Wutai mosquito virus (WtMV)	*Culex quinquefasciatus* (B)	Wuhan, Hubei; Wenzhou, Zhejiang (2015^d^)	[90]
Wenzhou shrimp virus 1 (WzSV-1)	*Penaeus monodon* (B)	Wenzhou, Zhejiang (2015^d^)	[90]
Xinzhou mosquito virus (XzMV)^b^	*Anopheles sinensis* (B)	Wuhan, Hubei; Wenzhou, Zhejiang; Ningbo, Zhejiang (2015^d^)	[90]
Yichang insect virus (YcIV)	*Aulacorthum magnoliae* (B)	Yichang, Hubei (2015^d^)	[90]
Yongjia tick virus (YONV)	*Haemaphysalis hystricis* (B)	Wenzhou, Zhejiang (2015^d^)	[90]
Rift Valley fever virus (RVFV)	Imported patient (A and B)	Beijing (2016)	[18]
Wuxiang virus (WUXV)^b^	*Phlebotomus chinensis* (A and B)	Changzhi, Shanxi (2018)	[103]
*Wupedeviridae*			
Wuhan millipede virus 2 (WhMV-2-mil)	Unidentified *Polydesmidae* (B)	Beijing; Wuhan, Hubei (2015^d^)	[90]
Uncharacterized Family			
Jiangxia mosquito virus 1 (JxMV-1)^b^	*Culex tritaeniorhynchus* (B)	Wuhan, Hubei; Ningbo, Zhejiang (2015^d^)	[90]
Jiangxia mosquito virus 2 (JxMV-2)^b^	*Culex tritaeniorhynchus* (B)	Wuhan, Hubei; Ningbo, Zhejiang (2015^d^)	[90]
Shuangao bedbug virus 1 (SgBV-1)^b^	*Cimex hemipterus* (B)	Hong Kong (2015^d^)	[90]
Shuangao mosquito virus (SgMV)^b^	*Armigeres subalbatus* (B)	Wuhan, Hubei (2015^d^)	[90]
Shayang spider virus 2 (SySV-2)^b^	*Neoscona nautica*; *Parasteatoda tepidariorum*; *Pirata* spp.; Unidentified *Araneae* (B)	Wuhan, Hubei; Jingmen, Hubei (2015^d^)	[90]
Wuhan insect virus 3 (WhIV-3) ^b^	*Asellus* spp. (B)	Wuhan, Hubei (2015^d^)	[90]
Wenzhou shrimp virus 2 (WzSV-2) ^b^	*Penaeus monodon*; *Exopalaemon carinicauda* (B)	Wenzhou, Zhejiang (2015^d^)	[90]

“–” The first discovered information of LIJV and RYKV came from GenBank database

^a^Diagnostic methods: (A) isolation of pathogens from samples; (B) molecular detection and sequence determination; (C) serological detection

^b^Not been formally described in taxonomic papers

^c^The first discovered year was taken from the earliest year of sample collection time period

^d^The first dis-covered year was taken from the publication year of references because the sample collection time was not provided

### *Bunyavirales* viruses known to infect humans

In total, 19 viruses belonging to five families were identified to cause human infections, including 5 viruses of *Nairoviridae* [CCHFV, Beiji nairovirus (BJNV), Songling virus (SGLV), Tacheng tick virus 1 (TcTV-1) and Tacheng tick virus 2 (TcTV-2)], 4 viruses of *Hantaviridae* [Haantan virus (HTNV), Seoul virus (SEOV), Imjin virus (MJNV), Puumala virus (PUUV)], 4 viruses of *Peribunyaviridae* [(Batai virus (BATV), Ebinur Lake virus (EBIV), Snowshoe hare virus (SSHV), Tahyna virus (TAHV)], 4 viruses of *Phenuiviridae* [*Dabie bandavirus* (formerly known as SFTS virus, SFTSV), Guertu virus (GTV), RVFV and Wuxiang virus (WUXV)] and 2 viruses of *Arenaviridae* [Lymphocytic choriomeningitis virus (LCMV), Wenzhou virus (WENV)] (Fig. 2A, B). Among them, 10 viruses were the first detected from human infections in the world, including 6 tick-borne viruses (GTV, TcTV-1, TcTV-2, BJNV, SGLV and SFTSV) and one mosquito-borne virus (EBIV), one sandfly-borne virus (WUXV), one rodent-borne virus (WENV) and one shrew-borne virus (MJNV).

From January 1951 to June 2021, there were a total of 447,848 laboratory-confirmed or clinically diagnosed cases with *Bunyavirales* virus infections reported in China. Hantavirus infections (HTNV and SEOV) were the most frequently determined illness with 431,010 cases reported in 31 PLADs of the mainland of China, followed by SFTSV with 16,339 cases in 26 PLADs, CCHFV with 331 cases in Xinjiang Autonomous Region of northwestern China (Fig. 2A). An additional 168 cases were infected by other 7 viruses, including BJNV, SGLV, LCMV, TAHV, TcTV-1, TcTV-2 and RVFV, scattered in 10 PLADs (Fig. 2A). Influenza-like illness (ILI) was the most common symptom, with fever presenting the highest frequency (50.0–100.0%) among human cases infected with *Bunyavirales* viruses with the exception of widely reported human cases of HFRS and SFTS, followed by headache (53.8–98.5%) and fatigue (7.5–52.9%) (Additional file 1: Table S6). Other common unspecific symptoms included gastrointestinal manifestations [e.g., anorexia (0–69.2%), vomiting (4.4–67.5%)] and nausea (2.9–50.0%)] and neurologic manifestations [e.g., mental confusion (2.3–61.8%), depression (0–61.8%) and malaise (0–37.5%)]. Meanwhile, pharyngeal hyperemia (0–58.3%), epistaxis (0–50.4%), myalgia (20.0–39.7%), arthralgia (0–38.5%) and rash or petechiae (0–37.5%) were reported as complications. Among these, there was pharyngeal hyperemia frequently seen in human case infections with TAHV (58.3%), as well as epistaxis in human case infections with CCHFV (39.7–50.4%) (Additional file 1: Table S6). Besides, human infections with the other 8 species of *Bunyavirales* viruses were reported only with serological evidence of specific antibodies, including WENV, MJNV, PUUV, BATV, EBIV, SSHV, GTV and WUXV (Fig. 2B).

### *Bunyavirales* viruses from vectors, wild animals and livestock

**Fig. 3:**
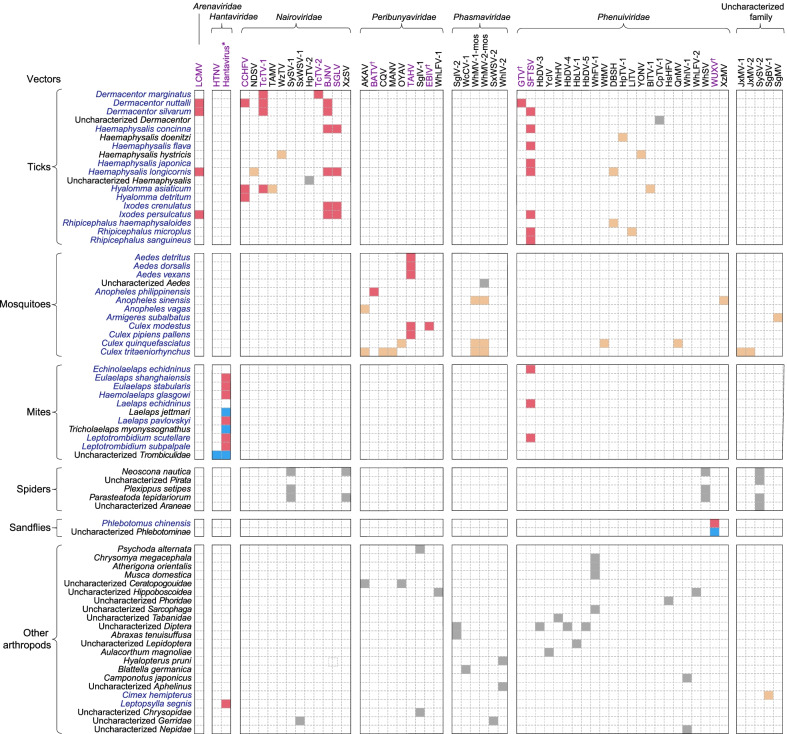
The relationship matrix of *Bunyavirales* virus species and involved vectors. The viruses that could infect humans with any detection method, including molecular, serological and pathogen isolation method, were marked by purple fonts. ^†^This represented infection of human-infected viruses in humans detected only by serological methods. *Hantavirus referred to unclassified hantavirus. Names of vectors were marked in blue if there was any proof through literature or search engines that they bite humans. The red square indicated that viruses can infect humans and can be carried by human-biting vectors, and it turned into blue if the vector cannot bite humans. Light yellow squares indicated viruses were not pathogenic to humans carried by human-biting vectors, and it turned into grey if carried by vectors of non-biting human. *Bunyavirales* viruses in vectors were all detected by molecular methods or pathogen isolation. The full name of each pathogen was shown in Table 1

A total of 52 vector species were reported to carry *Bunyavirales* viruses, with ticks (16 species), mosquitoes (11) and mites (10) acting as the main vectors, followed by spiders (3), sandflies (1) and other vectors (11) (Fig. 3). Among all identified *Bunyavirales* viruses, ticks were also the vectors carrying the most varieties with as many as 18 viruses including 9 of *Nairoviridae*, 8 of *Phenuiviridae* and one of *Arenaviridae*, mainly found in areas of northern, central and eastern China. Eight of these 18 viruses were pathogenic to humans and carried by human-biting ticks (Figs. 2C, D, 3). Mosquitoes were the second most varied, carrying 15 viruses including 7 of *Peribunyaviridae*, 3 of *Phenuiviridae*, 2 of *Phasmaviridae* and 3 of uncharacterized family, mainly found in the central, eastern and southwestern China, of which 3 viruses were pathogenic to humans (Figs. 2C, D, 3). Other common vectors were mites (HTNV, SFTSV and unclassified hantavirus in central China), spiders (SySV-1, SySV-2, WhSV and XzSV in Hubei province) and sandflies (WUXV in Shanxi province) (Figs. 2C, D, 3). Among them, only two viruses pathogenic to humans were identified to be carried by two types of vectors, including hantavirus (mites and fleas) and SFTSV (ticks and mites) (Fig. 3).

A total of 12 orders of wild animals were reported with *Bunyavirales* virus infections, among them Rodentia and Soricomorpha were reported to carry the largest number of viruses (21 and 13 respectively) (Additional file 1: Figs. S6, S7). Rodentia was scattered over the whole nation and Soricomorpha mostly on the east coast of China, both mainly carrying viruses belonging to the family *Hantaviridae*. In addition, 9 species of livestock were determined with *Bunyavirales* virus infections. Cattle and sheep were recorded to carry the largest number of *Bunyavirales* viruses (8 for each), also scattered over the whole nation (Additional file 1: Figs. S6, S7).

### Risk distribution and its drivers of CCHFV

**Fig. 4:**
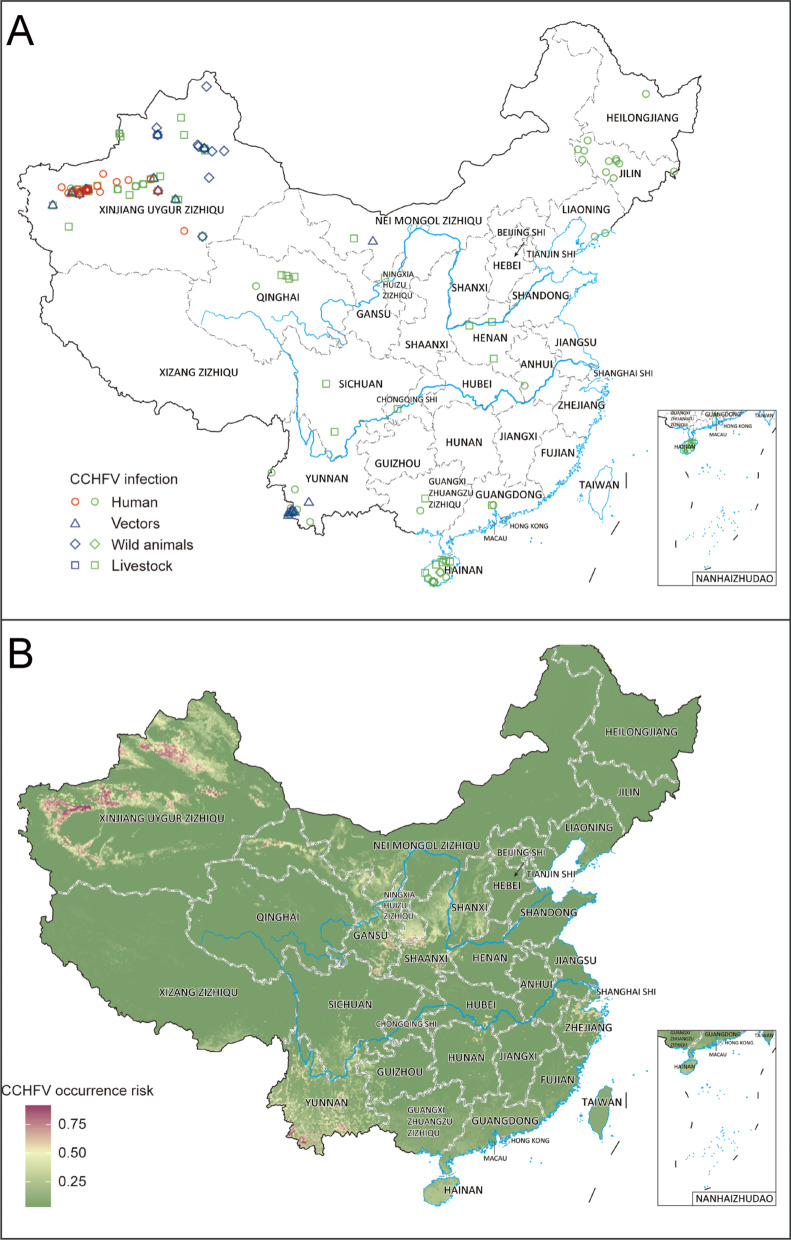
Recorded and predicted risk distribution of CCHFV presence in China. **A** Records of CCHFV were all from literature review. An occurrence record was defined as one or more con-firmed infection(s) with CCHFV at a unique location (the same administrative district or 10 km × 10 km pixel for points) regardless of the type of hosts, detection methods or time points with positive detection. The CCHFV occurrence records detected by serological methods were marked by green, otherwise it turned into red or blue if the records were detected by molecular methods and virus isolation. The coordinates of centroid points were both displayed for administrative district or 10 km × 10 km pixel level records. **B** Predicted risk distribution of CCHFV after averaged 100 boot-strapping BRT models. Source data are provided in Additional file 2

In total, there were 283 occurrence records of CCHFV identified from 14 PLADs, detected in humans, vectors, and animals (Fig. 4A). Human cases were exclusively detected in Xinjiang Autonomous Region, while serosurveys showed a wider distribution, mainly including some areas of northeastern, northwestern and southern China (Fig. 4A). CCHFV detection in vectors was recorded in Xinjiang, Inner Mongolia and Yunnan, while CCHFV detection in animals was scattered in northwestern, central and southern China (Fig. 4A). After the removal of geographic duplication and serological evidence, a total of 27 records of non-serological evidence were used for the environmental niche analyses (Fig. 1, Additional file 2).

Validation statistics indicated strong predictive performance of the BRT models with an average testing area-under-curve (AUC) of 0.963 (Additional file 1: Fig. S8). Model-predicted high-risk areas for CCHFV occurrence were mainly distributed in Xinjiang Autonomous Region, with more focalized areas at high risk in southwest border areas of Yunnan province (Fig. 4B). There were medium-risk areas distributed in central China, stretched over Gansu, Ningxia, and Shaanxi, as well as in eastern China (Zhejiang province) and southwestern China (Yunnan province). Our models showed the probability of CCHFV presence to be particularly determined by the predicted probability of *H. asiaticum* presence, which contributed 68.6% to the occurrence of CCHFV in the model (Table 1). Additional important drivers included annual mean temperature and precipitation seasonality (both relative contributions > 5%). Effect plots for each covariate in the model were provided in Additional file 1: Fig. S9. The model-predicted high-risk areas of CCHFV presence were more extensive than those recorded, with the areas at risk increasing from 2700 to 1,305,500 km^2^ and the population size at risk increasing from 0.3 million to 135.1 million people.

### Monthly potential risk distribution and its drivers of RVFV

A total of 326 publications were included in the study, yielding 2961 records of RVFV occurrence. An additional 1059 records of confirmed human or animal cases were appended from the EMPRES-i dataset (Additional file 1: Fig. S1). Finally, there were 4020 records identified from 39 countries, and the specific locations with RVFV detection were mapped (Additional file 1: Fig. S10A). After the removal of geographic duplication and serological records, monthly distributions (1193 records) were further mapped and used to conduct the ecological niche model to assess the monthly ecological suitability for RVFV occurrence (Additional file 1: Fig. S10B, Additional file 3). The BRT models with an average testing AUC of 0.956 indicated a decent predictive power (Additional file 1: Fig. S11). Higher livestock density and more precipitation in the previous month were the two most influential covariates, respectively contributing 21.6% and 21.2% to the model (Table 1, Additional file 1: Fig. S12). Effect plots for each covariate in the model and monthly model-predicted risk in China were shown in Additional file 1: Figs. S12–14.

**Fig. 5:**
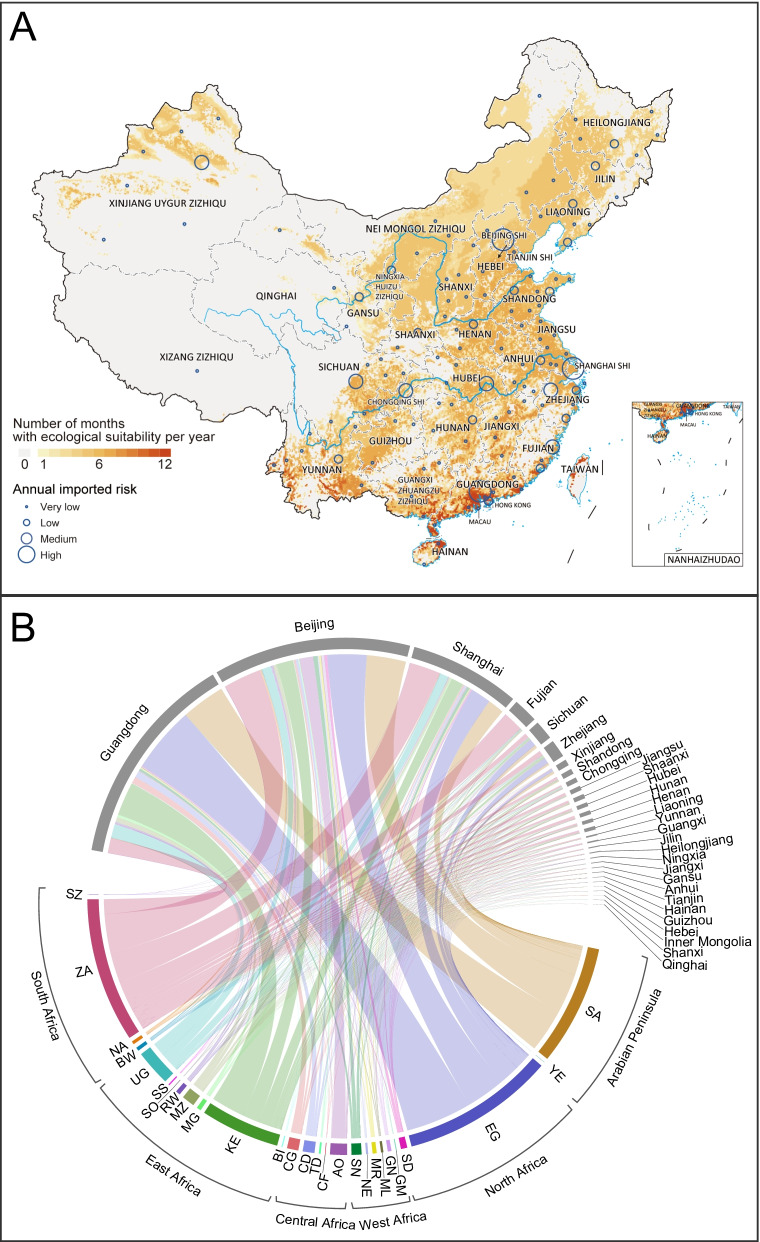
Average number of suitable months per year and annual imported risk for RVFV. **A** The average number of suitable months per year across years 2000–2020 was shown. Places in red were suitable for more months per year, on average. The annual imported risk index was calculated by adding the monthly imported risk index at the city level. The annual city imported risk level was classified into four categories: very low (imported risk index ≤ 10^–2^); low (imported risk index between 10^–2^ and 10^–1^); medium (imported risk index between 10^–1^ and 1); high (imported risk index > 1). **B** The annual imported risk was from the infected countries in RVFV epidemic area to the provinces in China. The annual imported risk index at the provincial level was calculated by adding the annual imported risk index of cities in the same province. The following are the full names of RVFV infected countries: SZ (Swaziland); ZA (South Africa); NA (Namibia); BW (Botswana); UG (Uganda); SS (South Sudan); SO (Somalia); RW (Rwanda); MZ (Mozambique); MG (Madagascar); KE (Kenya); BI (Burundi); CG (Republic of Congo); CD (Democratic Republic of the Congo); TD (Chad); CF (Central African Republic); AO (Angola); SN (Senegal); NE (Niger); MR (Mauritania); ML (Mali); GN (Guinea); GM (Gambia); SD (Sudan); EG (Egypt); YE (Yemen); SA (Saudi Arabia). Source data are provided in Additional file 2

According to the projection of the BRT models and the imported risk assessment, spatial and temporal variation of ecological suitability for RVFV transmission and its imported risk were evident in China (Additional file 1: Figs. S13–14). Guangzhou city had high environmental suitability of RVFV transmission all year round with the highest imported risk potentially originating in Egypt, Saudi Arabia and Kenya (Fig. 5). Although Beijing and Shanghai had high imported risk throughout year, only the environmental and climatical conditions from May to November were suitable for RVFV transmission (Additional file 1: Figs. S13–14). It is worth noting that some areas of northern China showed high environmental suitability between June and October, e.g., northern Xinjiang from July to September and areas centered around eastern Inner Mongolia and stretched over Shanxi, Shaanxi and Ningxia (Additional file 1: Figs. S13–14). Northern Xinjiang had a relatively high imported risk throughout the year from Egypt and Saudi Arabia, while the imported risk of Inner Mongolia and its surrounding areas was quite low. Broad areas of western China such as the Qinghai-Tibet region were shown to be unsuitable for the transmission of RVFV throughout the year, and there was almost no imported risk. In general, southern and eastern China generally had more suitable environmental conditions for RVFV transmission than northern and western China (Fig. 5, Additional file 1: Figs. S13–14). The model-predicted areas at risk of RVFV presence reached over 4,000,000 km^2^ from July to September in China and the population size at risk reached over 1000 million people from June to October (Additional file 1: Table S6).

## Discussion

In this study, we conducted by far the most comprehensive description of 89 viruses belonging to the order *Bunyavirales* in China for the first time, and showed a compendium of the distribution of viruses in space, including infections in humans, vectors and animal hosts. Furthermore, we made risk predictions and assessments of two major neglected *Bunyavirales* viruses including CCHFV and RVFV. The two viruses, both had the potential for outbreaks in local areas of China, and the findings of the predicted hotspots and seasonality at risk were helpful for their efficient surveillance.

Our study showed that the highest richness of *Bunyavirales* viruses was mainly distributed in Hubei, Zhejiang, Yunnan provinces and Xinjiang Autonomous Region. The spatial pattern of pathogen species usually depends on the distribution of their vectors and hosts and the ecology of the transmission. In particular, the distribution of vector organisms can directly affect their contact with animal hosts and humans, which in turn affect the spatial spread of pathogens [32]. The survey study in four regions of China, including Hubei and Zhejiang provinces, showed a high richness of arthropods, including 70 arthropod species carrying 41 newly-identified species of *Bunyavirales* viruses by RNA sequencing [33]. In addition, Xinjiang and Yunnan were the PLADs with the richest species of ticks [29]. Yunnan and Hubei provinces had abundant diversity of mosquito species [34]. This evidence indicated that distribution of pathogens had an overall correlation with the ecogeographical faunal region for specific vectors [35].

Ticks carried the highest variety of *Bunyavirales* viruses, followed by mosquitoes, thus highlighting the critical role of ticks and mosquitoes in harboring and disseminating *Bunyavirales* viruses. Compared with mosquitoes, there were noticeable geographic clusters for ticks carrying *Bunyavirales* viruses, especially in northeastern China, in Xinjiang and Shandong. There were the most abundant tick species (54 species) in Xinjiang [29], and high-density ticks in northeastern China due to the high forest cover [36]. These factors may increase the chance of ticks biting and exposure to the viruses in animals and humans, and promote the transmission cycle of *Bunyavirales* viruses. Shandong province is located in the North China Plain with more grassland vegetation and rich plant and animal resources. Shandong province was proved to be a high-suitable area for *Haemaphysalis longicornis* presence, which was the most important vector of SFTSV in China [11]. In addition to Shandong, the high-suitable areas of *H. longicornis* were mainly concentrated in Liaoning province of northeastern China, Jiangsu and Zhejiang provinces in the eastern coastal areas, and some areas in central China, which was also consistent with the geographical distribution of SFTSV [11]. Therefore, it is necessary to focus on the hotspot of ticks in China and increase the monitoring of ticks and tick-borne *Bunyavirales* viruses. The distribution of mosquitoes carrying *Bunyaviridae* viruses was relatively scattered, except in northeastern and southern China. With global warming, mosquitoes have longer life cycle and larger range of activities. In addition, the risk of mosquito-borne infectious diseases is also increasing due to the increase of population density due to rapid urbanization and the frequent population flow under economic globalization, displaying the need for the monitoring of mosquitoes and mosquito-borne viruses.

There were also significant differences in the types of viruses carried by different vectors, among which *Nairoviridae* and *Phenuiviridae* were mainly carried by ticks, *Peribunyaviridae* mainly carried by mosquitoes, and *Hantaviridae* and *Arenaviridae* mainly carried by Rodentia. Hantavirus and SFTSV have been determined from over two vector types, which means that both have more potential for transmission, more difficulties to control, and higher risks to humans, showing a greater need for their consideration. In addition, we found that the *Bunyavirales* viruses carried by ticks were not detected in mosquitoes, and the converse was also true. One study examined the putative receptor-binding domains of tick-borne and mosquito-borne flaviviruses, the envelope (E) protein domain III (D3), and found that tick-borne recombinant D3s (rED3s) exhibited different spectral properties distinct from mosquito-borne viruses, that tick-borne viruses are more stable than mosquito-borne viruses [37]. Flaviviruses and bunyaviruses were both icosahedral, membrane-containing plus-stranded RNA viruses, and the respective glycoproteins E and Gc of each encoded the same basic protein fold [38]. Thus, we speculated that there might be some differences in biophysical properties resulting in different biological properties and “mutual exclusion” between tick-borne and mosquito-borne bunyaviruses, but further research was needed to determine the differences. If “mutual exclusion” for viruses carried by ticks and mosquitoes could be determined, it would be informative for precisely targeting direction in surveillance of main vectors, e.g., paying little attention to the mosquito vectors when monitoring CCHFV carried by ticks.

The viruses belonging to the order *Bunyavirales* have caused a variety of diseases and attracted much attention. Our results showed that except for HFRS and SFTS, the two widely distributed infectious diseases caused by *Bunyavirales* viruses in China, other *Bunyavirales* virus-related infectious diseases were mainly distributed in Xinjiang, northeastern and central China (Fig. 2A). Human cases of CCHF occurred first in 1965 in China with a very high fatality rate [39]. Recently, CCHF outbreaks continued worldwide, e.g., in Turkey in 2021 and Mali in 2020 [40, 41]. However, no human indigenous cases of CCHF were reported in China since 2003 [42]. CCHF is not included in CISDCP, and CCHFV has still been detected in animals and vectors in recent years in China [30, 43–45], which indicates a neglected possibility and a high reemerging risk of this disease exist, especially in the potential hot-spot areas of CCHFV occurrence predicted by this study. Our study not only included meteorological and land cover covariates considered in previous studies [15, 17], but also included the influencing covariates concerning host and vector distribution, which reflected their roles in the biological transmission of CCHFV. Besides two areas in Xinjiang and southwestern Yunnan with high environmental suitability of CCHFV occurrence, our study identified some unneglectable medium-risk areas in central China as well as in eastern China, which were largely due to the distribution of *H. asiaticum* tick. Our BRT models showed that the distribution of *H. asiaticum* contributed significantly to CCHFV occurrence, further corroborating the vital role of this tick in maintaining and transmitting the disease pathogens in the natural environment. Therefore, intensive surveillance for potential CCHFV spread or invasion events is needed in the *H. asiaticum* rich areas. In addition, we also noted that there was some serological evidence of human infections in Hainan province and northeastern China where low risk was predicted (Fig. 4). Considering that most evidence occurred in the 1990s, there might be cross immune reaction in this serological evidence [46], so it would be prudent to strengthen the search for CCHFV in these areas to further evaluate the risk.

As for RVFV in China, we analyzed seasonal dynamics of the environmental suitability of RVFV occurrence and assessed the imported risk of RVFV for the first time, indicating the potential hotspots of RVFV occurrence that needed to be monitored. There was high environmental suitability of RVFV occurrence in Guangzhou city all year round, Beijing and Shanghai from May to November, and northern Xinjiang from July to September. A previous study showed high risk of RVFV occurrence in the southwest border region of China without considering the seasonality [47]. However, our data showed a very low environmental suitability of RVFV occurrence in Tibet in southwestern China. In addition, the imported RVFV case in Beijing was consistent with our data that the imported risk of RVFV-infected cases was high from Angola to Beijing, rather than Guangdong, Shanghai or other cities (Fig. 5B) [18]. Our data showed that the imported risk existed due to the population movement from Egypt, South Africa, Saudi Arabia and Kenya to China, which needed to be closely monitored. Our results can help optimize the allocation of surveillance and control of RVFV where the imported risk of RVFV is high. The BRT models showed that livestock density was the most important covariate contributing to RVFV occurrence with a generally positive correlation. Except for mosquito bites, human infections with RVFV can be through direct contact with tissue, blood and secretions of infected animals, which is more common in areas with high livestock density [21, 48]. In addition, sufficient rainfall and warm temperature are the other two important environmental conditions for RVFV transmission. Prolonged periods of sufficient rainfall provide breeding sites for RVFV vectors, such as the infected and transovarial mosquitoes, which are also facilitated by warm temperatures [22]. Earlier viral dissemination and higher transmission speed of RVFV would result from warmer temperatures [47, 49–51]. Therefore, it is essential for local governments to enhance the surveillance of the sanitary conditions and the control of mosquitos, especially in the areas with high-density livestock, sufficient rainfall, and warm temperature, as well as providing context-specific education, such as the necessity of avoiding contact with bodily fluids from potentially infected livestock.

It is worth emphasizing that of the human cases included in our study, there were 5 viruses reported only once in China, including BJNV (68 cases) [52], SGLV (43 cases) [53], TAHV (13 cases) [54], TCTV-1 (1 case) [55] and TCTV-2 (1 case) [56], indicating the necessity of further surveillance and investigation about the human pathogenicity of these viruses. On the other hand, some *Bunyavirales* viruses included in our study had no evidence of infecting humans yet. Owing to their high rates of nucleotide substitution and poor mutation error-correction ability [57–63], they were possible to be neglected due to limited surveys and laboratory tests of them in humans, or they would adapt to new hosts including humans. For example, TcTV-1 and TcTV-2 were both first detected from ticks (*Dermacentor marginatus*) in 2015 [33], while human cases infected with TcTV-1 and TcTV-2 were first found in 2020 and 2021, respectively [55, 56]. Especially, the five viruses including Amur virus (AMRV) [64], Cat Que virus (CQV) [65], Nairobi sheep disease virus (NDSV) [66], Oya virus (OYAV) [67] and Tamdy virus (TAMV) [68] had been reported to infect humans outside China, indicating the necessity of intensive etiological surveillance in humans.

Our study has some limitations. Although we collected corresponding data from CISDCP and literature as comprehensively as possible, the included data spanned a long duration of more than 60 years. Some historical documents inevitably suffered from flaws of changing criteria or standards that were applied to the data. The quality of detection and reports of *Bunyavirales* virus infections were also inconsistent by region due to different methods of diagnosis, limited resources for diagnostic testing and the variable reporting capacities of local health systems. Therefore, the distribution can be regarded as general epidemic trends of *Bunyavirales* viruses, but cannot be interpreted as an accurate reflection of the underlying distribution of vector-borne viruses or the prevalence. Some positive results of serological tests included in our study may be affected by the existence of immunological cross-reaction between species of *Bunyavirales* viruses, and diverse robust detection methods were needed to confirm this serological evidence, such as molecular detection and virus isolation. In addition, the ecological modeling only considered the presence or absence of test-positive human, animal and vector samples, ignoring the frequency of samples that might provide additional information about ecological suitability for persistence.

## Conclusions

Our study provides a full picture of the distribution of all *Bunyavirales* viruses detected in China, which can help to understand the diversity of *Bunyavirales* viruses, the infection spectrum in vectors and animals, as well as the potential transmission risk and disease burden caused by them. For two specific possible re-emerging but usually neglected viruses in China (CCHFV and RVFV), we updated the knowledge regarding their ecological niche and distribution, and for the first time mapped the seasonally potential-risk areas of RVFV occurrence. The risk maps created by this study could guide future refinement of surveillance of CCHFV and RVFV in humans, vectors and animals, especially at targeted high-risk times and regions, and provide guidance for when and where the local government should remain vigilant for potential spread or imported events.

## Supplementary Information

**Additional file 1.** Additional information includes Methods, 14 Figures, 6 Tables and References. **Table S1.** The specific references for all 89 *Bunyavirales* viruses in China and Rift Valley fever virus in Africa and the Arabian Peninsula. **Table S2.** Search terminology used for the systematic review. **Table S3.** Information of explanatory covariates sources in this study. **Table S4.** Description of 42 potential explanatory covariates used in the modelling efforts. **Table S5.** Clinical characteristics of human cases infections with different viruses (species) belonging to the order *Bunyavirales* in China. **Table S6.** The model-predicted areas and population sizes at risk by monthly for RVFV occurrence in China. **Fig. S1.** The flow diagram of data collection for Rift Valley fever virus. **Fig. S2.** Recorded and predicted risk distribution of *H. asiaticum* presence in China. **Fig. S3.** Correlation matrix for 36 covariates in CCHFV model. **Fig. S4.** Correlation matrix for 18 covariates in RVFV model. **Fig. S5.** Distribution of the first identification site of 89 *Bunyavirales* viruses in China. **Fig. S6.** Geographical distributions of *Bunyavirales* viruses detected in animals. **Fig. S7.** The relationship matrix of *Bunyavirales* virus species and involved animals. **Fig. S8.** ROC curves and AUC values of CCHFV model. **Fig. S9.** The marginal effect of explanatory covariates for CCHFV occurrence risk in the model. **Fig. S10.** Recorded distribution of RVFV occurrence in Africa and the Arabian Peninsula. **Fig. S11.** ROC curves and AUC values of RVFV model. **Fig. S12.** The marginal effect of explanatory covariates for RVFV occurrence risk in the model. **Fig. S13.** Mean environmental suitability and monthly imported risk for RVFV from January to June in China. **Fig. S14.** Mean environmental suitability and monthly imported risk for RVFV from July to December in China. 

**Additional file 2.** The dataset for CCHFV records in China used for BRT models.

**Additional file 3.** The dataset for RVFV records from 2000 to 2020 in Africa and the Arabian Peninsula used for BRT models.

## Data Availability

The datasets supporting the conclusions of this article are included within the article and Additional file 2 and Additional file 3.

## Abbreviations

HFRS: Hemorrhagic fever with renal syndrome
HCPS: Hantavirus cardiopulmonary syndrome
SFTS: Severe fever with thrombocytopenia syndrome
SFTSV: Severe fever with thrombocytopenia syndrome virus
CCHF: Crimean-Congo hemorrhagic fever
CCHFV: Crimean-Congo hemorrhagic fever virus
XHF: Xinjiang hemorrhagic fever
RVF: Rift Valley fever
RVFV: Rift Valley fever virus
CISDCP: China Information System for Disease Control and Prevention
GIDEON: Global Infectious Diseases and Epidemiology Network
CNKI: China National Knowledge Infrastructure
EMPRES-i: Global Animal Disease Information System
NDVI: Normalized Difference Vegetation Index
IATA: International Air Transport Association
BRT: Boosted regression tree
AUC: Average testing area-under-curve
ICTV: International Committee on Taxonomy of Viruses
NCBI: National Center for Biotechnology Information
