# Supplementary material for: Mapping the viruses belonging to the order Bunyavirales in China
Source: Infect Dis Poverty. 2022 Jul 7;11:81. doi: 10.1186/s40249-022-00993-x (PMC9264531; doi:10.1186/s40249-022-00993-x)
Supplement: Supplementary file 1 — Additional file 1. Additional information includes Methods, 14 Figures, 6 Tables and References. Table S1. The specific references for all 89 Bunyavirales viruses in China and Rift Valley fever virus in Africa and the Arabian Peninsula. Table S2. Search terminology used for the systematic review. Table S3. Information of explanatory covariates sources in this study. Table S4. Description of 42 potential explanatory covariates used in the modelling efforts. Table S5. Clinical characteristics of human cases infections with different viruses (species) belonging to the order Bunyavirales in China. Table S6. The model-predicted areas and population sizes at risk by monthly for RVFV occurrence in China. Fig. S1. The flow diagram of data collection for Rift Valley fever virus. Fig. S2. Recorded and predicted risk distribution of H. asiaticum presence in China. Fig. S3. Correlation matrix for 36 covariates in CCHFV model. Fig. S4. Correlation matrix for 18 covariates in RVFV model. Fig. S5. Distribution of the first identification site of 89 Bunyavirales viruses in China. Fig. S6. Geographical distributions of Bunyavirales viruses detected in animals. Fig. S7. The relationship matrix of Bunyavirales virus species and involved animals. Fig. S8. ROC curves and AUC values of CCHFV model. Fig. S9. The marginal effect of explanatory covariates for CCHFV occurrence risk in the model. Fig. S10. Recorded distribution of RVFV occurrence in Africa and the Arabian Peninsula. Fig. S11. ROC curves and AUC values of RVFV model. Fig. S12. The marginal effect of explanatory covariates for RVFV occurrence risk in the model. Fig. S13. Mean environmental suitability and monthly imported risk for RVFV from January to June in China. Fig. S14. Mean environmental suitability and monthly imported risk for RVFV from July to December in China. [file 40249_2022_993_MOESM1_ESM.docx]

**Supplementary information**

Supplementary Material to: Mapping the viral pathogens belonging to the order *Bunyavirales* in China

**Table of Contents**

| **Page** | **Item** |
| --- | --- |
| 2 | Supplementary Methods |
| 6 | Table S1: The specific references for all 89 *Bunyavirales* viruses in China and Rift Valley fever virus in Africa and the Arabian Peninsula. |
| 8 | Table S2: Search terminology used for the systematic review. |
| 9 | Table S3: Information of explanatory covariates sources in this study. |
| 10 | Table S4: Description of 42 potential explanatory covariates used in the modelling efforts. |
| 11 | Table S5: Clinical characteristics of human cases infections with different viruses (species) belonging to the order *Bunyavirales* in China. |
| 12 | Table S6: The model-predicted areas and population sizes at risk by monthly for RVFV occurrence in China. |
| 13 | Fig. S1: The flow diagram of data collection for Rift Valley fever virus. |
| 14 | Fig. S2: Recorded and predicted risk distribution of *H. asiaticum* presence in China. |
| 15 | Fig. S3: Correlation matrix for 36 covariates in CCHFV model. |
| 16 | Fig. S4: Correlation matrix for 18 covariates in RVFV model. |
| 17 | Fig. S5: Distribution of the first identification site of 89 *Bunyavirales* viruses in China. |
| 18 | Fig. S6: Geographical distributions of *Bunyavirales* viruses detected in animals. |
| 19 | Fig. S7: The relationship matrix of *Bunyavirales* virus species and involved animals. |
| 20 | Fig. S8: ROC curves and AUC values of CCHFV model. |
| 21 | Fig. S9: The marginal effect of explanatory covariates for CCHFV occurrence risk in the model. |
| 22 | Fig. S10: Recorded distribution of RVFV occurrence in Africa and the Arabian Peninsula. |
| 23 | Fig. S11: ROC curves and AUC values of RVFV model. |
| 24 | Fig. S12: The marginal effect of explanatory covariates for RVFV occurrence risk in the model. |
| 25 | Fig. S13: Mean environmental suitability and monthly imported risk for RVFV from January to June in China. |
| 26 | Fig. S14: Mean environmental suitability and monthly imported risk for RVFV from July to December in China. |
| 27 | Supplementary References |

**Supplementary Methods**

***Data on Bunyavirales viruses***

A standardized form was used to collate data on the following variables: year of publication, study year, study subjects (human beings, vectors or animals), laboratory method (molecular, serological or isolation assay) used to identify viral pathogens belonging to the order *Bunyavirales*, the number of samples tested and positive case, geo-referenced study sites, and the baseline information on the study subjects. Since the virus names and taxonomy might have changed along the study years, online taxonomy search websites, including International Committee on Taxonomy of Viruses (ICTV) (https://talk.ictvonline.org/) and National Center for Biotechnology Information (NCBI) Taxonomy (https://www.ncbi.nlm.nih.gov/taxonomy/) were used to update the current taxonomy classification. All the human cases had been confirmed by laboratory test methods (molecular detection, virus isolation, microscopy, a four-fold increase in titer of specific antibodies or seroconversion)[1]. Besides, the report data from the China Information System for Disease Control and Prevention (CISDCP) including laboratory-confirmed cases and clinically diagnosed cases without specified the type of test or diagnosis used, was considered as confirmed cases, and no genospecies were shown on these data. The database was to summarize the distribution of *Bunyavirales* viruses in China by location and different hosts or vectors (humans, animals, and vectors).

The data on RVFV occurrence in RVF epidemic areas were mainly extracted from two sources, including (1) the report data of RVF human and animal cases confirmed by regional partner laboratories, from the Global Animal Disease Information System (EMPRES-i) database of the United Nations Food and Agriculture Organization (FAO) (http://empres-i.fao.org/eipws3g/) between January 2004 and June 2021, (2) literature reporting the RVFV occurrence in humans, animals and vectors, published between January 1955 and June 2021. Literature related to RVFV was searched from the major electronic databases (PubMed, GIDEON, and Web of Science), using the following search terms “(Rift Valley Fever [Title/Abstract]) OR (Rift Valley fever virus [Title/Abstract])”, and the detailed procedures of the literature review were similar with which was mentioned above (Table S1, Fig. S1). We also checked the references in our study by comparison with the study by Hardcastle *et al.* [2] to identify any missing relevant articles. The details of the data extraction and management were introduced in Fig. S1.

After data collection was completed, all data were carefully checked, and duplicate data were removed. Then all data was geo-referenced by using Baidu Map (http://api.map.baidu.com/lbsapi/getpoint/), which generated point-level data at village or town level and polygon-level data at county, prefecture, or province level.

***Data on environmental, ecoclimatic and biological covariates***

Multi-source data on explanatory covariates which possibly contributed to diffusion and persistence of CCHFV[3-7] and RVFV[2, 8-11] were collected (Table S3). For meteorological data, on the one hand, we collected monthly minimum temperature, maximum temperature and precipitation from WorldClim (<https://www.worldclim.org/>), and used to create 19 cross-sectional ecoclimatic covariates (Bio01-19), which could reflect the average level of temperature and precipitation that differed marginally across seasons; on the other hand, we supplemented monthly 2m temperature and total precipitation from European Centre for Medium-Range Weather Forecasts (ECMWF, <https://cds.climate.copernicus.eu/cdsapp#!/home>). At the same time, data on volume of water in soil and surface runoff by monthly were also derived from ECMWF, which were measures of the availability of water in the soil, and could be used as indicators of drought or flood. Global planar river system data was from the Data Center for Resources and Environmental Sciences (RESDC, <http://www.resdc.cn/Default.aspx>), and used to create the minimum distance to nearest bodies of water. The levels of elevation were obtained from EarthEnv-DEM90 digital elevation model Dataset **(**<http://www.earthenv.org/>). Data on land cover came from European Space Agency (ESA, <https://maps.elie.ucl.ac.be/CCI/viewer/index.php>), which included the coverage of agriculture, forest, grassland, shrubland, sparse vegetation, wetland, water, bare and urban areas. NDVI, from NASA Earth Observations (NEO, <https://neo.sci.gsfc.nasa.gov/>), was correlated with increased vectors and reservoir hosts suitability and played important roles in shaping the ecological niches of them. In order to obtain the geographic distribution of potential reservoir hosts, livestock density was collected from Food and Agriculture Organization of the United Nations (FAO, <http://www.fao.org/livestock-systems/en/>). Mammalian richness was from Socioeconomic Data and Applications Center (SEDAC, <https://sedac.ciesin.columbia.edu/>). We also took population density from LandScan (<https://www.satpalda.com/product/landscan/>) into account, owing to the impact of human activities. Besides, we collected the distribution of *Hyalomma asiaticum* (*H. asiaticum*), for the vector were associated with the transmission of CCHFV in China. Data on *H. asiaticum* were collected from the literature review.

At the study area (China, or Africa and the Arabian Peninsula), land cover data were processed by using “Tabulate Area” in ArcGIS 10.7 into raster maps at a resolution of 10km × 10km grid as well as at the administrative area level to obtain the proportion of each land cover type in the unit area. Meanwhile, the data of minimum distance to nearest bodies of water was calculated by using “Near Analysis” in ArcGIS 10.7 and the method was selected as “GEODESIC”. Other variables were processed by using “Zonal Statistics as Table” and “statistics_type” was selected as “MEAN”. Then we obtained 42 variables from all data sources (Table S4).

***Predict the probability of Hyalomma asiaticum presence in China***

As *H. asiaticum* is a major vector of the CCHFV in China, we collected the occurrence records of *H. asiaticum* from a previous database by Zhao *et al.*[12], and added detailed location information of records. The boosted regression tree (BRT) model was used to map the predicted probabilities of *H. asiaticum* presence. Each occurrence record was geo-referenced as point data or polygon data and correlated with socio-environmental covariates, just like CCHFV occurrence records processing. After geographic deduplication of presence point, background points were generated by randomly sampling at the 10km × 10km grid level, and the number of extractions was three times as much as the number of presence point. As for socio-environmental covariates (Table S4), ten variables (annual mean temperature, agriculture, grassland, total precipitation, precipitation seasonality, elevation, cattle, sparse vegetation, sheep, population density) were included in the final model after correlation analysis and pilot model. The BRT model results showed predictions with the average testing AUC of 0.940, and the recorded locations and model-predicted distribution probability of *H. asiaticum* were shown in Fig. S2.

***Monthly explanatory covariates selection***

Due to mosquitoes and other RVFV vectors were present on every continent except Antarctica[13], We use meteorological and other variables to indirectly identify areas in space and time that could serve as suitable mosquito habitats instead of model-predicted distribution probability of RVFV vectors. A distinguishing feature of RVF environmental epidemiology is that it occurred after heavy rainfall, because standing water could induce the hatching mosquitoes and served as a habitat for new generations and other species of mosquitoes[2]. Therefore, mean precipitation in previous month, volume of water in soil in previous month, surface runoff in previous month, and minimum distance to nearest bodies of water were considered as important influence variables (Table S4). Mean temperature in previous month was also included in the model, considering that mosquitoes survival from eclosion to adult emergence was relatively low below 15°C[14]. Considering the geographic difference between the modeled area (RVF epidemic area) and the target prediction area (China), the same type of livestock actually varies in the living habits, so we merged every type of livestock density and included into the model.

***Model development***

In all BRT models, a bootstrapping procedure with 100 iterations was conducted to generate more robust estimates. In each iteration, a training set with 80% of data was randomly selected by bootstrapping without replacement, and the remaining 20% served as a test set. The train set was used to train the BRT model, and the test set was used to evaluate model performance and generate the best threshold cutoff value based on the Youden index for the classification of predicted values. The algorithm hyper-parameters were set to the following values: cross-validation folds=10, tree complexity=5, learning rate=0.005, and bag fraction=0.75.

***RVFV imported risk assessment***

The monthly risk index ($r$) of RVFV population at risk importing to a city in China ($k$) from one RVFV infected country ($j$) was defined as: the risk flow from $j$ to $k$ as the matrix

$r_{jk}=\frac{t_{jk}}{{Pop}_{j}}\sum_{i} {Pr}_{i}{Pop}_{i}$.

The risk of population at risk importing to $k$ from whatever origin in RVFV infected countries was then

$R_{k}=\sum_{j} r_{jk}$.

where $i$ was a pixel in country $j$; ${Pr}_{i}$ was the predicted probability of a pixel in country $j$; ${Pop}_{i}$ was the population of a pixel in country $j$; $t_{jk}$ was the monthly travel flux from country $j$ to city $k$; ${Pop}_{j}$ was the total population in country $j$.

This risk is normalized so that $\sum_{k} R_{k}=1$.

**Table S1: The specific references for all 89 *Bunyavirales* viruses in China and Rift Valley fever virus in Africa and the Arabian Peninsula.**

| Region | Family | Virus | Abbreviation of virus | References |
| --- | --- | --- | --- | --- |
| China | *Arenaviridae* | Lymphocytic choriomeningitis virus | LCMV | [15-31] |
|  |  | Ryukyu virus | RYKV | [32] |
|  |  | Wenzhou virus | WENV | [33-40] |
|  |  | Alxa virus | ALXV | [41] |
|  |  | Lijiang virus | LIJV | [32] |
|  | *Hantaviridae* | Hantaan virus | HTNV | [42-106] |
|  |  | Seoul virus | SEOV | [42, 44-46, 49, 50, 52, 53, 56-60, 67, 68, 71, 72, 76, 78, 79, 83, 85-87, 89, 91, 92, 94, 96, 98, 99, 103, 105-162] |
|  |  | Xinyi virus | XYIV | [163] |
|  |  | Dabieshan virus | DBSV | [88, 104, 164-170] |
|  |  | Fusong virus | FUSV | [171, 172] |
|  |  | Yuanjiang virus | YUJV | [171] |
|  |  | Puumala virus | PUUV | [63, 173-183] |
|  |  | Khabarovsk virus | KHAV | [69, 172] |
|  |  | Amur virus | AMRV | [184-186] |
|  |  | Qian Hu Shan virus | QHSV | [187] |
|  |  | Cao Bang virus | CBNV | [163] |
|  |  | Luxi virus | LUXV | [188, 189] |
|  |  | Yakeshi virus | YKSV | [190] |
|  |  | Thottapalayam virus | TPMV | [191, 192] |
|  |  | Lianghe virus | LHEV | [190] |
|  |  | Longquan virus | LQUV | [190] |
|  |  | Imjin virus | MJNV | [131, 191, 193] |
|  |  | Fugong virus | FUGV | [194] |
|  |  | Huangpi virus | HUPV | [190] |
|  |  | Kenkeme virus | KKMV | [69] |
|  |  | Laibin virus | LAIV | [195, 196] |
|  |  | Xuan son virus | XSV | [195] |
|  |  | Uncharacterized hantavirus | Uncharacterized HV | [49, 89, 94, 129, 136, 155, 159, 197-269] |
|  | *Nairoviridae* | Crimean-Congo hemorrhagic fever virus | CCHFV | [270-314] |
|  |  | Nairobi sheep disease virus | NDSV | [315, 316] |
|  |  | Huangpi tick virus 2 | HpTV-2 | [317] |
|  |  | Tacheng tick virus 1 | TcTV-1 | [317, 318] |
|  |  | Tacheng tick virus 2 | TcTV-2 | [317, 319] |
|  |  | Sanxia water strider virus 1 | SxWSV-1 | [317] |
|  |  | Shayang spider virus 1 | SySV-1 | [317] |
|  |  | Wenzhou tick virus | WzTV | [317] |
|  |  | Xinzhou spider virus | XzSV | [317] |
|  |  | Beiji nairovirus | BJNV | [320] |
|  |  | Songling virus | SGLV | [321] |
|  |  | Tamdy virus | TAMV | [322] |
|  | *Peribunyaviridae* | Snowshoe hare virus | SSHV | [271, 323-325] |
|  |  | Akabane virus | AKAV | [326-338] |
|  |  | Batai virus | BATV | [339-345] |
|  |  | Cat Que virus | CQV | [346] |
|  |  | Tahyna virus | TAHV | [325, 347-354] |
|  |  | Manzanilla virus | MANV | [355] |
|  |  | Oya virus | OYAV | [338, 356] |
|  |  | Ebinur Lake Virus | EBIV | [357, 358] |
|  |  | Shuangao insect virus 1 | SgIV-1 | [317] |
|  |  | Wuhan louse fly virus 1 | WhLFV-1 | [317] |
|  | *Phasmaviridae* | Shuangao Insect Virus 2 | SgIV-2 | [317] |
|  |  | Sanxia water strider Virus 2 | SxWSV-2 | [317] |
|  |  | Wuchang cockroach virus 1 | WcCV-1 | [317] |
|  |  | Wuhan insect virus 2 | WhIV-2 | [317] |
|  |  | Wuhan mosquito virus 1 | WhMV-1-mos | [317] |
|  |  | Wuhan mosquito virus 2 | WhMV-2-mos | [317] |
|  | *Phenuiviridae* | Severe fever with thrombocytopenia syndrome virus | SFTSV | [51, 297, 359-433] |
|  |  | Hubei diptera virus 3 | HbDV-3 | [434] |
|  |  | Hubei diptera virus 4 | HbDV-4 | [434] |
|  |  | Hubei diptera virus 5 | HbDV-5 | [434] |
|  |  | Hubei lepidoptera virus 1 | HbLV-1 | [434] |
|  |  | Guertu virus | GTV | [297, 435] |
|  |  | Bole tick virus 1 | BlTV-1 | [317] |
|  |  | Changping tick virus 1 | CpTV-1 | [317] |
|  |  | Dabieshan tick virus | DBSH | [317, 436, 437] |
|  |  | Huangpi tick virus 1 | HpTV-1 | [317] |
|  |  | Huangshi humpbacked fly virus | HsHFV | [317] |
|  |  | Lihan tick virus | LITV | [317] |
|  |  | Qingnian mosquito virus | QnMV | [317] |
|  |  | Wuhan fly virus 1 | WhFV-1 | [317] |
|  |  | Wuhan horsefly virus | WhHV | [317] |
|  |  | Wuhan insect virus 1 | WhIV-1 | [317] |
|  |  | Wuhan louse fly virus 2 | WhLFV-2 | [317] |
|  |  | Wuhan millipede virus 1 | WhMV-1-mil | [317] |
|  |  | Wuhan spider virus | WhSV | [317] |
|  |  | Wutai mosquito virus | WtMV | [317] |
|  |  | Wenzhou shrimp virus 1 | WzSV-1 | [317] |
|  |  | Xinzhou mosquito virus | XzMV | [317] |
|  |  | Yichang insect virus | YcIV | [317] |
|  |  | Yongjia tick virus | YONV | [317] |
|  |  | Rift Valley fever virus | RVFV | [438] |
|  |  | Wuxiang virus | WUXV | [439-442] |
|  | *Wupedeviridae* | Wuhan millipede virus 2 | WhMV-2-mil | [317] |
|  | Uncharacterized | Jiangxia mosquito virus 1 | JxMV-1 | [317] |
|  |  | Jiangxia mosquito virus 2 | JxMV-2 | [317] |
|  |  | Shuangao bedbug virus 1 | SgBV-1 | [317] |
|  |  | Shuangao mosquito virus | SgMV | [317] |
|  |  | Shayang spider virus 2 | SySV-2 | [317] |
|  |  | Wuhan insect virus 3 | WhIV-3 | [317] |
|  |  | Wenzhou shrimp virus 2 | WzSV-2 | [317] |
| Africa and the Arabian Peninsula | *Phenuiviridae* | Rift Valley fever virus | RVFV | [443-560] |

**Table S2: Search terminology used for the systematic review.**

| Search | English (Pubmed, GIDEON and GenBank) [Title/Abstract] | Chinese (CHKI and WanFang) [Title/Abstract] |
| --- | --- | --- |
| #1 (Order) | *Bunyavirales* | 布尼亚病毒目 |
| #2 (Family) | *Bunyaviridae* OR *Arenaviridae* OR *Hantaviridae* OR *Phenuiviridae* OR *Nairoviridae* OR *Peribunyaviridae* OR *Phasmaviridae* OR *Wupedeviridae* | 布尼亚病毒科 or 沙粒病毒科 or 砂粒病毒科 or 汉坦病毒科 or 内罗病毒科 or 泛布尼亚病毒科 or 白纤病毒科 |
| #3 (Genus) | *Orthohantavirus* OR *Mammantavirinae* OR *Bandavirus* OR *Orthonairovirus* OR *Phlebovirus* OR *Loanvirus* OR *Thottimvirus* OR *Mobatvirus* OR *Orthobunyavirus* OR *Uukuvirus* OR *Mammarenavirus* OR MARV OR *Wenrivirus* OR *Sawastrivirus* OR *Orthophasmavirus* OR *Shangavirus* OR *Wuhivirus* OR *Goukovirus* OR *Horwuvirus* OR *Phasivirus* OR *Wumivirus* OR *Striwavirus* OR *Shaspivirus* OR *Hudovirus* OR *Beidivirus* OR *Hudivirus* | 汉坦病毒属 or 正内罗病毒属 or 白蛉病毒属 or 正布尼亚病毒属 or 哺乳动物沙粒病毒属 or 哺乳动物砂粒病毒属 |
| #4 (viruses and  Diseases) | Hantaan virus OR Seoul virus OR Hemorrhagic fever with renal syndrome OR Hemorrhagic fever OR Hantavirus OR Dabie bandavirus OR severe fever with thrombocytopenia syndrome virus OR severe fever with thrombocytopenia syndrome OR Crimean-Congo hemorrhagic fever virus OR Crimean-Congo hemorrhagic fever OR Xinjiang hemorrhagic fever virus OR Xinjiang hemorrhagic fever OR Rift Valley fever virus OR Rift Valley fever OR Lianghe virus OR Huangpi virus OR Longquan virus OR Yakeshi virus OR Cao Bang virus OR Xinyi virus OR Kenkeme virus OR Khabarovsk virus OR Fusong virus OR Yuanjiang virus OR Imjin virus OR Amur virus OR Luxi virus OR Nairobi sheep disease virus OR Laibin virus OR Xuan son virus OR Ebinur Lake virus OR Abbey lake virus OR Qian Hu Shan virus OR Fugong virus OR Manzanilla virus OR Cat Que virus OR Songling virus OR Tamdy virus OR Dabieshan virus OR Dabieshan tick virus OR Tacheng tick virus 1 OR Snowshoe hare virus OR Tahyna virus OR Batai virus OR Puumala virus OR Oya virus OR Akabane virus OR Lymphocytic choriomeningitis virus OR Wenzhou mammarenavirus OR Wuxiang virus OR Guertu bandavirus OR Alxa virus OR Lijiang virus OR Ryukyu virus OR Huangpi tick virus 1 OR Wenzhou shrimp virus 1 OR Sanxia water strider Virus 2 OR Lihan tick virus OR Yongjia tick virus OR Wuhan mosquito virus 1 OR Wuhan mosquito virus 2 OR Shuangao insect virus 1 OR Wuhan insect virus 2 OR Wuchang cockroach virus 1 OR Yichang insect virus OR Wuhan horsefly virus OR Wutai mosquito virus OR Wuhan millipede virus 2 OR Wuhan fly virus 1 OR Sanxia water strider virus 1 OR Shayang spider virus 1 OR Wenzhou tick virus OR Tacheng tick virus 2 OR Huangpi tick virus 2 OR Shuangao Insect Virus 2 OR Bole tick virus 1 OR Changping tick virus 1 OR Huangshi humpbacked fly virus OR Jiangxia mosquito virus 1 OR Jiangxia mosquito virus 2 OR Qingnian mosquito virus OR Shayang spider virus 2 OR Shuangao bedbug virus 1 OR Shuangao mosquito virus OR Wenzhou shrimp virus 2 OR Wuhan insect virus 1 OR Wuhan insect virus 3 OR Wuhan louse fly virus 1 OR Wuhan louse fly virus 2 OR Wuhan millipede virus 1 OR Wuhan spider virus OR Xinzhou mosquito virus OR Xinzhou spider virus OR Hubei lepidoptera virus 1 OR Hubei diptera virus 3 OR Hubei diptera virus 4 OR Hubei diptera virus 5 | 汉滩病毒 or 汉城病毒 or 汉坦病毒 or 发热伴血小板减少综合征病毒 or 新布尼亚病毒or 克里米亚刚果出血热病毒 or 新疆出血热病毒 or 梁河病毒 or 黄陂病毒 or 龙泉病毒 or 牙克石病毒 or 信义病毒 or 抚松病毒 or 沅江病毒 or 泸西病毒 or 内罗毕绵羊病 or 内罗毕绵羊病病毒 or 来宾病毒 or 艾比湖病毒 or 千湖山病毒 or 福贡病毒 or 松岭病毒 or 大别山病毒 or 大别山蜱虫病毒 or 塔城病毒 or 雪鞋野兔病毒 or 塔希纳病毒 or 巴泰病毒 or 普马拉病毒or阿卡斑病毒 or 赤羽病 or淋巴细胞脉络丛脑膜炎病毒 or 温州病毒 or 温州沙粒病毒 or 温州砂粒病毒 |
| #5 | China OR The mainland of China OR Chinese mainland OR Hongkong OR Taiwan OR Macao | Select 医药、卫生+生物科学 |
| #6 | #1 OR #2 OR #3 OR #4) AND #5 | (#1 OR #2 OR #3 OR #4) AND #5 |

**Table S3: Information of** **explanatory covariates sources in this study.**

| Covariate | Source | Website | Note | Data period |
| --- | --- | --- | --- | --- |
| Meteorological data | WorldClim | <https://www.worldclim.org/> | Including monthly average temperature and relative humidity which 19 ecoclimatic covariates (Bio01‒19) were created based on; a resolution of 2.5 min of arc. | 1960–2018 |
|  | European Centre for Medium-Range Weather Forecasts (ECMWF） | https://cds.climate.copernicus.eu/cdsapp#!/home | Including monthly 2m temperature and total precipitation with a resolution of 6 min of arc. | 2000-2020 |
| Volumetric soil water layer 1 | ECMWF | https://cds.climate.copernicus.eu/cdsapp#!/home | Volume of water in soil layer 1 (0-7cm) by monthly, with a resolution of 6 min of arc. | 2000-2020 |
| Surface runoff | ECMWF | https://cds.climate.copernicus.eu/cdsapp#!/home | The total amount of water by monthly which drains away over the surface, with a resolution of 6 min of arc. | 2000-2020 |
| Global planar river system data | Resource and Environment Science and Data Center (RESDC) | <http://www.resdc.cn/Default.aspx> | A raster digital map with global planar river system data which the minimum distance to nearest bodies of water were created based on; a resolution of 10km. | - |
| Elevation | EarthEnv-DEM90 digital elevation model Dataset | http://www.earthenv.org/ | A raster digital map with a resolution of 1km. | 2010 |
| Land cover | European Space Agency (ESA) | https://maps.elie.ucl.ac.be/CCI/viewer/index.php | A raster digital map with a resolution of 0.3km. | 1992–2019 |
| Normalized Difference Vegetation Index (NDVI) | NASA Earth Observations (NEO) | https://neo.sci.gsfc.nasa.gov/ | On the basis of monthly data, the annual vegetation index dataset was generated by the maximal synthesis method; a resolution of 6 min of arc. | 2000–2020 |
| Livestock density | Food and Agriculture Organization of the United Nations (FAO) | <http://www.fao.org/livestock-systems/en/> | Including the density of bufflo, cattle, goat and sheep with a resolution of 5 min of arc. | 2010 |
| Mammalian richness | Socioeconomic Data and Applications Center (SEDAC) | https://sedac.ciesin.columbia.edu/ | Provide information on the number of mammal species present at a 1km spatial resolution. | 2013 |
| Population density | LandScan | https://www.satpalda.com/product/landscan/ | A raster digital map with a resolution of 1km. | 2000–2019 |
| Predicted probability of *H. asiaticum* presence | Literature | - | point data and county data, 236 records added | 1985-2021^*^ |

^*^Indicates the publication time of the literature.

**Table S4: Description of 42 potential explanatory covariates used in the modelling efforts.**

| Covariate | Description | Covariate type | CCHFV model  included | RVFV model included | *H. asiaticum* model  included |
| --- | --- | --- | --- | --- | --- |
| Bio1 | Annual mean temperature (℃) | Dynamic, annual | √ |  | √ |
| Bio2 | Mean diurnal range (Mean of monthly (max temp-min temp)) (℃) | Dynamic, annual | √ |  | √ |
| Bio3 | Isothermality (Bio02/Bio07) (*100) | Dynamic, annual | √ |  | √ |
| Bio4 | Temperature seasonality (standard deviation*100) | Dynamic, annual | √ |  | √ |
| Bio5 | Max temperature of warmest month (℃) | Dynamic, annual | √ |  | √ |
| Bio6 | Min temperature of coldest month (℃) | Dynamic, annual | √ |  | √ |
| Bio7 | Annual range of temperature (Bio05-Bio06) (℃) | Dynamic, annual | √ |  | √ |
| Bio8 | Mean temperature of wettest quarter (℃) | Dynamic, annual | √ |  | √ |
| Bio9 | Mean temperature of driest quarter (℃) | Dynamic, annual | √ |  | √ |
| Bio10 | Mean temperature of warmest quarter (℃) | Dynamic, annual | √ |  | √ |
| Bio11 | Mean temperature of coldest quarter (℃) | Dynamic, annual | √ |  | √ |
| Bio12 | Annual precipitation (mm) | Dynamic, annual | √ |  | √ |
| Bio13 | Precipitation of wettest month (mm) | Dynamic, annual | √ |  | √ |
| Bio14 | Precipitation of driest month (mm) | Dynamic, annual | √ |  | √ |
| Bio15 | Precipitation seasonality (Coefficient of variation) | Dynamic, annual | √ |  | √ |
| Bio16 | Precipitation of wettest quarter (mm) | Dynamic, annual | √ |  | √ |
| Bio17 | Precipitation of driest quarter (mm) | Dynamic, annual | √ |  | √ |
| Bio18 | Precipitation of warmest quarter (mm) | Dynamic, annual | √ |  | √ |
| Bio19 | Precipitation of coldest quarter (mm) | Dynamic, annual | √ |  | √ |
| Mean temperature | Temperature of air at 2m above the surface of land, sea or in-land waters (K) | Dynamic, monthly |  | √^*^ |  |
| Mean precipitation | the depth of precipitation (the sum of large-scale precipitation and convective precipitation) would have if it were spread evenly over the grid box (m) | Dynamic, monthly |  | √^*^ |  |
| Soil volumetric water content | Volume of water in soil layer 1 (0 - 7 cm) (m3*m-3) | Dynamic, monthly |  | √^*^ |  |
| Surface runoff | The depth of surface runoff would have if it were spread evenly over the grid box (m) | Dynamic, monthly |  | √^*^ |  |
| Minimum distance to nearest bodies of water | The minimum distance to nearest bodies of water (m) | Static |  | √ |  |
| Elevation | Average altitude (m) | Static | √ | √ | √ |
| Agriculture | Percentage coverage of agriculture (%) | Dynamic, annual | √ | √ | √ |
| Forest | Percentage coverage of forest (%) | Dynamic, annual | √ | √ | √ |
| Grassland | Percentage coverage of grassland (%) | Dynamic, annual | √ | √ | √ |
| Shrubland | Percentage coverage of shrubland (%) | Dynamic, annual | √ | √ | √ |
| Sparse vegetation | Percentage coverage of sparse vegetation (%) | Dynamic, annual | √ | √ | √ |
| Wetland | Percentage coverage of wetland (%) | Dynamic, annual | √ | √ | √ |
| Bare areas | Percentage coverage of bare areas (%) | Dynamic, annual | √ | √ | √ |
| Urban areas | Percentage coverage of urban areas (%) | Dynamic, annual | √ | √ | √ |
| NDVI | Normalized Difference | Dynamic, monthly | √ | √ | √ |
| livestock density | Density of livestock (the sum of buffalo, cattle, goat and sheep) (number per km²) | Static |  | √ |  |
| Buffalo density | Density of buffalo (number per km²) | Static | √ |  | √ |
| Cattle density | Density of cattle (number per km²) | Static | √ |  | √ |
| Goat density | Density of goat (number per km²) | Static | √ |  | √ |
| Sheep density | Density of sheep (number per km²) | Static | √ |  | √ |
| Mammalian richness | The number of mammal species (number per km^2^) | Static | √ | √ | √ |
| Population density | Density of human population (person per km^2^) | Dynamic, annual | √ | √ | √ |
| *H. asiaticum* | predicted probability of *H. asiaticum* presence | Static | √ |  |  |

^*^ Indicates included the value of covariates in a previous month. For example, we included the mean precipitation in January as a covariate in the February model.

**Table S5: Clinical characteristics of** **human cases infections with different viruses (species) belonging to the order *Bunyavirales* in China.**

| **Clinical manifestation** | *Nairoviridae* | | | | |  | *Arenaviridae* |  | *Peribunyaviridae* |  | *Phenuiviridae* |
| --- | --- | --- | --- | --- | --- | --- | --- | --- | --- | --- | --- |
|  | CCHFV | BJNV | SGLV | TcTV−1 | TcTV−2 |  | LCMV |  | TAHV |  | RVFV |
| **Overall cases** | 331 | 68 | 43 | 1 | 1 |  | 41 |  | 13 |  | 1 |
| **Manifestations*** | 142 | 68 | 43 | 1 | 1 |  | 40 |  | 13 |  | 1 |
| **Influenza-like illness (ILI)** |  |  |  |  |  |  |  |  |  |  |  |
| Fever | 114 (80.9) ^†^ | 68 (100.0) | 32 (74.4) | 1 | 1 |  | 20–21 (50.0–52.5) |  | 8 (61.5) |  | 1 |
| Headache | 94–95 (66.7–67.4) | 67 (98.5) | 33 (76.7) | 1 | 1 |  | 27–28 (67.5–70.0) |  | 7 (53.8) |  | 1 |
| Fatigue | 52–69 (36.9–48.9) | 36 (52.9) | 22 (51.2) | 1 | 1 |  | 3–16 (7.5–40.0) |  | 6 (46.2) |  | 1 |
| Chills | 55–72 (39.0–51.1) | 12 (17.6) | 4 (9.3) | 1 | 1 |  | 5–17 (12.5–42.5) |  | 0–2 (0.0–15.4) |  | 1 |
| Dizziness | 5–28 (3.5–19.9) | 0–2 (0.0–2.9) | 21 (48.8) | 1 | 0–1 |  | 2–15 (5.0–37.5) |  | 0–2 (0.0–15.4) |  | 0–1 |
| Cough | 1–25 (0.7–17.7) | 15 (22.1) | 4 (9.3) | 0–1 | 0–1 |  | 2–15 (5.0–37.5) |  | 2 (15.4) |  | 0–1 |
| Chest tightness | 0–25 (0.0–17.7) | 12 (17.6) | 6 (14.0) | 0–1 | 0–1 |  | 0–15 (0.0–37.5) |  | 0–2 (0.0–15.4) |  | 0–1 |
| **Gastrointestinal manifestations** |  |  |  |  |  |  |  |  |  |  |  |
| Anorexia | 37–56 (26.2–39.7) | 24 (35.3) | 13 (30.2) | 1 | 1 |  | 0–15 (0.0–37.5) |  | 6 (69.2) |  | 0–1 |
| Vomiting | 28–46 (19.9–32.6) | 3 (4.4) | 7 (16.3) | 0–1 | 1 |  | 26–27 (65.0–67.5) |  | 4 (30.8) |  | 0–1 |
| Nausea | 33–51 (23.4–36.2) | 2 (2.9) | 11 (25.6) | 0–1 | 1 |  | 9–20 (22.5–50.0) |  | 4 (30.8) |  | 0–1 |
| Abdominal pain | 32–50 (22.7–35.5) | 3 (4.4) | 1 (2.3) | 0–1 | 0–1 |  | 0–15 (0.0–37.5) |  | 0–2 (0.0–15.4) |  | 0–1 |
| Abdominal distention | 13–33 (9.2–23.4) | 0–2 (0.0–2.9) | 0–1 (0.0–2.3) | 0–1 | 0–1 |  | 0–15 (0.0–37.5) |  | 0–2 (0.0–15.4) |  | 0–1 |
| Diarrhea | 18–35 (12.8–24.8) | 3 (4.4) | 2 (4.7) | 0–1 | 0–1 |  | 0–15 (0.0–37.5) |  | 0–2 (0.0–15.4) |  | 0–1 |
| Hematochezia | 23–38 (16.3–27.0) | 0–2 (0.0–2.9) | 0–1 (0.0–2.3) | 0–1 | 0–1 |  | 0–15 (0.0–37.5) |  | 0–2 (0.0–15.4) |  | 0–1 |
| Hematemesis | 7–28 (5.0–19.9) | 0–2 (0.0–2.9) | 0–1 (0.0–2.3) | 0–1 | 0–1 |  | 0–15 (0.0–37.5) |  | 0–2 (0.0–15.4) |  | 0–1 |
| **Neurologic manifestations** |  |  |  |  |  |  |  |  |  |  |  |
| Mental confusion | 14–33 (9.9–23.4) | 42 (61.8) | 1 (2.3) | 0–1 | 0–1 |  | 17–23 (42.5–57.5) |  | 2 (15.4) |  | 0–1 |
| Depression | 1–25 (0.7–17.7) | 42 (61.8) | 23 (53.5) | 0–1 | 0–1 |  | 4–18 (10.0–45.0) |  | 0–2 (0.0–15.4) |  | 0–1 |
| Malaise | 4–27 (2.8–19.1) | 0–2 (0.0–2.9) | 2 (4.7) | 0–1 | 0–1 |  | 2–15 (5.0–37.5) |  | 3 (23.1) |  | 0–1 |
| **Urinary manifestations** |  |  |  |  |  |  |  |  |  |  |  |
| Hematuria | 9–27 (6.4–19.1) | 0–2 (0.0–2.9) | 0–1 (0.0–2.3) | 0–1 | 0–1 |  | 0–15 (0.0–37.5) |  | 0–2 (0.0–15.4) |  | 0–1 |
| Oliguria | 4–25 (2.8–17.7) | 0–2 (0.0–2.9) | 0–1 (0.0–2.3) | 0–1 | 0–1 |  | 0–15 (0.0–37.5) |  | 0–2 (0.0–15.4) |  | 0–1 |
| **Respiratory manifestations** |  |  |  |  |  |  |  |  |  |  |  |
| Hemoptysis | 14–31 (9.9–22.0) | 0–2 (0.0–2.9) | 0–1 (0.0–2.3) | 0–1 | 0–1 |  | 0–15 (0.0–37.5) |  | 0–2 (0.0–15.4) |  | 0–1 |
| **Other manifestations** |  |  |  |  |  |  |  |  |  |  |  |
| Pharyngeal hyperemia | 4–26 (2.8–18.4) | 0–2 (0.0–2.9) | 0–1 (0.0–2.3) | 0–1 | 0–1 |  | 3–16 (7.5–40.0) |  | 7 (58.3) |  | 0–1 |
| Epistaxis | 56–71 (39.7–50.4) | 0–2 (0.0–2.9) | 0–1 (0.0–2.3) | 0–1 | 0–1 |  | 0–15 (0.0–37.5) |  | 0–2 (0.0–15.4) |  | 0–1 |
| Myalgia | 38–56 (27.0–39.7) | 15 (22.1) | 14 (32.6) | 1 | 0–1 |  | 8–15 (20.0–37.5) |  | 4 (30.8) |  | 0–1 |
| Arthralgia | 24–42 (17.0–29.8) | 15 (22.1) | 14 (32.6) | 1 | 0–1 |  | 0–15 (0.0–37.5) |  | 5 (38.5) |  | 1 |
| Rash or petechiae | 30–35 (21.3–24.8) | 22 (32.4) | 13 (30.2) | 1 | 0–1 |  | 0–15 (0.0–37.5) |  | 0–2 (0.0–15.4) |  | 0–1 |
| Conjunctiva hyperemia | 6–28 (4.3–19.9) | 0–2 (0.0–2.9) | 0–1 (0.0–2.3) | 0–1 | 0–1 |  | 0–15 (0.0–37.5) |  | 0–2 (0.0–15.4) |  | 0–1 |

*Cases with incomplete characteristic information were excluded. ^†^Data are presented as n (%), where n is the number of human cases reported the clinical manifestation, and % is the rate. For avoiding overestimation of the clinical manifestations resulted from those publications that some clinical manifestations were not reported or missing, we given an interval of positive number for those manifestations rather than zero: the minimum was zero and the maximum was the same to the minimum positive number of all reported clinical manifestations in this publication. The ratio is not calculated if N is no more than 10 due to the deficient representativeness. The clinical manifestations of human cases with HFRS (caused by hantavirus, including HTNV and SEOV) and SFTS (caused by SFTSV) which had been widely reported in China were not included.

**Table S6: The model-predicted areas and population sizes at risk by monthly for RVFV occurrence in China.**

| Month | Model-predicted area**^#^** (10,000 km2) | Model-predicted population size^†^ (million) |
| --- | --- | --- |
| January | 23.24 | 123.579638 |
| February | 13.77 | 81.489966 |
| March | 31.68 | 135.151215 |
| April | 59.70 | 218.600003 |
| May | 219.25 | 902.197468 |
| June | 377.32 | 1112.778864 |
| July | 465.67 | 1067.421558 |
| August | 434.50 | 1010.673551 |
| September | 443.01 | 1008.806447 |
| October | 382.48 | 1094.019073 |
| November | 189.09 | 827.608401 |
| December | 68.04 | 251.017227 |

**^#^** Model-predicted area = number of pixels in risk * 100km^2^

^†^ Model-predicted population size was calculated by adding up the average population of each pixel at risk.

**Fig. S1: The flow diagram of data collection for Rift Valley fever virus.**


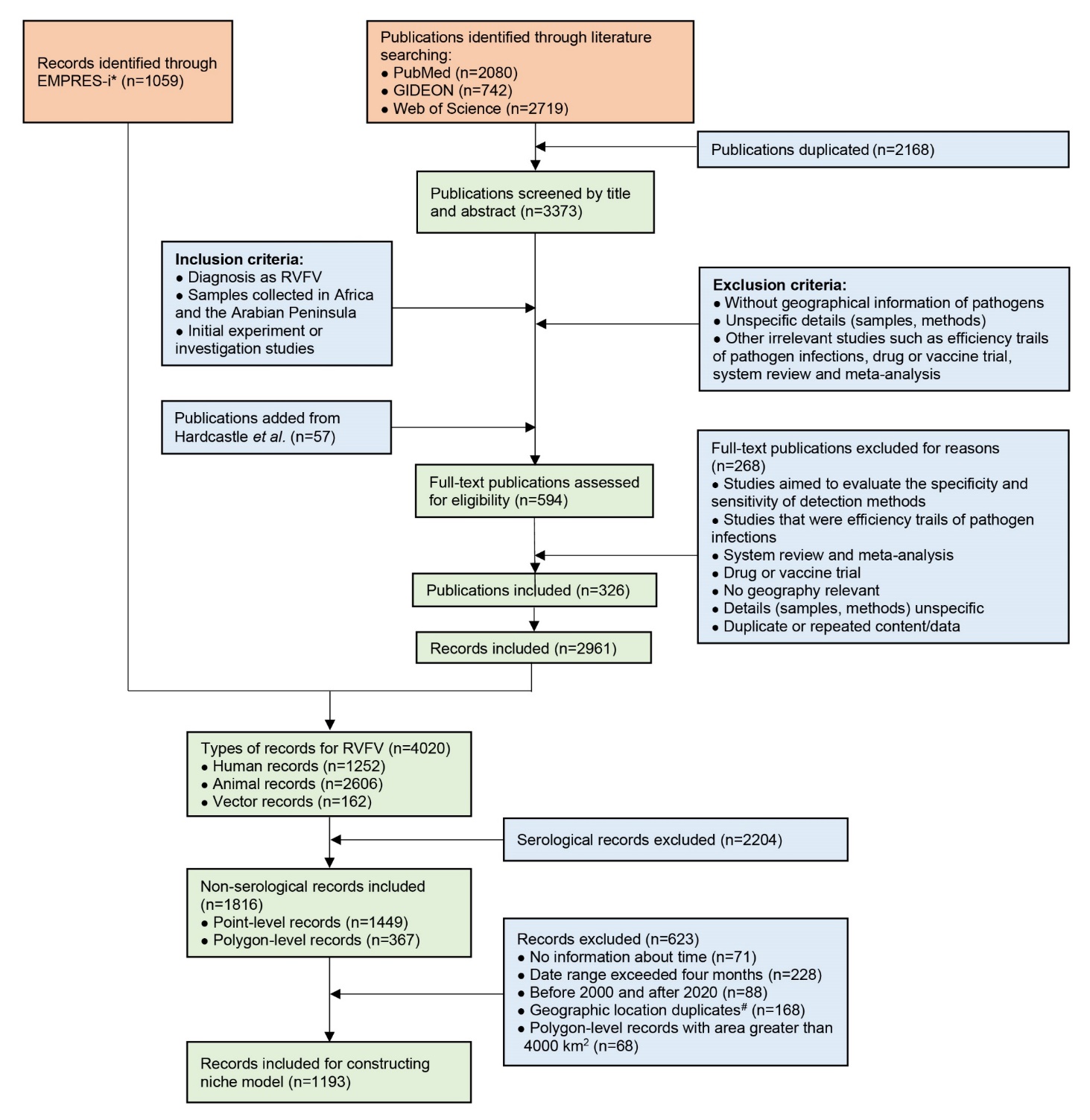


* Global Animal Disease Information System (EMPRES-i). ^#^ Geographic location duplicates included between point-level records and point-level records, between polygon-level records and polygon-level records, between polygon-level records and point-level records (the record about polygon corresponding to the point-level record was removed).

**Fig. S2: Recorded and predicted risk distribution of *H. asiaticum* presence in China.** (A) Each occurrence record of *H. asiaticum* was geo-referenced and linked to the digital China map. (B) Predicted risk distribution of *H. asiaticum* after averaged 100 bootstrapping BRT models.


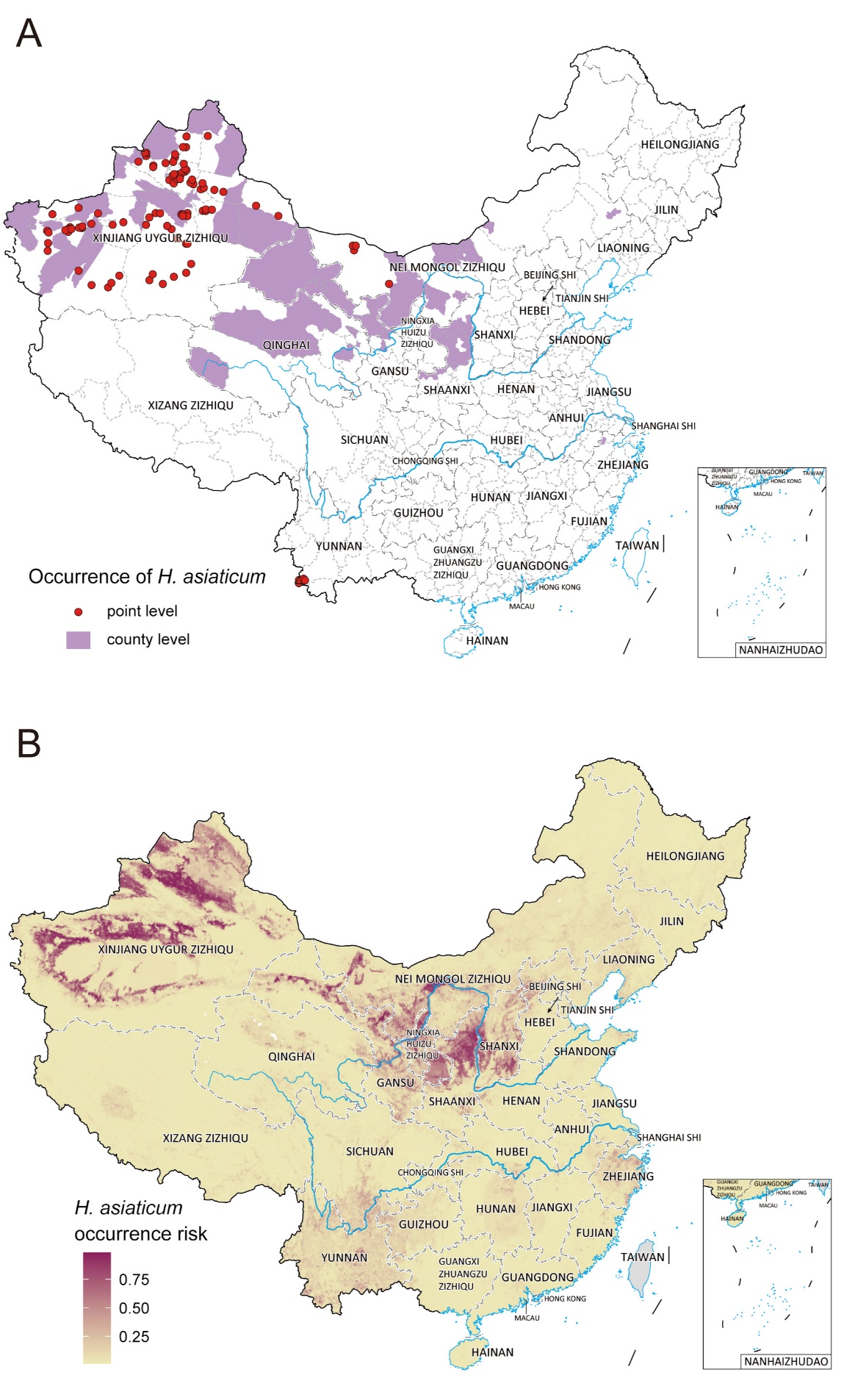


**Fig. S3: Correlation matrix for 36 covariates in CCHFV model.** Heatmap representing the correlation between features using Spearman correlation coefficient. Red and blue colors in the plot represent the positive and negative monotonic relationship respectively. The absolute value of the correlation coefficient was represented by the size of the circle, and the exact value shown in the lower triangle. The variables marked red represent those excluded by multicollinearity screening.


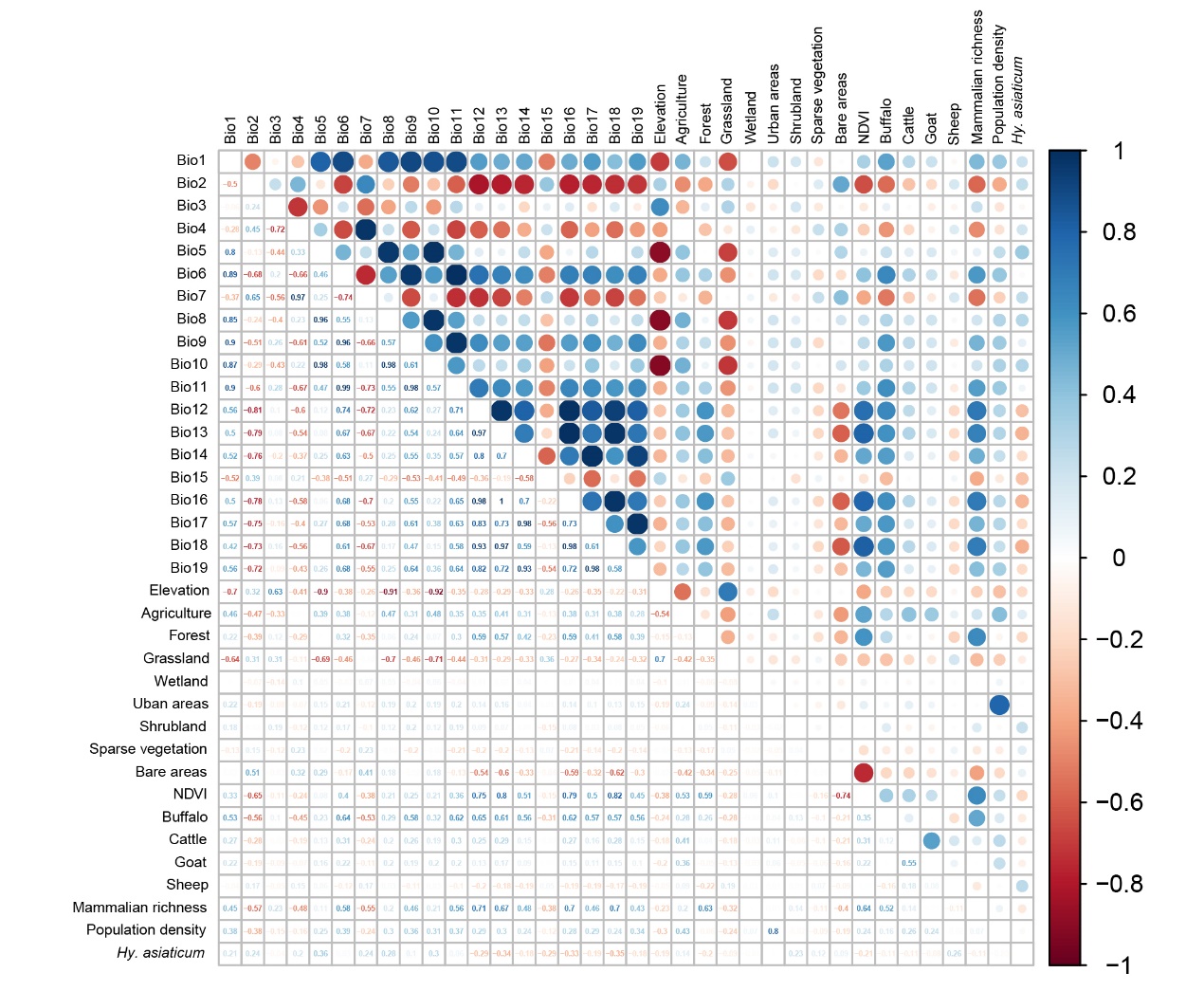


**Fig. S4: Correlation matrix for 18 covariates in RVFV model.** Heatmap representing the correlation between features using Spearman correlation coefficient. Red and blue colors in the plot represent the positive and negative monotonic relationship respectively. The absolute value of the correlation coefficient was represented by the size of the circle, and the exact value shown in the lower triangle. The variables marked red represent those excluded by multicollinearity screening.


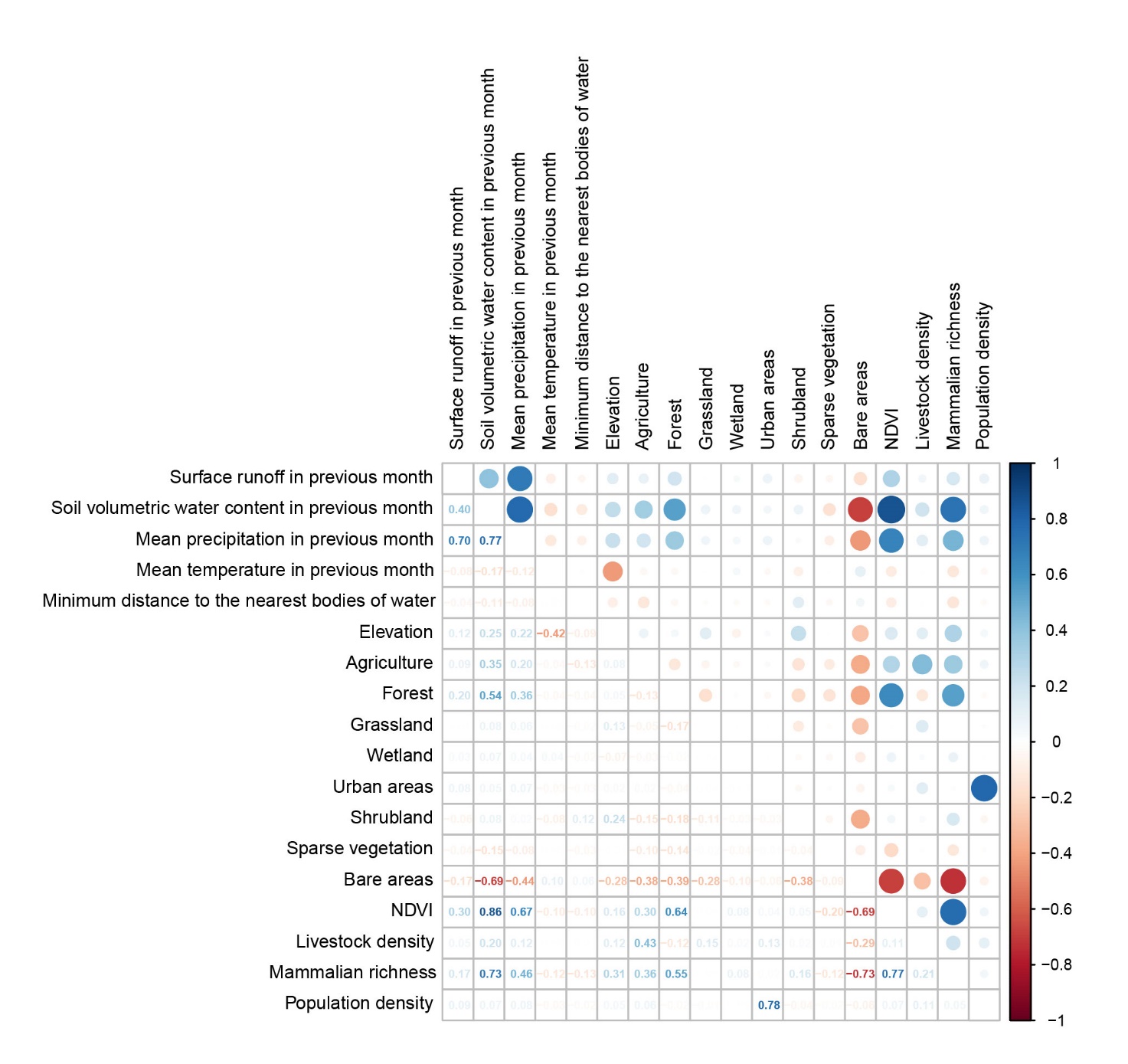


**Fig. S5: Distribution of the first identification site of 89 *Bunyavirales* viruses in China.** The location where the virus was firstly identified is determined at the city level as referred to the included literatures. ^†^The virus was not included in the latest International Committee on Taxonomy of Viruses Report. The full name of each virus was shown in Table 1.


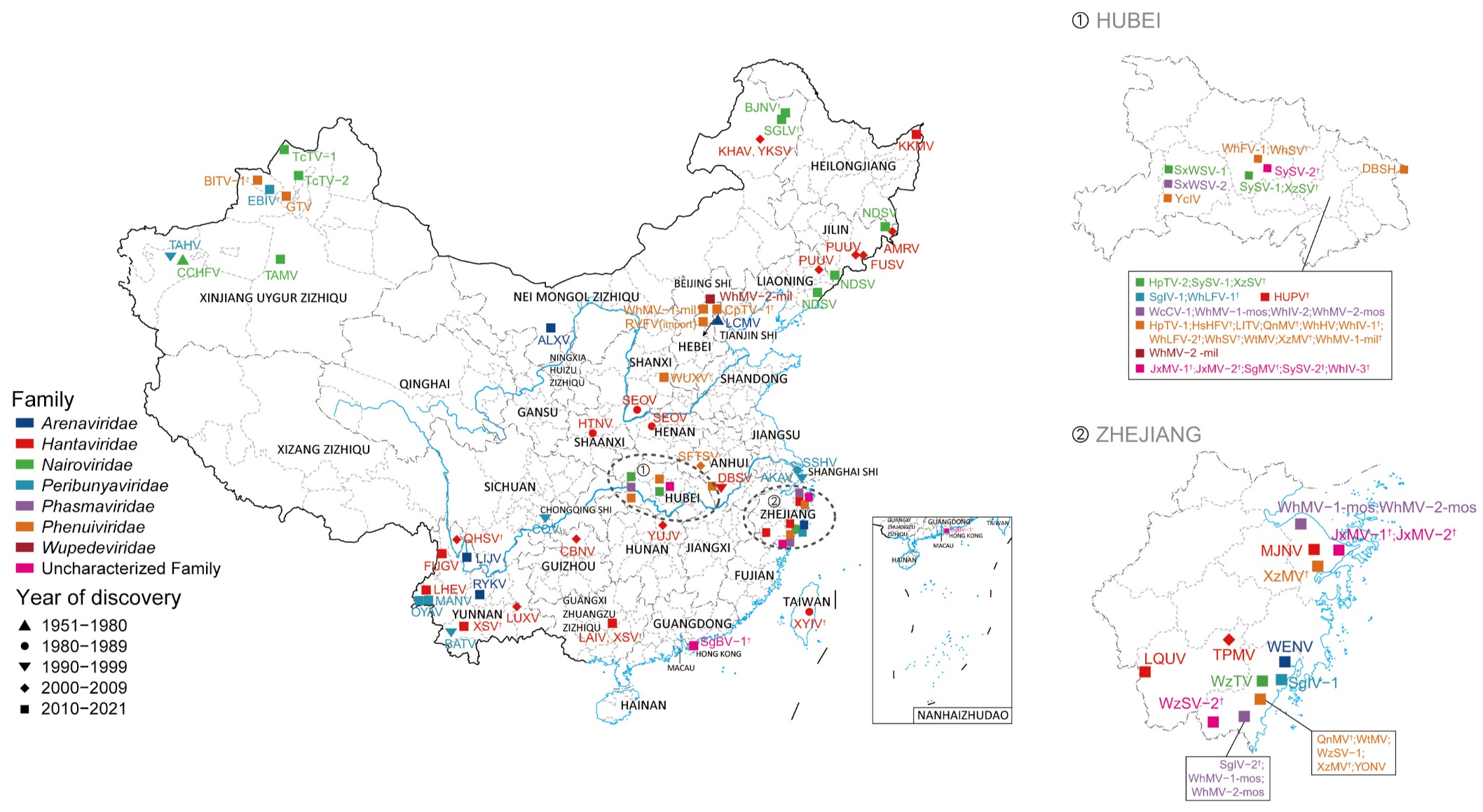


**Fig. S6: Geographical distributions of *Bunyavirales* viruses detected in animals. (A)** The distribution of *Bunyavirales* virus species in animals determined by molecular methods or virus isolation. **(B)** The distribution of animal species that carrying *Bunyavirales* viruses, determined by molecular methods or virus isolation. **(C)** The distribution of *Bunyavirales* virus species in animals determined by serological methods. **(D)** The distribution of animal species that carrying *Bunyavirales* viruses, determined by serological methods. Locations with positive records were positioned at the center of either provinces or cities depending on data availability, among which the data at the province level were circled with dashed lines. The full name of each virus was shown in Table 1.


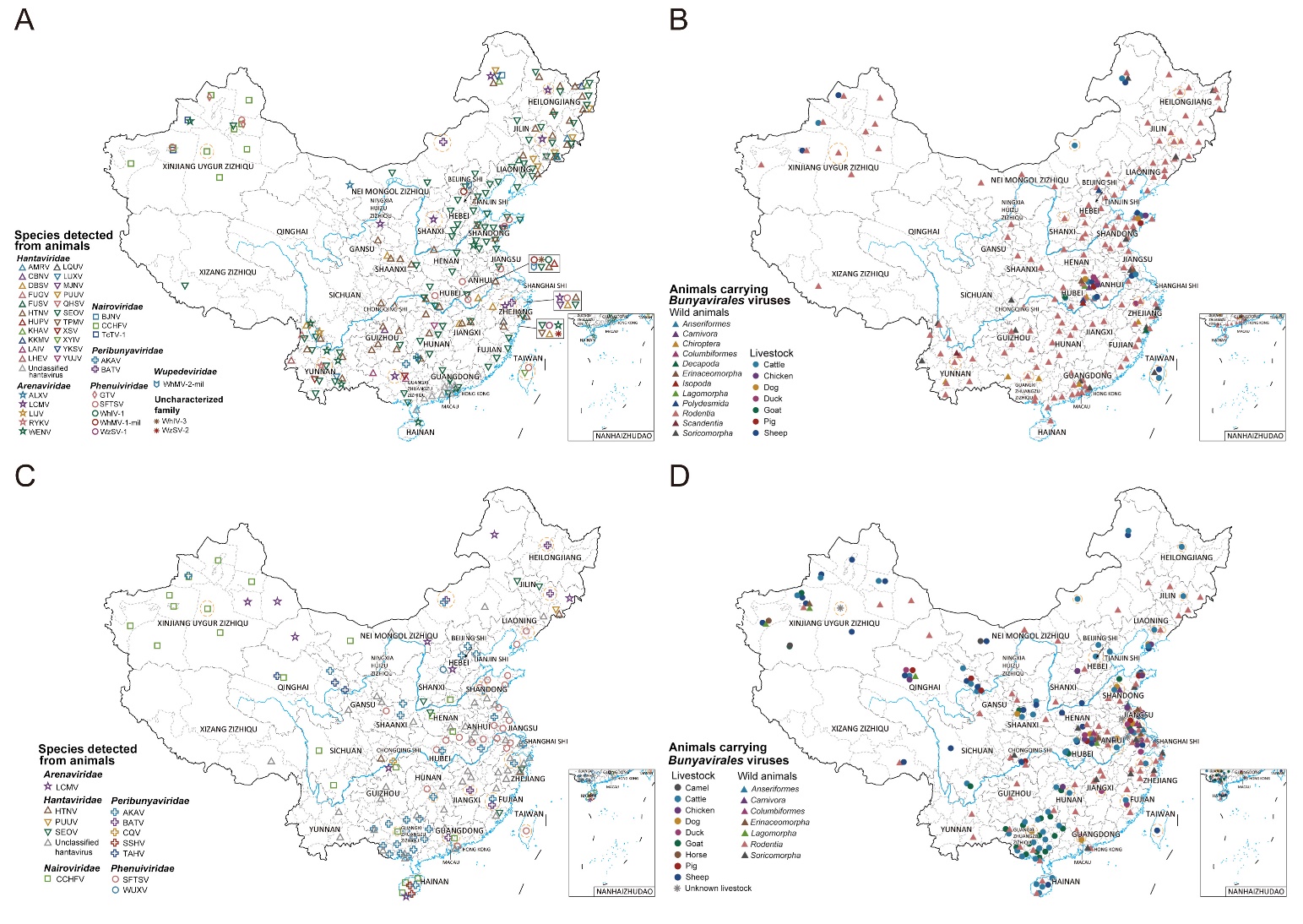


**Fig. S7: The relationship matrix of *Bunyavirales* virus species and involved animals.** The viruses that could infect humans with any detection method, including molecular, serological and virus isolation method, were marked by purple fonts. ^†^This represented infection of human-infected viruses in humans detected only by serological methods. *Hantavirus referred to unclassified hantavirus. The dark green square represented infection of human-infected viruses in animals detected by molecular methods or virus isolation, while light green indicated viruses that had not been found in humans detected by molecular methods or virus isolation, and grey represented viruses detected by serological tests whether the viruses infected with humans or not. The full name of each virus was shown in Table 1.


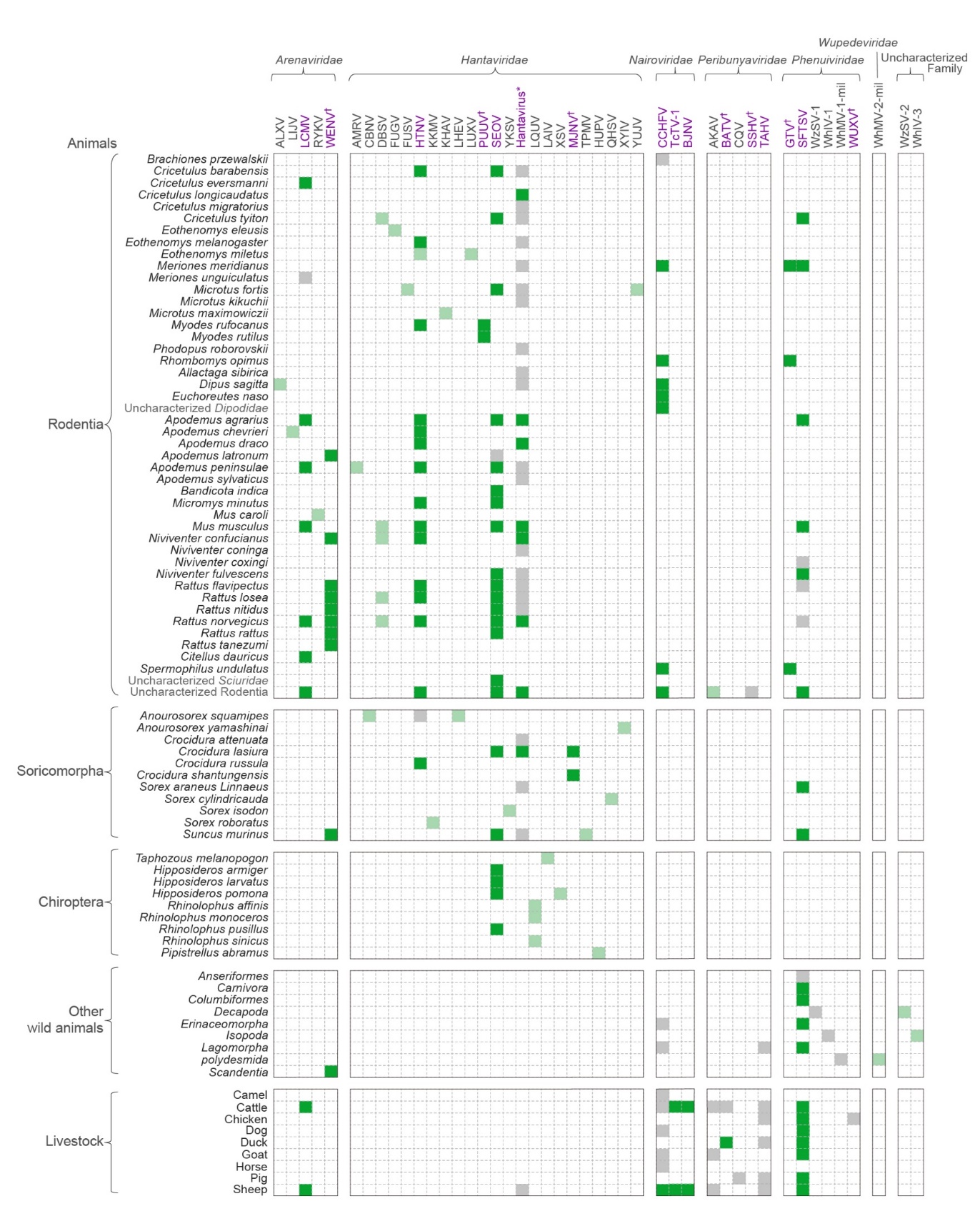


**Fig. S8: ROC curves and AUC values of CCHFV model.** Performance evaluation of the BRT model after averaged 100 bootstrapping models. AUC: area under the receiver operating characteristics curve.


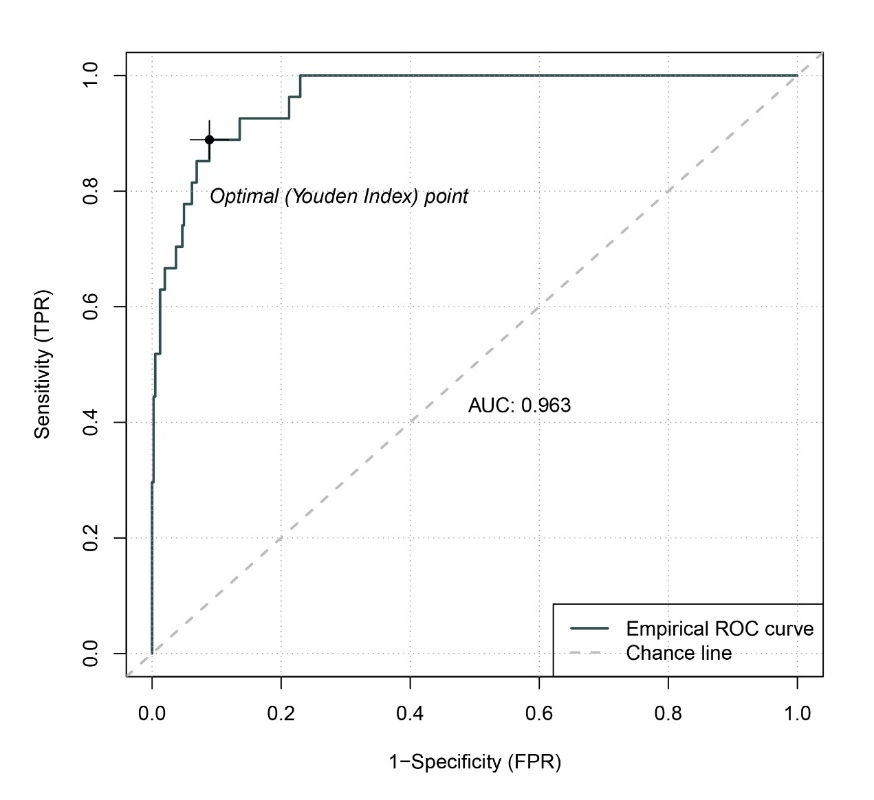


**Fig. S9: The marginal effect of explanatory covariates for CCHFV occurrence risk in the model.** The red curves and gray bands show the average and range, respectively, of predicted occurrence risk from 100 BRT model using bootstrap method. Frequency distributions of the predictors are shown by the histograms in blue. The percentage values in parentheses show the relative contributions averaged over all 100 BRT models.


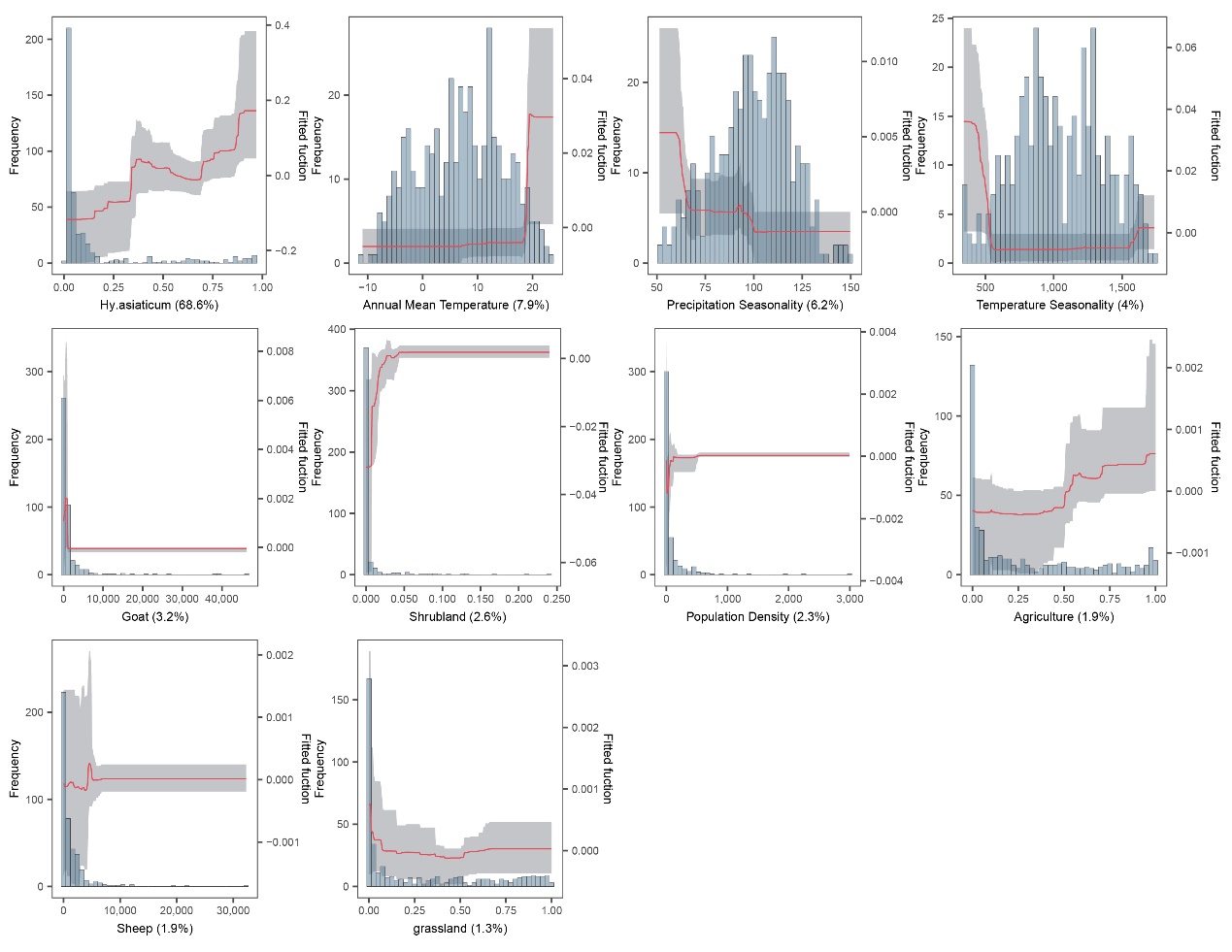


**Fig. S10: Recorded distribution of RVFV occurrence** **in Africa and the Arabian Peninsula.** **(A)** All records of RVFV occurrence from the EMPRES-i database and literatures. **(B)** Records of RVFV occurrence used for modelling. An occurrence record was defined as one or more confirmed infection(s) with RVFV at a unique location (the same administrative district or 10 km×10 km pixel for points) regardless of the type of hosts, detection methods or time points with positive detection. The RVFV occurrence records detected by serological methods were marked by green, otherwise it turned into red or blue if the records were detected by molecular methods and virus isolation. The coordinates of centroid points were both displayed for administrative district or 10 km×10 km pixel level records.


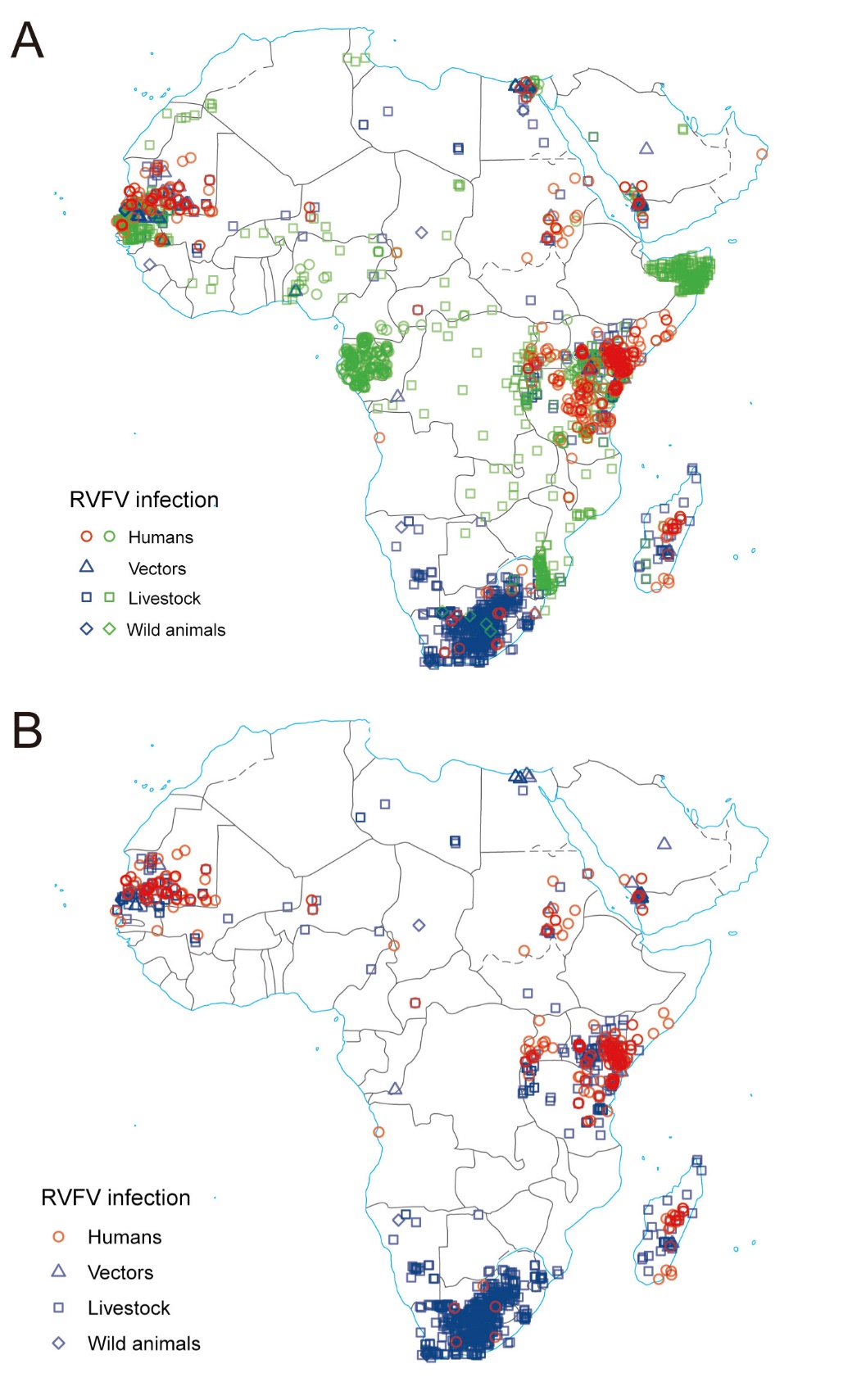


**Fig. S11: ROC curves and AUC values of RVFV model.** Performance evaluation of the BRT model after averaged 100 bootstrapping models. AUC: area under the receiver operating characteristics curve.


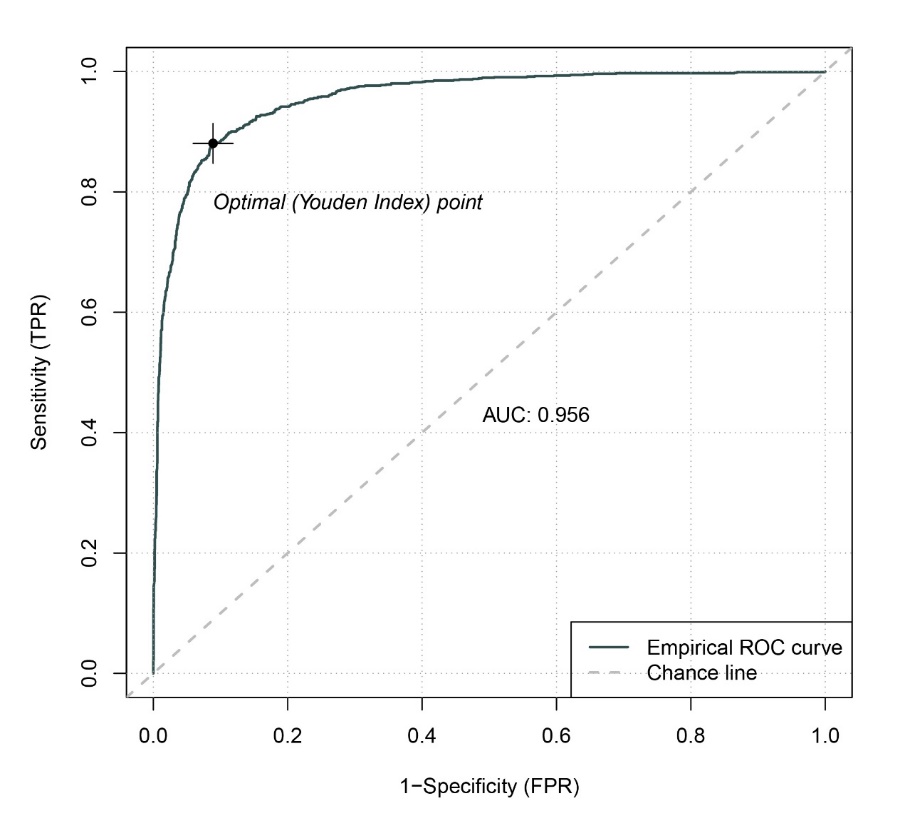


**Fig. S12: The marginal effect of explanatory covariates for RVFV occurrence risk in the model.** The red curves and gray bands show the average and range, respectively, of predicted occurrence risk from 100 BRT model using bootstrap method. Frequency distributions of the predictors are shown by the histograms in blue. The percentage values in parentheses show the relative contributions averaged over all 100 BRT models.

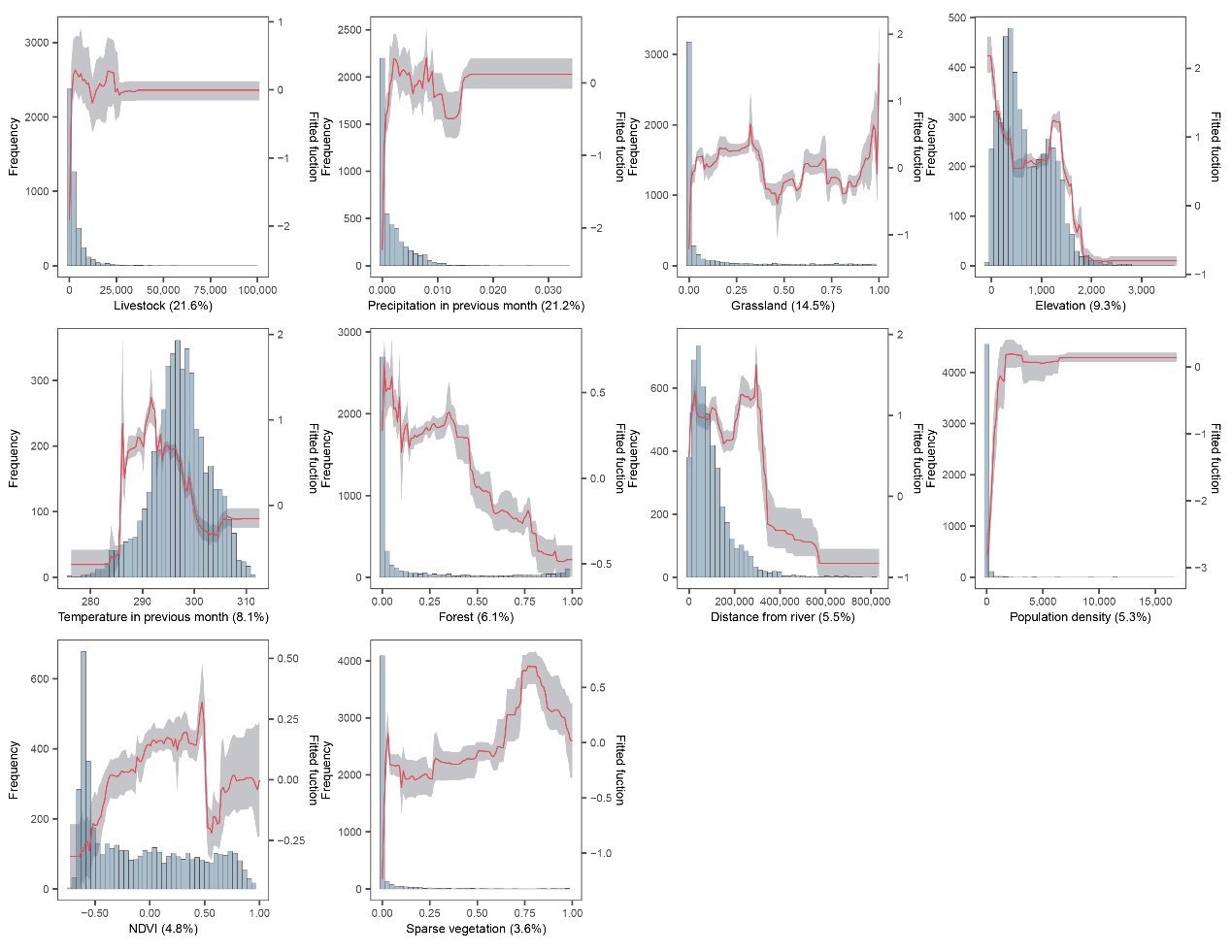


**Fig. S13: Mean environmental suitability and monthly imported risk for RVFV from January to June in China.** Each monthly map represented the average environmental suitability predictions in that month across the years 2000–2020. Areas shown in purple are more suitable than those shown in green. The circle represents the city where the airport in China was located. The monthly imported risk at the city level was classified into four categories: very low (imported risk index ≤10^-3^); low (imported risk index between 10^-3^ and 10^-2^); medium (imported risk index between 10^-2^ and 10^-1^); high (imported risk index >10^-1^).


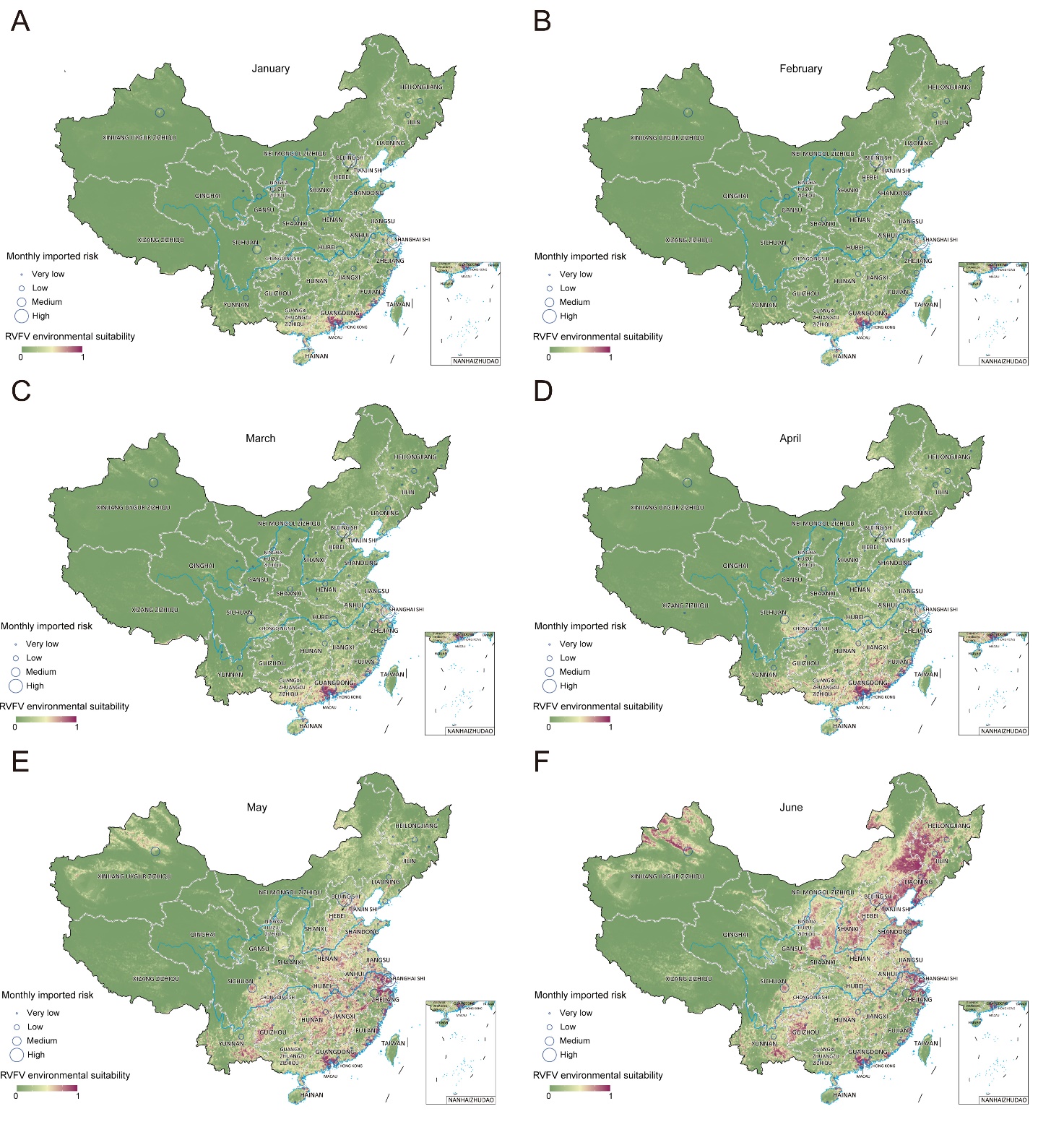


**Fig. S14: Mean environmental suitability and monthly imported risk for RVFV from July to December in China.** Each monthly map represented the average environmental suitability predictions in that month across the years 2000–2020. Areas shown in purple are more suitable than those shown in green. The circle represents the city where the airport in China was located. The monthly imported risk at the city level was classified into four categories: very low (imported risk index ≤10^-3^); low (imported risk index between 10^-3^ and 10^-2^); medium (imported risk index between 10^-2^ and 10^-1^); high (imported risk index >10^-1^).


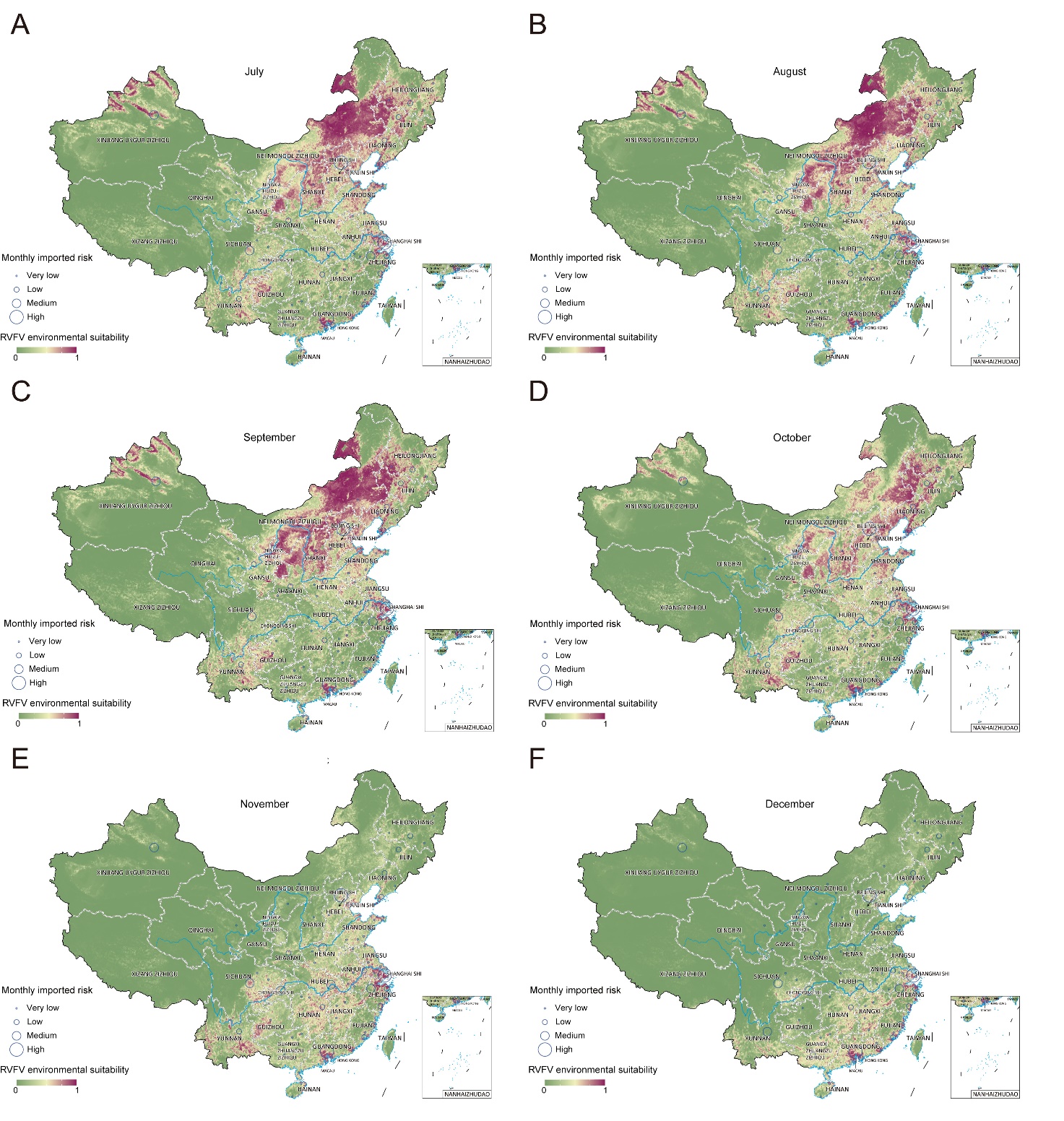


**Supplementary References**

1. Fang LQ, Liu K, Li XL, Liang S, Yang Y, Yao HW, et al. Emerging tick-borne infections in mainland China: an increasing public health threat. Lancet Infect Dis. 2015; 15(12): 1467-79.

2. Hardcastle AN, Osborne JCP, Ramshaw RE, Hulland EN, Morgan JD, Miller-Petrie MK, et al. Informing Rift Valley fever preparedness by mapping seasonally varying environmental suitability. Int J Infect Dis. 2020; 99: 362-72.

3. Messina JP, Pigott DM, Golding N, Duda KA, Brownstein JS, Weiss DJ, et al. The global distribution of Crimean-Congo hemorrhagic fever. Trans R Soc Trop Med Hyg. 2015; 109(8): 503-13.

4. Okely M, Anan R, Gad-Allah S, Samy AM. Mapping the environmental suitability of etiological agent and tick vectors of Crimean-Congo hemorrhagic fever. Acta Trop. 2020; 203: 105319.

5. Gale P, Stephenson B, Brouwer A, Martinez M, de la Torre A, Bosch J, et al. Impact of climate change on risk of incursion of Crimean-Congo haemorrhagic fever virus in livestock in Europe through migratory birds. J Appl Microbiol. 2012; 112(2): 246-57.

6. Estrada-Peña A, Ruiz-Fons F, Acevedo P, Gortazar C, de la Fuente J. Factors driving the circulation and possible expansion of Crimean-Congo haemorrhagic fever virus in the western Palearctic. J Appl Microbiol. 2013; 114(1): 278-86.

7. Ak Ç, Ergönül Ö, Gönen M. A prospective prediction tool for understanding Crimean-Congo haemorrhagic fever dynamics in Turkey. Clin Microbiol Infect. 2020; 26(1): 123.e1-.e7.

8. Métras R, Jewell C, Porphyre T, Thompson PN, Pfeiffer DU, Collins LM, et al. Risk factors associated with Rift Valley fever epidemics in South Africa in 2008-11. Sci Rep. 2015; 5: 9492.

9. Redding DW, Tiedt S, Lo Iacono G, Bett B, Jones KE. Spatial, seasonal and climatic predictive models of Rift Valley fever disease across Africa. Philos Trans R Soc Lond B Biol Sci. 2017; 372(1725).

10. Clements AC, Pfeiffer DU, Martin V. Application of knowledge-driven spatial modelling approaches and uncertainty management to a study of Rift Valley fever in Africa. Int J Health Geogr. 2006; 5: 57.

11. Williams R, Malherbe J, Weepener H, Majiwa P, Swanepoel R. Anomalous high rainfall and soil saturation as combined risk indicator of Rift Valley fever outbreaks, South Africa, 2008-2011. Emerg Infect Dis. 2016; 22(12): 2054-62.

12. Zhao GP, Wang YX, Fan ZW, Ji Y, Liu MJ, Zhang WH, et al. Mapping ticks and tick-borne pathogens in China. Nat Commun. 2021; 12(1): 1075.

13. Linthicum KJ, Britch SC, Anyamba A. Rift Valley Fever: An Emerging Mosquito-Borne Disease. Annu Rev Entomol. 2016; 61: 395-415.

14. Rueda LM, Patel KJ, Axtell RC, Stinner RE. Temperature-dependent development and survival rates of *Culex quinquefasciatus* and *Aedes aegypti* (*Diptera*: *Culicidae*). J Med Entomol. 1990; 27(5): 892-8.

15. Zhuang ZC. A case of lymphocytic choriomeningitis treated by huoxueshui method. Journal of Traditional Chinese Medicine. 2002; (06): 470.

16. Zhang XD, Qiu FX, Wang HC. Lymphocytic choriomeningitis: report of a chronic patient. Chin J Intern Med. 1954; 02(3): 169-71.

17. Zhang L, Li S, Huang SJ, Wang ZD, Wei F, Feng XM, et al. Isolation and genomic characterization of lymphocytic choriomeningitis virus in ticks from northeastern China. Transbound Emerg Dis. 2018; 65(6): 1733-9.

18. Zhang L. Epidemiology of Lymphocytic choriomeningitis virus in cattle and sheep in Hulun Buir [Master Dissertation]. Changchun(Jilin): Jilin Agricultural University, **2019**.

19. Xiao XM. Death from lymphocytic choriomeningitis: a case report. Chinese Pediatric Emergency Medicine. 1998; (03): 2.

20. Wu G, Jin YL, Sun S. Lymphocytic choriomeningitis complicated with SAH1: a case report. Journal of Apoplexy and Nervous Diseases. 2002; (05): 1.

21. Morita C, Tsuchiya K, Ueno H, Muramatsu Y, Kojimahara A, Suzuki H, et al. Seroepidemiological survey of lymphocytic choriomeningitis virus in wild house mice in China with particular reference to their subspecies. Microbiol Immunol. 1996; 40(4): 313-5.

22. Li HY. Four cases of lymphocytic choriomeningitis treated with aminosalicylic acid. Railway Medical Journal. 1981; (04): 250.

23. Ji HW, Liu ZY, Li XH, Wang D, Zhang L, Hou ZJ, et al. Isolation, identification and epidemiology investigation of lymphocytic choriomeningitis virus (LCMV) in parts of China. Chinese Journal of Veterinary Parasitology. 10.

24. Huang SF, Meng XC, Qu YF. Lymphocytic choriomeningitis: 2 cases. Journal of Harbin Medical University. 2001; (03): 208.

25. Huang DY, Wang SJ, Zhong HL, Zhang XD, Gao CJ, Weng XZ, et al. Lymphocytic choriomeningitis: a report of seven cases. Chin J Intern Med. 1956; 04(8): 614-20.

26. Hu Q, Zou CY, Ma SJ, Tong SM. Establishment of a real-time quantitative PCR technique to detect lymphocytic choriomeningitis virus carried by rodents. Journal of Parasitic Biology. 2014; 9(10): 877-9.

27. Hu Q, Qiu JL, Ma SJ, Ma Y, Li J. RT-PCR detection and N gene sequence analysis of Lymphocytic choriomeningitis virus in rodents. Chin J Vector Biol Control. 2013; 24(06): 483-5.

28. Hao R, Zhang T, Yang H. Lymphocytic choriomeningitis: a case report. Internal Medicine of China. 2010; 5(03): 322.

29. Dong ML, Dong XY, Wang Y, Lu J, Li SL, Wang J. Detection of virus in wild and domesticated Mongolian Gerbil by ELISA. Chin J Zoono. 2013; 29(04): 366-8+74.

30. Cheng WB, Wu K. Lymphocytic choriomeningitis complicated with subarachnoid hemorrhage: a case report. Journal of Forensic Medicine. 2010; 26(04): 307.

31. Chen YY. Jiedu decoction treated 22 cases of lymphocytic choriomeningitis. Chinese Community Doctors. 2004; (08): 41.

32. Wu JY, Guo C, Xia Y, Bao HM, Zhu YS, Guo ZM, et al. Genomic characterization of Wenzhou mammarenavirus detected in wild rodents in Guangzhou City, China. One Health. 2021; 13: 100273.

33. Zhang Y. Investigation of the emerging infectious virus in important animal reservoir in Hainan [Master Dissertation]. Haikou(Sanya): Hainan Medical University, **2018**.

34. Wang J, Yang X, Liu H, Wang L, Zhou J, Han X, et al. Prevalence of W¨¥nzh¨­u virus in small mammals in Yunnan province, China. PLoS Negl Trop Dis. 2019; 13(2): e0007049.

35. Wang B, Cai CL, Li B, Zhang W, Zhu Y, Chen WH, et al. Detection and characterization of three zoonotic viruses in wild rodents and shrews from Shenzhen city, China. Virol Sin. 2017; 32(4): 290-7.

36. Tan ZZ. Virome profiling of rodents in Xinjiang, China: isolation and characterization of a new Wenzhou mammarenavirus variant [Master Dissertation]. Changchun(Jilin): Jilin Agricultural University, **2018**.

37. Liu MM. The study of viral metagenomics in rodents from Shandong [Master Dissertation]. Qingdao(Shandong): Qingdao University, **2018**.

38. Li K, Lin XD, Wang W, Shi M, Guo WP, Zhang XH, et al. Isolation and characterization of a novel arenavirus harbored by rodents and shrews in Zhejiang province, China. Virology. 2015; 476: 37-42.

39. Jiang MY. Study on the infection of a species of Arenavirus in hosts and patients with fever in Yunnan province, China [Master Dissertation]. Dali(Yunnan): Dali University, **2019**.

40. Guo L, Liu S, Song J, Han L, Zhang H, Wu C, et al. Seroprevalence of Wenzhou virus in China. Biosaf Health. 2020; 2(3): 152-6.

41. Wu Z, Du J, Lu L, Yang L, Dong J, Sun L, et al. Detection of hantaviruses and arenaviruzses in three-toed jerboas from the Inner Mongolia autonomous region, China. Emerg Microbes Infect. 2018; 7(1): 35.

42. Zou Y, Hu J, Wang ZX, Wang DM, Yu C, Zhou JZ, et al. Genetic characterization of hantaviruses isolated from Guizhou, China: evidence for spillover and reassortment in nature. J Med Virol. 2008; 80(6): 1033-41.

43. Zou Y, Hu J, Wang ZX, Wang DM, Li MH, Ren GD, et al. Molecular diversity and phylogeny of hantaan virus in Guizhou, China: evidence for Guizhou as a radiation center of the present hantaan virus. J Gen Virol. 2008; 89(Pt 8): 1987-97.

44. Zhu YQ, Jia N, Tian QW, Wu GC, Dong DD, Zhang H, et al. Study on antibody detections and genotypes of the hantavirus in Qingdao district, China. Chinese Journal of Laboratory Diagnosis. 2017; 21(09): 1504-6.

45. Zhou JH, Zhang HL, Wang JL, Yang WH, Zhang YZ. A study on rodents infected with hantavirus and their genotypes in four counties, Yunnan province. China Preventive Medicine. 2009; 10(11): 984-6.

46. Zhang YZ, Xiao QY, Li MH, Zou Y, Lv W, Dai DF, et al. An epidemiologic investigation of hantaviruses carried by rodent hosts in Hunan province. Chin J Epidemiol. 2007; 28(1): 65-9.

47. Zhang YS, Ma Y, Liu P, Zhang Y, Zhang CM, Tang K, et al. Retrospective analysis of long-term immunity protection in a case of hantaan virus infection after 44 years. Infectious Disease Information. 2020; 33(04): 324-6.

48. Zhang YH, Lu XH, Chen HX. Isolation and identification of hantaviruses from reservoir animals captured in nidi of HFRS in Ningxia. Chin J Microbiol Immunol. 1999; (06): 4.

49. Zhang P, Chen YW, Dong YB, He T, Qi YL, Li Q. Monitoring and analysis of hemorrhagic fever with renal syndrome in host animals in Shangluo city, Shaanxi province from 2013 to 2015. Journal of Medical Pest Control. 2020; 36(01): 63-5+8.

50. Zhang J, Xu G, Gong T, Xiong Y, Shi Y, Li JX, et al. Analysis on genetic characteristics of hantavirus from rodents in Jiangxi province. Chin J Vector Biol Control. 2015; 26(5): 475-9.

51. Zhang BH, Zhang H, Zhang X, Tan QQ, Zhou HQ, Sun JF, et al. Spectrum of pathogens causing syndromes of fever and hemorrhage in Guangdong province, China. Chin J Publ Heal. 2018; 34(06): 864-7.

52. Yu JW, Gao J, Sun LJ, Li SC. Retrospective analysis of epidemiology and clinical characteristics of hemorrhagic fever with renal syndrome in Harbin area during 10 years. Chin J Infect. 2009; (03): 172-5.

53. Yu JW, Gao J, Sun LJ, Li SC. Clinical characteristics and serotype analysis of 87 cases of hemorrhagic fever with renal syndrome in Harbin area in 2005. The Seventh National Conference on Hemorrhagic Fever with Renal Syndrome. Hangzhou, China, **2006**:1.

54. Yao ZH, Dong GM, Zhang JK, Yu YX, Liu XC, Liu WX, et al. Analysis on the genetics of hantavirus S85-46 isolated from Sichuan province. Chin J Epidemiol. 2001; (06): 3.

55. Yao ZH, Dong GM, Yu YX, Zhang JK, Liu XC, Zhang LL, et al. A new subtype of hantavirus SN7 isolated from Niviventer Confucianus in Sichuan province, China. Chin J Infect. 2002; (02): 4.

56. Yao PP, Chen G, Xu F, Yang ZN, Chen C, Sun YS, et al. Genotype and evolution of hantavirus in Tiantai of Zhejiang province, 2011-2018. Chin J Epidemiol. 2019; (10): 1285-90.

57. Yao LS. Study of composition of rodents and their ectoparasites and pathogens infection in the adjacent port area of Changbai mountain of China and Democratic People's Republic of Korea (DPRK) [Ph.D Dissertation]. Beijing: Academy of Military Medical Sciences, **2012**.

58. Yao K, Jiang JF, Zhang WY, Zhao QM, Jiang XN, Zuo SQ, et al. Molecular epidemiological investigation on hantavirus carried by rodents in some areas of northeastern China. Journal of Parasitic Biology. 2007; 2(1): 1-3,10.

59. Yang ZQ, Yu SY, Chen Q, Nie J, Liu YX, Zhang JL, et al. Antigen typing of the HFRSV strains and serotyping of patients and main host animals from Shandong area. Practical Journal of Medicine & Pharmacy. 2005; 22(4): 343-6.

60. Yang DJ. Studies on molecular epidemiology of hantavirus in Tianjin and GP multi-epitope antigen gene construction and expression [Ph.D Dissertation]. Tianjin: Tianjin Medical University, **2009**.

61. Yan YC, Liu XL, Yang ZB, Li ZL. Propagation and characterization of the etiologic agent of epidemic hemorrhagic fever in cultured A-549 cells. Acta Academiae Medicinae Sinicae. 1982; 4(2): 67-72. (in Chinese).

62. Yan QL, Yang PF, Shao LJ, Liu YX, Pu Y, Zhang XL, et al. The analysis of hantavirus S gene in Apodemus Agrarius in Changbai area. Chin J Virol. 2013; 29(04): 382-5.

63. Xu YJ. Epidemiological characteristics of epidemic hemorrhagic fever in Baishan city from 2008 to 2017. Strait J Prev Med. 2019; 25(1): 35-6.

64. Xu F, Zhu HP, Yao PP, Hu D, Zhang Y, Xie RH, et al. Isolation and identification of hantavirus carried by rodents in Zhejiang province, China from 2008 to 2011. Chin J Vector Biol Control. 2013; 24(04): 285-8.

65. Xu F, Yao PP, Zhu HP, Xie RH. Isolation and identification of 3 strains of hemorrhagic fever with renal syndrome virus. Chin J Vector Biol Control. 2008; 19(06): 562-3.

66. Wu R, Yu PB, Li J, Dong QL, Chen HL, Liu JF, et al. Complete genome sequencing and analysis of hantavirus isolated from Xi 'an. Shanxi Medical Journal. 2013; 42(12): 1581-2+5.

67. Wang ZQ, You XD, Wang YL, Wang XJ, Kang DM, Wang M, et al. Surveillance of hemorrhagic fever with renal syndrome host virus in Shandong province from 1998 to 2002. Preventive Medicine Tribune. 2003; 9(6): 693-4.

68. Wang YL, Cai D, Xu Y, Zhang J, Fu RK, Shi SL. Surveillance on rats and hantavirus detection in Rizhao plain area. Chinese Journal of Frontier health and Quarantine. 2016; 39(06): 417-9+06.

69. Wang CQ, Gao JH, Li M, Guo WP, Lu MQ, Wang W, et al. Co-circulation of hantaan, kenkeme, and khabarovsk hantaviruses in Bolshoy Ussuriysky island, China. Virus Res. 2014; 191: 51-8.

70. Tao KH, Zhu J, Zhang Y, Tang JQ, Wu GH. Study on detection, prolification and location of HFRSV in chigger mite. Chin J Publ Heal. 1999; (04): 3.

71. Tang LY, Yu T, Liu WX, Chen HX. Study on types of hemorragic fever with renal syndron in Chinese epidemic areas (I) Detection of serum types of HFRS in Gaoan and Guangzhou areas. Dis Surveill. 2000; 15(7): 248-9.

72. Tan X, Xiao D, Yan YP. Analysis of epidemic situation of hemorrhagic fever with renal syndrome in Huxian, Xi¡¯ an, China from 1971 to 2010. Chin J Vector Biol Control. 2012; 23(06): 577-80.

73. Sun YL, Dou XF, Liu F, Li X, Li XY. Genotyping and molecular tracing of one patient died from hemorrhagic fever with renal syndrome, 2020. Chinese Journal of Frontier health and Quarantine. 2021; 44(04): 237-40.

74. Sun YF, Hu K, Wu YP. Analysis on results of the hemorrhagic fever with renal syndrome cases of serum diagnosis. Journal of Medical Pest Control. 2016; 32(09): 984-6.

75. Shen B, Pu DF, Wu DL, Xu S, Wu YP, Yang XD, et al. Isolation and identification of hantavirus in Fusong area. Chin J Publ Heal Engineering. 2012; 11(02): 97-9+103.

76. Shen B, Gou WM, Wei LL, Wu DL, Xu S. Detection and analysis of hantavirus nucleic acid in the cases of hemorrhagic fever with renal syndrome serum in Yanbian Korean Autonomous prefecture. Chin J Publ Heal Engineering. 2015; 14(05): 396-8.

77. Qiu JM, Dong ZP, Hang CS, Xie WM, Song G. Hantavirus RNA detection in clinical sera of hemorrhagic fever with renal syndrome using reverse transcription-nested PCR. Chin J Virol. 1997; (02): 7.

78. Qin CM, Liu Y, Yao WQ, Sun YW, Han YH, Zhang JB. Nosetiology and molecular biology analysis of rats infected with HFRS in Liaoning province. Chin J Zoono. 2010; 26(6): 528-31.

79. Qiao G, Pang SS, Zhang HL, Gong Y, Cheng ML, Li ZH. Analysis of part of M gene and genotyping for M segment of hantavirus detected from HFRS patients' sera in Qingdao region during 2000- 2003. Chin J Exp Clin Virol. 2005; 19(1): 22-4.

80. Peng Y, Zou WJ, Li GM. Study on genetic characteristics of hantavirus carried by murids in Xiantao city of Hubei province during 2012. Laboratory Medicine and Clinic. 2015; 12(18): 2720-1.

81. Ma J, Zheng LJ, Tang J, Li SJ, Huo YQ. Molecular characteristics of hantavirus isolated from patients with hemorrhagic fever with renal syndrome in several regions of Henan province (HFRS). Chin J Zoono. 2021; 37(03): 216-20.

82. Ma C, Li MH, Zhang FX, Zhang YZ. Genetic types of hantavirus carried by rodent hosts in Hulunbeier Inner Mongolia. Journal of Shihezi University (Natural Science. 2007; (06): 741-5.

83. Liu T, Xia SG, Mei FS, Hao HB, Nie XP, Huang JG, et al. Surveillance results of host animals of hemorrhagic fever with renal syndrome in Jingzhou, Hubei province, China from 2017 to 2018. Chin J Vector Biol Control. 2020; 31(04): 469-73.

84. Liu SW, Gong T, Xu G, Shi Y, Li JX, Zhang YN, et al. Complete genome sequence analysis of hantaan virus strain AYW89-15 isolated in Jiangxi, China. Chin J Zoono. 2017; 33(12): 1089-93.

85. Liu J. Genetic analysis of hantaviruses and their hosts in Hubei province [Ph.D Dissertation]. Wuhan(Hubei): Wuhan University, **2011**.

86. Liu FQ, Gao LD, Dai DF, Zhang H, Zeng G, Guo SH, et al. Study on hemorrhagic fever with renal syndrome surveillance in Hunan province, 2006. China Preventive Medicine. 2008; 9(6): 496-9.

87. Li JM, Zhang HL, Yang WH, Zhou JH, Wang JL, Zhang YZ, et al. Study on host animals and their genotyping of hantavirus in Yunnan province of China in 2007. Endemic Dis Bull. 2009; 24(06): 1-7+11.

88. Li HB, Fu HL, Hu K, Hu XQ, Deng F, Zhang KJ, et al. Survey on host animals of hemorrhagic fever with renal syndrome in Baoji city of Shaanxi province from 2014 to 2018. Chinese Journal of Hygienic Insecticides & Equipments. 2020; 26(06): 529-32.

89. Huang XG, Yang CH, Guo LL, Hua X. Analysis of surveillance data of hemorrhagic fever with renal syndrome in Zunyi county from 1962 to 2000. Preventive Medicine Tribune. 2002; 8(4): 437-,40.

90. Hu Q, Tong SM, Guo LP. Genotyping and DNA sequenece analysis of hantaviruses at Daxie port. Chinese Journal of Frontier health and Quarantine. 2010; 33(04): 224-8.

91. Hou Y, Li DP, Liu YP, Ju WD, Cheng C, Gao DN, et al. Epidemiologic investigation and analysis on hantavirus carried by rodents at Heilongjiang frontier ports between Sino-Russian border in 2009~2010. Chinese Journal of Frontier health and Quarantine. 2012; 35(3): 186-9.

92. He C, Shao LJ, Huang XG, Yu YQ, Liu Y. Study on genotype of hantavirus at Changchun Longjia international airport. Chin J Vector Biol Control. 2009; 20(03): 246-8.

93. Gu XS, Meng GR, Song ZB, Peng LT, You ZQ, Su Q, et al. A 5-year investigation of hantavirus infection in small mammals and healthy people in epidemic hemorrhagic fever epidemic area. Journal of Preventive Medicine Information. 1994; (04): 3.

94. Gong ZY, Xia JH, Zhao ZY, Fu GM, Weng JQ, Jiang LP. Analysis of surveillance results of hemorrhagic fever with renal syndrome in Zhejiang province in 1995. Practical Preventive Medicine. 1998; (06): 2.

95. Geng YZ, Tian J, Liu Y, Wang B, Sun YW, Li X, et al. Genetic features and distribution of hantaan virus in Liaoning province, China. Chin J Vector Biol Control. 2012; 23(05): 449-51+54.

96. Fu GM, Zhao ZY, Cui QR, Chen YF, Yao ZH, Xu SF, et al. A surveillance report about the hemorrhagic fever with renal syndrome in Tiantai county, 1984-2002. Zhejiang Journal of Preventive Medicine. 2004; (06): 10-1.

97. Dong X, Zhang YZ, Li X, Zhao CZ, Wang B. Genotype of isolated strain of hantavirus in Liaoning area. Chin J Exp Clin Virol. 2005; 19(1): 39-42.

98. Cui BJ, Yi HH, Wu BY, He J, Hu SS, Yang QG, et al. Investigation and analysis on pathogens carried by rodents at ports of Jiangsu from 2014 to 2015. Chinese Journal of Hygienic Insecticides & Equipments. 2016; 22(01): 73-6.

99. Cheng C, Ju WD, Fu WM, Cao SY, Xu N, Wang YM, et al. Epidemiologic investigation hantavirus carried by rodents at Heilongjiang frontier ports. Chin J Zoono. 2015; 31(7): 681-6.

100. Chen YP, Wu YQ, Gao GJ, Chen T, Ma XH, Dong JJ, et al. Analysis of nucleic acid sequence of a virus strain amplified from a HFRS patient in Baoding district. Infectious Disease Information. 1998; (01): 14-5.

101. Chen X. Isolation and sequence analysis of hantavirus from Jilin in northeast of China [Master Dissertation]. Chengdu(Sichuan): Sichuan University, **2005**.

102. Chen SH, Chen LF, Liu YC, Li JH, Hui S. Isolation and gene identification of a hantaan virus strain in Heilongjiang province. Chinese Journal of Natural Medicine. 2008; (03): 224-6.

103. Chen HX, Wang H, Jia KL, Luo CW, Liu GM, Yu T, et al. Study on serotypes of patients with hemorrhagic fever with renal syndrome in China. Chin J Publ Heal. 1995; (05): 4.

104. Cao ZW. Infection and genetic variation of hantavirus in small mammals in some regions of China [Master Dissertation]. Beijing: Academy of Military Medical Sciences;, **2011**.

105. Cao SY. Molecular epidemiology investigation on the rodent-borne hantaviruses isolated from the port cities of Heilongjiang, China, in 2014 [Master Dissertation]. Harbin(Heilongjiang): Northeast Forestry University, **2016**.

106. Bi FY, Tan Y, Mo JJ, Xie ZG, Chen MM. Genotypes of hantavirus in north areas of Guangxi. Journal of Applied Preventive Medicine. 2016; 22(05): 383-5+97.

107. Zuo SQ, Wu XM, Sun PY, Zhang PH, Wang BC, Tang F, et al. Study on the molecular epidemiology of hantaviruses carried by hosts in northern suburb of Beijing. Chin J Epidemiol. 2004; 25(5): 421-4.

108. Zuo SQ. Molecular epidemiology study on hemorrhagic fever with renal syndrome in Beijing [Ph.D Dissertation]. Beijing: Academy of Military Medical Sciences;, **2007**.

109. Zou Y, Zhang HL, Zhang YZ, Mi ZQ, Yang WH, Yuan QH, et al. The epidemiologic investigation and characterization of hantavirus carried by rattus in Yunnan. Chin J Vector Biol Control. 2006; 17(5): 399-403.

110. Zhou JH, Zhang HL, Wang JL, Yang WH, Mi ZQ, Zhang YZ, et al. Survey on host animal and molecular epidemiology of hantavirus in Chuxiong prefecture, Yunnan province. Chin J Epidemiol. 2009; 30(3): 239-42.

111. Zhao S, Chen ZQ, Zhan ZH, Ye TT, Wang XY. Investigation on hantaviruses carried by rodents in Nansha ports. Chin J Vector Biol Control. 2016; 27(6): 591-3.

112. Zhao C, Zhao Y, Li Y, Yuan ZH, Sun KX, Zhang L, et al. Genotype analysis of HFRS pathogen in Jilin area. Journal of Microbiology. 2011; 31(5): 101-4.

113. Zhang XM, Song SX, Di WJ, Wang M, Li XJ, Wang ZQ, et al. Molecular epidemiological investigation of hantavirus isolated from Shandong province. Chin J Vector Biol Control. 2009; 20(5): 457-60.

114. Zhang X, Ma MX, Zhang Z, Tian J, Sun PL, Zhou DY, et al. Molecular epidemiology of hantavirus isolated from rodent hosts in Jinzhou city. Chin J Publ Heal. 2015; 31(01): 39-41.

115. Zhang FX, Zhang YZ, Chen HX, Zhang YM, Bao FB, Wang DW, et al. Genotype of hantavirus carried by the rodents in Hohhot city. Chin J Vector Biol Control. 2007; 18(1): 45-8.

116. Yang PF, Ma C, Gao N, Zhang YZ, Zhu JB. The molecular epidemiology investigation of hantavirus in Bayannaoer region. Journal of Southwest China Normal University(Natural Science Edition). 2008; (03): 109-12.

117. Yan YZ, Yao LS, Hu GW, Dong ZS, Li MH, Zhang YZ. Genotyping of hantavirus of seoul type in Jilin province. Chin J Vector Biol Control. 2006; (04): 324-6.

118. Yan QL, Xing YZ, Li BB, Pang YX, He F, Liu CC, et al. Genetic characteristics of Seoul orthohantavirus in rodents in Huai¡¯an, Jiangsu province. Chinese Journal of Frontier health and Quarantine. 2019; 42(04): 254-8+83.

119. Xiong HP, Li MH, Zhu Y, Dong ZX, Tian JH, Chen HX, et al. Molecular epidemiology of hantavirus carried by rodent hosts in Wuhan, Hubei province. China Tropical Medicine. 2010; 10(6): 658-60.

120. Wu J. Analysis of geographic epidemiology and pathogenic gene sequence of hemorrhagic fever with renal syndrome in ChangChun [Ph.D Dissertation]. Changchun(Jilin): Jilin University, **2015**.

121. Wei YM, Han ZY, Zhang YB, Han X, Cai YN, Xu YG, et al. The isolation, recovery and identification of hantavirus in Hebei province. Chin J Vector Biol Control. 2016; 27(5): 447-9,58.

122. Wang ZY, Song SX, Wang ZQ, Bi ZQ. Molecular epidemiology of hemorrhagic fever with renal syndrome virus isolated from Shandong province. Proceedings of the 7th Epidemiology Academic Exchange Conference in East China. Weihai, China, **2004**:313-8.

123. Wang ZQ, Wang YL, Fu JH, Zhao L, Sun CY, Zhang XQ, et al. Molecular analysis of hantavirus isolated from Shandong province. Chin J Exp Clin Virol. 2003; 17(2): 121-3.

124. Wang YP, Xu Q, Cai HZ, Chen J, Zhang JM, Zheng YP, et al. Molecular epidemiological investigation on hantavirus carried by rodents in Fujian port. Chin J Vector Biol Control. 2012; 23(4): 292-4.

125. Wang YL, Yang YL. Analysis of surveillance results of hemorrhagic fever with renal syndrome in Jinjiang city from 2011 to 2012. Strait J Prev Med. 2013; 19(06): 41-2.

126. Wang Y, Wei YM, Han X, Han ZY, Cai YN, Qi SX, et al. Host rodent and gene characteristics of hantavirus in main endemic areas of hemorrhagic fever with renal syndrome in Hebei province. Chin J Vector Biol Control. 2017; 28(6): 553-6.

127. Wang Y. Molecular and epidemiology studies of hantavirus on nature focus of infection of hemorrhagic fever with renal syndrome [Master Dissertation]. Urumchi(Xinjiang): Xinjiang Agricultural University, **2003**.

128. Wang AN, Dang YQ, Li BQ, Li YJ, Liu Z. Genotyping and sequence analysis of wild animals carrying viruses in Raohe area, China. Chin J Zoono. 2020; 36(03): 206-10.

129. Tan Y, Bi FY, Wei ZL, Yang JY. Host animals of hemorrhagic fever with renal syndrome and hantavirus infections among host animals in Guangxi. Chin J Vector Biol Control. 2010; 21(6): 566-8.

130. Sun Y, Sun BX, Shen B, Wu J. Genotype analysis of hantavirus carried by the rodents in Shuangyang area of Changchun city. Chinese Journal of Hygienic Insecticides & Equipments. 2016; 22(03): 283-7.

131. Sun XF, Zhao L, Zhang ZT, Liu MM, Xue ZF, Wen HL, et al. Detection of Imjin virus and seoul virus in Crocidurine shrews in Shandong province, China. Vector Borne Zoonotic Dis. 2017; 17(6): 425-31.

132. Sun L, Zhang YZ, Li LH, Zhang YP, Zhang AM, Hao ZY, et al. Genetic subtypes and distribution of hantavirus type II in Henan province. Chin J Epidemiol. 2005; (08): 578-82.

133. Sun BX, Sun Y, Shen B, Wu J. Genotype analysis of hantavirus carried by the rodents in Changchun city. Chin J Vector Biol Control. 2015; 26(06): 600-4.

134. Song SX. Sequence and molecular epidemiology analyses of hemorrhagic fever with renal syndrome virus [Master Dissertation]. Jinan(Shandong): Shandong University, **2005**.

135. Ma HB, Sun H, Dong J, Tan H, Lin JC, Yao RD, et al. Epidemiologic investigation on hantaviruses carried by rodents in Zhuhai. Chinese Journal of Frontier health and Quarantine. 2009; 32(04): 243-5+50.

136. Luo YN. Analysis on the surveillance results of hemorrhagic fever with renal syndrome in Cixi city from 1996 to 2011. Chin J Vector Biol Control. 2012; 23(03): 265-7.

137. Liu Y, Jiang LY, Ding P, Wang DH, Xiao XC. Molecular epidemiology analyze of a hemorrhagic fever with renal syndrome case. Journal of Medical Pest Control. 2012; 28(06): 635-6+9.

138. Liu SW, Xu G, Gong T, Shi Y, Li JX, Liu XQ, et al. The detection and genotyping of hantavirus in rats in Nanchang city. Modern Preventive Medicine. 2016; 43(06): 1085-8+96.

139. Liu JJ, Yang F, He JF, Zhang XL, Liang C, Zhang SX, et al. Molecular epidemiology of hantavirus infection in host animals in Shenzhen. Proceedings of the Second Annual Conference of the Chinese Preventive Medicine Association and the Second Annual Conference of the Global Chinese Public Health Association. Xianghe, China, **2006**:1.

140. Liu DP, Wan Q, Qu RW, Liu DH, Sun N, Liu YL, et al. Analysis on genetic characteristic hantavirus carrying by rats, Dalian city 2012. Preventive Medicine Tribune. 2013; 19(12): 893-5+903.

141. Li Y, Shi LL, Liu JN, Yan JH, Nie WZ, Liu SP. S gene sequence analysis of hantavirus carried by the Rattus norvegicus from Qinhuangdao port. Chin J Vector Biol Control. 2017; 28(05): 487-9.

142. Li MH, Chen XP, Yang GQ, Shen TF, Liu B, Guo WP, et al. Analysis of hantavirus carried by Rattus norvegicus in residential areas of Huludao. Chin J Vector Biol Control. 2011; 22(3): 239-42.

143. Li M, Li LM, Zhang LB, Yuan LH, Chen JP. Primary report of Seoul hantavirus detected in bats from Guangdong province. Guangdong Agricultural Sciences. 2014; 41(08): 177-80+84.

144. Hu TS, Hu QL, Li SX, Huang Y, Hu HM, Gao LF, et al. Epidemiological features of hemorrhagic fever with renal syndrome and the host animals in Chuxiong, Yunnan province, China, 2015-2018. Chin J Vector Biol Control. 2020; 31(2): 152-7,63.

145. Hu T, Fan Q, Hu X, Deng B, Chen G, Gu L, et al. Molecular and serological evidence for Seoul virus in rats (Rattus norvegicus) in Zhangmu, Tibet, China. Arch Virol. 2015; 160(5): 1353-7.

146. He X, Qi CL, Wang C, Jiang YZ. Analysis on monitoring results of rodents at Lianyungang seaport from 2014 to 2015. Port Health Control. 2016; 21(5): 41-4.

147. Hang CS, Song G, Qiu XZ, Du YL, Zhao JN, Liao HX. Investigation of the agent causing mild type of hemorrhagic fever. Chin J Epidemiol. 1982; 3(204;): 5. (in Chinese).

148. Guo T. Epidemiological status and host animal survey of hemorrhagic fever with renal syndrome in Yunnan province from 2014 to 2015 [Master Dissertation]. Dali(Yunnan): Dali University, **2016**.

149. Guo G, Xu J, Huang L, Sheng JL, Na. Nucleic acid detection and genotyping analysis of hantavirus among rodents in Xinjiang Uyghur Autonomous region, China. Chin J Vector Biol Control. 2013; 24(02): 144-6.

150. Geng YZ, Tian J, Liu Y, Wang B, Sun YW, Li X, et al. Genetic subtypes and distribution of Seoul virus in Liaoning province. Chin J Publ Heal. 2012; 28(12): 1594-6.

151. Gao YF, Song GR, Tan Z, Jiang L, Cheng XL, Song FL. Analysis on population structure of rodents and hantavirus infection at Liaoning ports from 2008 to 2015. Chinese Journal of Frontier health and Quarantine. 2017; 40(02): 100-3.

152. Gao N. The epidemiologic investigation and characterization of hantavirus in some region of Xinjiang and Inner Mongolia [Master Dissertation]. Shihezi(Xinjiang): Shihezi University, **2008**.

153. Fan ST, Gao XL, Li YG, Ying Y, Guo J, Zhang ZW, et al. Genetics and evolution of viruses carried by animal hosts of hantaviruses in Jilin province, China. Chin J Biol. 2014; 27(4): 467-71+75.

154. Fan FN, Yang PF, Shi NF, Gao N, Chen GH, Li MH, et al. Study on the molecular epidemiology of hantavirus carried by rodent hosts in Cixi, Zhejiang province. Chin J Epidemiol. 2008; (04): 365-8.

155. Fan FN, Xu JY, Fan CP. Surveillance and analysis of hemorrhagic fever with renal syndrome in Cixi city from 1996 to 2005. Chin J Vector Biol Control. 2008; (03): 254-5.

156. Dong ZX, Tan YJ, Xiong HP, Chen HX, Zhang YZ, Zheng JH. The molecular epidemiological investigation of hantavirus in Ouhai district of Wenzhou city. China Tropical Medicine. 2009; 9(4): 608-10.

157. Dong YH, Li Y, Ma H, Wang HF, Xu BL, Huang XY. Pathogenic analysis of rat infected hantavirus in Henan province, 2014-2016. Tianjin Medical Journal. 2017; 45(06): 648-51.

158. Deng YL, Chen WQ, Sun XK, Zhan QL, Luo JR. Surveilance on hantaan virus natural infection of rodents in Qingyuan. Proceedings of the Symposium on Hot Spots in the Prevention and Control of Emerging and Re-emerging Infectious Diseases. Zhuhai, China, **2011**:1.

159. Chen WQ, Luo WL, Huang JY, Li BQ. Survey on hantavirus infection of rat and health people in Qingyuan city. Occupation and Health. 2018; 34(22): 3149-51.

160. Cao YC, Gao R, Wang LX, Li M. Analysis of hantavirus antigen detection in Taonan city, Jilin province in 2013. Chinese Journal of Control of Endemic Disenaces. 2015; 30(04): 272.

161. Bi FY, Tan Y, Wei ZL. Detection of rodent specimen from epidemic hemorrhagic fever in Guangxi. Journal of Applied Preventive Medicine. 2007; 13(4): 199-202.

162. Bao HM, Wei YH, Yuan LH, Chen SY, Yang ZC, Lu JH. The detection and genotyping of hantavirus in small mammals in Guangzhou. Journal of Tropical Medicine. 2018; 18(05): 565-8.

163. Gu SH, Arai S, Yu HT, Lim BK, Kang HJ, Yanagihara R. Genetic variants of Cao Bang hantavirus in the Chinese mole shrew (*Anourosorex squamipes*) and Taiwanese mole shrew (*Anourosorex yamashinai*). Infect Genet Evol. 2016; 40: 113-8.

164. Wang H, Yoshimatsu K, Ebihara H, Ogino M, Araki K, Kariwa H, et al. Genetic diversity of hantaviruses isolated in china and characterization of novel hantaviruses isolated from *Niviventer confucianus* and *Rattus rattus*. Virology. 2000; 278(2): 332-45.

165. Tian H, Tie WF, Li H, Hu X, Xie GC, Du LY, et al. Orthohantaviruses infections in humans and rodents in Baoji, China. PLoS Negl Trop Dis. 2020; 14(10): e0008778.

166. Luo ZZ, Liu Y, Liu H, Wang YY, Zhao YP. Discovery of two sub-types of hantanvirus in Anhui mountain areas. Chin J Epidemiol. 2002; 23(5): 363-5.

167. Liu SW, Xu G, Gong T, Shi Y, Xiao F, Li JX, et al. Detection and genetic analysis of Dabieshan orthohantavirus from 537 rodent lung samples in Jiangxi province. Modern Preventive Medicine. 2018; 45(20): 3778-81.

168. Lin XD, Wang W, Guo WP, Zhang XH, Xing JG, Chen SZ, et al. Cross-species transmission in the speciation of the currently known murinae-associated hantaviruses. J Virol. 2012; 86(20): 11171-82.

169. Hu Q, Zou CY, Ma SJ, Tong SM, Mei Y. Sequence comparison and analysis of S gene of five hantavirus strains separated from rodents. Chin J Zoono. 2016; 32(5): 502-5.

170. Cao ZW, Zuo SQ, Gong ZD, Zhan L, Bian CL, Zhang PH, et al. Genetic analysis of a hantavirus strain carried by Niviventer confucianus in Yunnan province, China. Virus Res. 2010; 153(1): 157-60.

171. Zou Y, Xiao QY, Dong X, Lv W, Zhang SP, Li MH, et al. Genetic analysis of hantaviruses carried by reed voles *Microtus fortis* in China. Virus Res. 2008; 137(1): 122-8.

172. Zou Y, Wang JB, Gaowa HS, Yao LS, Hu GW, Li MH, et al. Isolation and genetic characterization of hantaviruses carried by *Microtus voles* in China. J Med Virol. 2008; 80(4): 680-8.

173. Zhang YZ, Zou Y, Yan YZ, Hu GW, Yao LS, Du ZS, et al. Detection of phylogenetically distinct Puumala-like viruses from red-grey vole Clethrionomys rufocanus in China. J Med Virol. 2007; 79(8): 1208-18.

174. Wu YP, Huang B, Hu GW, Yao LS, Gou WM, Feng GD, et al. Analysis on epidemiologic features of HFRS in Jilin province. Chinese Journal of Control of Endemic Disenaces. 2004; 19(3): 164-5.

175. Wu DL, Wang H, Shen B, Hu GW, Li DX. RT-PCR detection and sequence analysis of Puumala virus in Jilin province. Chin J Publ Heal Engineering. 2008; 7(5): 259-61.

176. Wu DL, Wang H, Shen B, Hu GW, Hou X. Detection of immunoglobulin G antibody of hemorrhagic fever patients by an ELISA based on recombinant nucleocapsid protein of Puumala virus. Chinese Journal of Health Laboratory Technology. 2010; 20(3): 584-5.

177. Tang LH, Zhang QF, Xiu MH, Hu GW, Shen B, Yang XD, et al. Identification of a new Puumala hantavirus subtype in rodents from China. Chin J Virol. 2007; 23(4): 320-5.

178. Tang LH. Genetic analysis of new subtype of Puumala virus in China [Master Dissertation]. Beijing: Chinese Center for Disease Control and Prevention, **2007**.

179. Liu G, Li C, Hu GW, Li Y, Yao LS, Chen YQ, et al. Identification of Puumala like viruses in China. Chin J Exp Clin Virol. 2003; 17(1): 55-7.

180. Liu G. The molecular biology study of Puumala virus and the establishment of its diagnostic method [Master Dissertation]. Beijing: Chinese Center for Disease Control and Prevention, **2003**.

181. Li Q, Luo F, Yang ZQ. Humoral cross-reactivity of hantaan viruses to the nucleocapsid protein of Puumala viruses and Dobrava viruses in Hubei province. Immunol J. 2008; 24(4): 438-41.

182. Li JL. The molecular epidemiology study of Puumala virus and the establishment of its detection method [Master Dissertation]. Beijing: Chinese Center for Disease Control and Prevention, **2005**.

183. Geng YZ, Yao WQ, Liu Y, Sun YW, Wang B, Han YH, et al. Detection and gene feature analysis of Puumala virus in Liaoning province. Journal of Parasitic Biology. 2012; 07(6): 426-8.

184. Zhang WY, Jiang JF, Yao K, Wu XM, Zuo SQ, Zhan L, et al. Identincation of Amur like virus in Apodemus Peninsulae and its molecular characteristics in China. Chin J Epidemiol. 2007; 28(5): 482-6.

185. Yao LS, Shao LJ, Wang G, Liu Y, Liu YX, Pu Y, et al. Detection of Amur like virus in Apodemus peninsulae in Changbai county, Jilin province. Acta Parasitology et Medica Entomologica Sinica. 2013; 20(2): 120-4.

186. Wang Y, Fang ZQ, Li Y, Gao YF, Liu YY, Zhang XL, et al. Investigation and gene analysis of rodent-borne hantavirus at Changbai port in 2019. Chinese Journal of Hygienic Insecticides & Equipments. 2020; 26(6): 546-8.

187. Zuo SQ, Gong ZD, Fang LQ, Jiang JF, Zhang JS, Zhao QM, et al. A new hantavirus from the stripe-backed shrew (*Sorex cylindricauda*) in the People's Republic of China. Virus Res. 2014; 184: 82-6.

188. Zhang Y, Yuan J, Yang X, Zhou J, Yang W, Peng C, et al. A novel hantavirus detected in Yunnan red-backed vole (Eothenomys miletus) in China. J Gen Virol. 2011; 92(Pt 6): 1454-7.

189. Wang JL, Zhang HL, Zhou JH, Yang WH, Zhang YZ, Mi ZQ, et al. First detection of Tula-like hantanvirus from Eothenomys Milelus in China. Chin J Zoono. 2010; 26(5): 408-12.

190. Guo WP, Lin XD, Wang W, Tian JH, Cong ML, Zhang HL, et al. Phylogeny and origins of hantaviruses harbored by bats, insectivores, and rodents. PLoS Pathog. 2013; 9(2): e1003159.

191. Lin XD, Zhou RH, Fan FN, Ying XH, Sun XY, Wang W, et al. Biodiversity and evolution of Imjin virus and Thottapalayam virus in Crocidurinae shrews in Zhejiang province, China. Virus Res. 2014; 189: 114-20.

192. Guo WP, Lin XD, Wang W, Zhang XH, Chen Y, Cao JH, et al. A new subtype of Thottapalayam virus carried by the Asian house shrew (*Suncus murinus*) in China. Infect Genet Evol. 2011; 11(8): 1862-7.

193. Qi R, Sun XF, Qin XR, Wang LJ, Zhao M, Jiang F, et al. Suggestive serological evidence of infection with shrew-borne Imjin virus (Hantaviridae) in humans. Viruses. 2019; 11(12).

194. Ge XY, Yang WH, Pan H, Zhou JH, Han X, Zhu GJ, et al. Fugong virus, a novel hantavirus harbored by the small oriental vole (*Eothenomys eleusis*) in China. Virol J. 2016; 13: 27.

195. Xu L, Wu J, Li Q, Wei Y, Tan Z, Cai J, et al. Seroprevalence, cross antigenicity and circulation sphere of bat-borne hantaviruses revealed by serological and antigenic analyses. PLoS Pathog. 2019; 15(1): e1007545.

196. Xu L, Wu J, He B, Qin S, Xia L, Qin M, et al. Novel hantavirus identified in black-bearded tomb bats, China. Infect Genet Evol. 2015; 31: 158-60.

197. Zhu GHX, Meng YC, Lan MY. Natural infection in and transmission of EHF virus through biting by gamasid mites, Tricholaelaps myonyssognathus and Eulaelaps stabularis. Chin J Publ Heal. 1987; (06): 335-6.

198. Zhu CF, Yu CX, Sheng SQ, Jiang CM. Surveillance and analysis of hemorrhagic fever with renal syndrome in Fengtai county from 1982 to 1993. Chin J Vector Biol Control. 1995; (02): 100-4.

199. Zhou XF, Peng WJ, Zhang RL, Chen HB, Cao H. Investigation of rodents and hantavirus in Longhua district of Shenzhen from 2014 to 2019. Chinese Journal of Frontier health and Quarantine. 2020; 43(2): 95-7.

200. Zhou H, He JH, Li Y, Ning DM, Li GY. Surveillance and analysis of hemorrhagic fever with renal syndrome in Yunnan province in 1998. Chin J Publ Heal. 1999; (07): 1.

201. Zhao ZY, Fu GM, Weng JQ, Yao PP, Li MH, Lu QY, et al. Surveillance and analysis of HFRS in Zhejiang province from 1997 to 2000. Chin J Vector Biol Control. 2001; (05): 355-7.

202. Zhao ZY, Fu GM, Weng JQ, Yao PP, Li MH, Lu QY, et al. Surveillance and analysis of HFRS in Zhejiang province. Chin J Vector Biol Control. 2000; (04): 290-2.

203. Zhao XZ, Zhang Y. Studies of gamasid mite as the vector reservoir of epidemic hemorrhagic fever. Chin J Vector Biol Control. 1991; (05): 316-8.

204. Zhang Z, Ma XJ, Liu XB, Bai SY, Yan Y, Zhang B. Survey of the species, density and virus rate of hemorrhagic fever with renal syndrome of rodents in Ningxia Jingyuan. Ningxia Medical Journal. 2009; 31(10): 902-3.

205. Zhang YL, Yan J. Surveillance and analysis of hemorrhagic fever with renal syndrome in Pingliang city in 2003. Chinese Journal of Natural Medicine. 2005; (01): 37-8.

206. Zhang Y, Zhu J, Wu GH, Zhang LL, Sun ZJ, Zhang JJ, et al. Detection of hemorrhagic fever with renal syndrome virus structural protein and gene in gamasid mite and chigger mite. Chin J Vector Biol Control. 1999; (04): 3.

207. Zhang Y, Zhu J, Wu GH, Zhang JJ, Zhou YP. Study on detection of hemorrhagic fever with renal syndrome virus gene in cultured cells of gasmid mites and chigger mites. Chin J Publ Heal. 2000; (12): 2.

208. Zhang Y, Zhu J, Deng XZ, Wu GH, Zhang JJ, Zhou YP. Experimental study on the roles of gasmid mite and chigger mite in the transmission of hemorrhagic fever with renal syndrome virus. Chin J Epidemiol. 2001; (05): 3.

209. Zhang Y, Zhao XZ, Zhang BG, Shen JZ, Tang JQ, Bao MR, et al. Investigation of Crocidura Attenata as host animal of epidemic hemorrhagic fever. Chin J Publ Heal. 1987; 6(04): 209-10.

210. Zhang Y, Li FQ, Shen JZ, Bao MR, Deng XZ, Zhao XZ, et al. Investigation on natural infection of gamasid mite with epidemic hemorrhagic fever virus. Jinagsu Medical Journal. 1985; (06): 2-4.

211. Zhang WD, Tang WT, Liu AY, Pan XW, Yang SF. Surveillance of gamasid mites in epidemic hemorrhagic fever area of Daxian county. Chin J Vector Biol Control. 1994; (02): 1.

212. Zhang T, Miao XM, Li N, Liu XH, Zhang WX. Analysis of surveillance data of hemorrhagic fever with renal syndrome in Pingyi county from 1980 to 2000. Preventive Medicine Tribune. 2002; (05): 618-9.

213. Zhang R, Yao PP, Xu F, Sun JM, Lv HK, Lu MG, et al. Analysis of epidemiological characteristics and surveillance of hemorrhagic fever with renal syndrome in Zhejiang province, China, 2013. Chin J Vector Biol Control. 2015; 26(01): 37-40.

214. Zhang ML, Wang L. Analysis of surveillance results of hemorrhagic fever with renal syndrome in Ganyu county in 2005. Chinese Primary Health Care. 2007; (02): 66-7.

215. Zhang M, Yan Y, Qin ZQ, Wang L, Ma HR, Ma XM. Analysis of host animal surveillance results of hemorrhagic fever with renal syndrome in Jingyuan county, Ningxia in 2013. Ningxia Medical Journal. 2015; 37(03): 275-6.

216. Zhang LZ, Yin LQ, Li MM, Li BR. Surveillance and epidemic analysis of hemorrhagic fever with renal syndrome in Chifeng city from 2005 to 2009. Journal of Medical Pest Control. 2010; 26(12): 1158-9.

217. Zhang LZ, Yin LQ, Chen DL. Surveillance and epidemic analysis of hemorrhagic fever with renal syndrome in Chifeng city from 1998 to 2005. Journal of Medical Pest Control. 2006; (01): 13-4.

218. Zhang L, Yang FT, You DQ, Lu Q, Jin J. Investigation on the latent infection of hemorrhagic fever with renal syndrome among healthy individuals in the epidemic foci in Hefei city. Chinese Journal of Disease Control & Prevention. 2015; 19(07): 671-4.

219. Zhang GB, Ying LH, Zhang XY, Hu XX, Wen L. Surveillance and analysis of hantavirus recessive infection rate in healthy population in Jinyun county. Practical Preventive Medicine. 2016; 23(03): 354-5.

220. Zeng YW, Xie Y, Xiong Y, Gong T, Zhou J, Shi Y, et al. Analysis on survrillance of HFRS in Gaoan city from 2006 to 2007. Modern Preventive Medicine. 2009; 36(16): 3148-9+55.

221. Yuan M, Li D, Wang Q. Epidemic analysis of hemorrhagic fever with renal syndrome in Anyi county from 2010 to 2014. Jiangsu Journal of Preventive Medicine. 2016; 27(02): 192-3.

222. Yu ZY. Surveillance and analysis of hemorrhagic fever with renal syndrome in Quzhou city, Zhejiang province from 2005 to 2012. Dis Surveill. 2013; 28(11): 929-32.

223. Yu LZ, Fu JR, Wang YP. Analysis of surveillance results of hemorrhagic fever with renal syndrome from 2006 to 2010 in Ningxiang county. Practical Preventive Medicine. 2012; 19(02): 207-8.

224. Ying LH, Zhang GB, Zhang XY, Hu XX, Wen L. Analysis of surveillance results of hemorrhagic fever with renal syndrome from 2005 to 2014 in Jinyun county, Zhejiang province. China Preventive Medicine. 2016; 17(11): 846-8.

225. Yin LQ, Chen DL, Wang MY. Surveillance and epidemic analysis of hemorrhagic fever with renal syndrome in Chifeng city from 1998 to 2001. Journal of Medical Pest Control. 2002; (06): 281-2.

226. Yang YH. Surveillance on hemorrhagic fever with renal syndrome in Bayannaoer from 2000 to 2005. Preventive Medicine Tribune. 2006; (06): 727-8.

227. Xia YF. Host animal surveillance and human epidemic analysis of hemorrhagic fever with renal syndrome in Ji 'an from 2003 to 2007. Chinese Primary Health Care. 2008; 22(12): 47-8.

228. Wu JL, Su YC, Cai MS, Qiu QH, Wang TT, Dong CF. Surveillance and analysis of hemorrhagic fever with renal syndrome in Shishi city from 2007 to 2015. Strait J Prev Med. 2016; 22(06): 35-7.

229. Wu AL, Chen MF, Li YH. Surveillance and analysis of hemorrhagic fever with renal syndrome in Dongyang city, Zhejiang province from 2005 to 2011. Chin J Vector Biol Control. 2013; 24(01): 75.

230. Wen JL, Xie GL. Surveillance of hemorrhagic fever with renal syndrome in Gaoan city in 2000. Chin J Vector Biol Control. 2002; (01): 74.

231. Wang Z, Xu ZY, Sun L, Liang CL, Ma B. Analysis on HFRS surveillance in Yingshang county during 2010-2015. Journal of Diseases Monitor & Control. 2016; 10(06): 439-41.

232. Wang LG. Analysis of 10 years surveillance of hemorrhagic fever with renal syndrome in Dafeng city. Jiangsu Journal of Preventive Medicine. 2001; (01): 34-5.

233. Wang DM, Wang ZX, Tong YB, Liu M, Hu LJ, Cai XH, et al. Surveillance of host animals and analysis of human epidemic situation of hemorrhagic fever with renal syndrome in Zunyi county from 1996 to 2000. Chin J Vector Biol Control. 2003; 14(5): 384-5.

234. Sun MH, Li ZQ, Li BX, Yang HN, Ma ST, He C. Survey on rats and pathogens at Ji¡¯an road bridge port in 2016. Chinese Journal of Frontier health and Quarantine. 2018; 41(1): 21-3.

235. Shi YL, Gao XD, Liang XC, Yao CX, Ding XL, Xi JX, et al. The investigaiton and analysis of epidemiology hemorrhagic fever in Minxian county, Gansu. Endemic Dis Bull. 1997; (02): 67-9+6.

236. She JJ, Zhang Y, Huang ZA, Yu MM, Jiang KJ, Wu GH. Preliminary study on Leptotrombidium subpalpale as spreading medium of HFRS. Chin J Vector Biol Control. 1998; (01): 4.

237. Rao HY. Epidemiological characteristics and surveillance analysis of hemorrhagic fever with renal syndrome in Shunchang county from 2004 to 2019. Strait J Prev Med. 2020; 26(06): 37-9.

238. Qiao YL. Monitoring and analysis of serum antigen and antibody levels in rats of main infection source of hemorrhagic fever with renal syndrome in Zhengyang county by ELISA. Journal of Diseases Monitor & Control. 2021; 15(3): 182-3,213.

239. Qian JY, Deng XZ, Zhang Y. Investigation of Leptotrombidium scutellare-hemorrhagic fever with renal syndrome vector. Chin J Vector Biol Control. 2009; 20(06): 583-4.

240. Pang WL, Zheng X, Ge JH, Cui QR, Liu Y, Hu YF, et al. Surveillance of hemorrhagic fever with renal syndrome in Tiantai county of Zhejiang province, China, from 1984 to 2018. Chin J Vector Biol Control. 2020; 31(02): 158-63.

241. Pang WL, Cui QR, Qiu XQ, Ge JH, Chen Y. Analysis of surveillance results of hemorrhagic fever with renal syndrome from 2001 to 2011 in Tiantai county, Zhejiang province. Chin J Vector Biol Control. 2012; 23(06): 574-6.

242. Luo YX, Jiang QY, Liu MQ, Cai XH. Surveillance and epidemic analysis of host animals of hemorrhagic fever with renal syndrome in Guiyang city. Journal of Applied Preventive Medicine. 2002; (05): 297-9.

243. Liu LQ, Wu CX, Chen JY. Investigation on the relationship between seasonal fluctuation of rodent mite and incidence of epidemic hemorrhagic fever. Journal of Medical Pest Control. 1992; (01): 36-40+4.

244. Liu LQ, Chen JY, Wu CX, Ou YW, Liu G, Huang XF. Investigation on epidemic hemorrhagic fever foci in Hejiashan township, Zixing city. Journal of Clinical Research. 1990; (02): 114.

245. Lin YZ, Yang W, Liu PQ. Serosurvey of epidemic hemorrhagic fever on Hainan isand. J Hainan Med Univ. 2000; (02): 75-6.

246. Li ZY, Zhu LY, Zhang LJ, Li H, Wang ZH. Analysis of HFRS surveillance from 1999 in Fuyang. Anhui Journal of Preventive Medicine. 2000; (03): 177-9.

247. Li T, Wei XJ, Zhang HL, Chen Z. Surveillance and analysis of hemorrhagic fever with renal syndrome and evaluation of vaccine immunization effect in Fengcheng city, Jiangxi province in 2014. South China Journal of Preventive Medicine. 2017; 43(02): 145-7.

248. Li KL, Zheng ZS, Xie YW. The surveillance of rats and pathogens at Foshan Chencun Guotong logistics city, 2016. Chinese Journal of Frontier health and Quarantine. 2017; 40(2): 106-7.

249. Li DH, Guo RQ. Analysis of surveillance data of hemorrhagic fever with renal syndrome in Ganyu county in 2001. Preventive Medicine Tribune. 2003; (01): 112-3.

250. Jiang WB. Comprehensive analysis of monitoring and prevention of hemorrhagic fever with renal syndrome in Jiujiang city. Jiujiang Medical Journal. 2000; (03): 173-4.

251. Jiang MJ, Wang YA, Wu LF. Surveillance and analysis of hemorrhagic fever with renal syndrome in Linshu county. Journal of Medical Pest Control. 1999; (07): 366-7.

252. Jian HH, Wan JF, Zhu LY, Jiang T, Tian YZ. Analysis on the surveillance of hemorrhagic fever with renal syndrome in Fuyang city from 2006 to 2010. Anhui Journal of Preventive Medicine. 2012; 18(01): 31-3.

253. Huang JG. Analysis of surveillance results of hemorrhagic fever with renal syndrome in Dafeng city in 1998. Chin J Publ Heal. 1999; (07): 1.

254. He XQ, Li WR, Zhao YP, Ren CZ, Liu H. A report of EHF recessive infection among healthy people in Tibet Autonomous region. Anhui Journal of Preventive Medicine. 1997; (3): 17.

255. He PS, Ma YK, Li XG, Zhang JT. Analysis of surveillance data of hemorrhagic fever with renal syndrome in Junan county from 1975 to 1998. Journal of Shandong Medical College. 1999; (02): 99-101.

256. Gu LQ, Zheng JF, Zhou YF, Zhang XL, Zhang L, Hu XB. Investigation on epidemic hemorrhagic fever in Yadong county Xizang. Medical Journal of National Defending Forces in Southwest China. 2021; 31(1): 87-9.

257. Gong ZY, Zhao ZY, Weng JQ, Fu GM, Jiang LP. Surveillance and analysis of hemorrhagic fever with renal syndrome in Zhejiang province in 1997. Chin J Epidemiol. 1998; (06): 1.

258. Gao YX, Li XB, Fang SF, Yan J, Huang L, Zhang XG, et al. Investigation of Yersinia pestis and hantavirus carried by small mammal hosts at Guangdong entry-exit ports. Chin J Vector Biol Control. 2016; 27(2): 137-40.

259. Fu JR, Hu JZ, Guo LJ, Huang CH, Long QZ, Liu YZ, et al. Monitoring of hemorrhagic fever with renal syndrome in Ningxiang county in 1998. Chin J Publ Heal. 1999; (07): 1.

260. Fu GM, Zhao ZY, Weng JQ, Zhu ZY. Analysis on the surveillance of hemorrhage fever with renal syndrome of Zhejiang province in 1998. Chin J Vector Biol Control. 1999; (04): 3.

261. Fu GM, Zhao ZY, Weng JQ, Lu QY, Yao PP, Li MH, et al. Surveillance of hemorrhagic fever with renal syndrome (HFRS) in Zhejiang province. Dis Surveill. 2001; (08): 285-6.

262. Fu GM, Gao Y, Fang CF, Zhao ZY, Zhu ZY. Analysis of hemorrhagic fever with renal syndrome surveillance in Kaihua county of Zhejiang province. Chin J Vector Biol Control. 2004; (03): 214-5.

263. Fang CF, Yu ZY, Wang W. Surveillance and analysis of hemorrhagic fever with renal syndrome (HFRS) from 2002 to 2004 in Quzhou city. Dis Surveill. 2005; (10): 513-6.

264. Chen SL, Wang JY. Analysis of surveillance data of hemorrhagic fever with renal syndrome in Zhoushan city from 1985 to 2000. Preventive Medicine Tribune. 2002; 8(1): 102-3.

265. Chen JJ, Tang F, Wang XQ, Zhang ZZ, Chu CY, Wang N, et al. Investigation of rodent borne pathogens in scenic areas of Beijing. Military Medical Sciences. 2020; 44(2): 122-30.

266. Chen HX, Xie SH, Li SQ, Luo CW, Tang LY, Yu T, et al. Seroepidemiological survey on natural epidemic areas of hemorrhagic fever with renal syndrome in Linzhi areas of Tibet. Chin J Vector Biol Control. 2001; 12(2): 89-93.

267. Chang SJ. Surveillance and analysis of hemorrhagic fever with renal syndrome in Lintan county from 1994 to 1998. Dis Surveill. 1999; (09): 335-6.

268. Cai CL, Chen WS, Chen WH, Zhuo F, Yao XJ, Zhang RL. Serological investigation of rodents hantaviruses in epidemic foci of hemorrhagic fever with renal syndrome in Shenzhen. Chinese Journal of Health Laboratory Technology. 2017; 27(1): 127-8.

269. Bi ZQ, Wang ZQ, Wang XJ, Wang M, Ma WS, Song SX, et al. The first detection of hantavirus antigen in Sorex araneus in Shandong province. Chin J Epidemiol. 2005; (7): 484.

270. Zhao CS, Jiang LH, Chen WJ, Lu ZZ. Investigation of Xinjiang hemorrhagic fever virus antibody in human serum in some areas of Hainan province. Chin J Zoono. 1996; (04): 2.

271. Zhao CS, Bai ZJ, Peng YF, Zhao WZ, Chen CH, Fang MY, et al. The seroepidemiolgical investigation of arbovirus in humans and rats of the south of China. Chin J Publ Heal. 2001; (01): 3.

272. Zhang Y, Shen S, Fang Y, Liu J, Su Z, Liang J, et al. Isolation, characterization, and phylogenetic analysis of two new Crimean-Congo hemorrhagic fever virus strains from the northern region of Xinjiang province, China. Virol Sin. 2018; 33(1): 74-86.

273. Zhang T, Niu JQ, Jiang YF, Wang F. Clinical study of Xinjiang hemorrhagic fever in Jilin province. Chin J Exp Clin Infect Dis. 2007; (03): 158-60.

274. Xiao CD. Investigation on vectors of Xinjiang hemorrhagic fever. Endemic Dis Bull. 2004; (S1): 50-4.

275. Xia H, Li P, Yang J, Pan L, Zhao J, Wang Z, et al. Epidemiological survey of Crimean-Congo hemorrhagic fever virus in Yunnan, China, 2008. Int J Infect Dis. 2011; 15(7): e459-63.

276. Tu YR, Zhang JK, Yan DY, Zhang LL, Jin ZW, Liu XC, et al. A seroepidemiological survey on Xinjiang hemorrhagic fever (Crimean-congo hemorrhagic fever) in Sichuan province. Chin J Vector Biol Control. 1992; (06): 381-3.

277. Tong SD, Pan H, Ma YH, Jiang ZJ, Lu ZZ, Song Y, et al. Investigation of tick-borne zoonosis in dogs in hot areas. Chinese Journal of Veterinary Science. 1998; (04): 1.

278. Tang Q, Han L, Zhao XQ, Tao XX. Field investigation reports of Crimean-Congo hemorrhagic fever in Bachu, Xinjiang in 2001. Dis Surveill. 2002; 17(2): 50-.

279. Tang Q, Gao DP, Zhao XQ, Han L, Hang CS. Study on the molecular biology of hemorrhagic fever virus in Xinjiang. Chin J Epidemiol. 2002; 23(6): 449-52.

280. Sun SR, Hao JM, Luo T, Wang QG, Zhang YJ. Analysis correlation between Xinjiang hemorrhagic fever prevalence and ecosystem diversity of its natural foci in the a Trim rivers (II). Endemic Dis Bull. 2014; 29(02): 7-9+27.

281. Sun S, Dai X, Aishan M, Wang X, Meng W, Feng C, et al. Epidemiology and phylogenetic analysis of crimean-congo hemorrhagic fever viruses in Xinjiang, China. J Clin Microbiol. 2009; 47(8): 2536-43.

282. Shao XR, La Y, Xue WJ, Cheng Y, Wang XY, Wang JQ, et al. Natural foci of Xinjiang hemorrhagic fever detected in Qinghai province. Qinghai Medical Journal. 1995; (03): 2.

283. Shao XR, La Y, Cheng Y, Wang XY, Liu QM, Li YF, et al. The first case of tick-borne hemorrhagic fever virus antibody detected in Qinghai province. Qinghai Medical Journal. 1994; (02): 2.

284. Ren CZ, Wang YY, Liu H, Luo ZZ. First detection of Crimean-Congo hemorrhagic fever in Anhui province. Chin J Epidemiol. 1995; (01): 1.

285. Qi JS, Chen YB, Wu JJ, Wang M, Chen WZ, Feng CH, et al. The first detection of Crimean-Congo hemorrhagic fever virus IgG antibody in bovine serum in Hainan province. Chin J Vector Biol Control. 1995; (02): 158.

286. Pan H, Tong SD, Ma YH, Mo ZS, Dong WD, Hu YH, et al. Investigation on zoonosis in dogs and animals in South China military police. Chin J Zoono. 1998; (05): 92-3.

287. Na, Na, Na. Report on diagnosis and treatment of two cases of Xinjiang hemorrhagic fever in Bachu county in 2003. Endemic Dis Bull. 2007; (05): 63.

288. Moming A, Yue X, Shen S, Chang C, Wang C, Luo T, et al. Prevalence and phylogenetic analysis of Crimean-Congo hemorrhagic fever virus in ticks from different ecosystems in Xinjiang, China. Virol Sin. 2018; 33(1): 67-73.

289. Mao B. Analysis of clinical data of 55 cases of Xinjiang hemorrhagic fever. Endemic Dis Bull. 2004; (S1): 58-61.

290. Lv XJ, Tang Q, Feng YM, Zhi Q, Wang C, Xu LH, et al. Molecular biological survey of tick-born arboviruses in southern part of Xinjiang. Chin J Exp Clin Virol. 2005; 19(4): 325-30.

291. Liu YH, Li WH, Liu YZ, Feng CH, Zhou HY, Liu XF. Investigation on a nosocomial outbreak of Xinjiang hemorrhagic fever (XHF). Endemic Dis Bull. 2004; (S1): 31-4.

292. Liu YH, Chai JJ, Xiao CD, Li WH. Epidemiological analysis of 140 cases with Xinjiang hemorrhagic fever. Endemic Dis Bull. 2004; (S1): 47-9.

293. Liu DX. The research of tick-borne Crimean-Congo hemorrhagic fever virus genomeand molecular epidemiology in the west of InnerMongolia££Š [Master Dissertation]. Yinchuan(Ningxia): Ningxia University, **2017**.

294. Li Y, Yan C, Liu D, He B, Tu C. Seroepidemiological investigation of Crimean-Congo hemorrhagic fever virus in sheep and camels of Inner Mongolia of China. Vector Borne Zoonotic Dis. 2020; 20(6): 461-7.

295. Li WH, Liu YH, Ji XS, Liu XF. Epidemiological survey of Xinjiang hemorrhagic fever. Endemic Dis Bull. 2004; (S1): 82-3.

296. Li LH, Zhang YP, Wu ZY, Hao ZY, Tang Q, Tao XX, et al. Sero-epidemiological investigation of Xinjiang hemorrhagic fever in Henan province. China Preventive Medicine. 2005; (05): 426-8.

297. Kou C. Investigation of arboviruses from ticks and rodents in the surrounding region of Xinjiang Junggar Basin [Master Dissertation]. Urumchi(Xinjiang): Xinjiang University, **2016**.

298. Ji XS, Li WH, Liu XF, Mu JD. Survey of animal host of Xinjiang hemorrhagic fever virus in Bachu county, Xinjiang in 1979. Endemic Dis Bull. 2004; (S1): 76-8+125.

299. Huang HZ, Chen YB, Feng CH, Chen HX, Chen WZ, Kuang JS, et al. Analysis on testing result of serum antibody against CCHFV virus in Hainan province. Chin J Vector Biol Control. 1996; (05): 2.

300. Hou ZL, Huang WL, Zi DY, Zhang HL, Shi HF, Gong ZD, et al. Study on serological epidemiology of tick-borne viruses in Yunnan. Chin J Vector Biol Control. 1992; (03): 173-6.

301. Han L, Tang Q, Zhao XQ, Xi TZX, Tao XX. Serologic studies of Xinjiang hemorrhagic fever in Bachu county, 2001. Chin J Epidemiol. 2002; (03): 3.

302. Guo R, Shen S, Zhang Y, Shi J, Su Z, Liu D, et al. A new strain of Crimean-Congo hemorrhagic fever virus isolated from Xinjiang, China. Virol Sin. 2017; 32(1): 80-8.

303. Feng CH, Liu XF, Wang DL, Bai XH, Zhang XB, Liu QM, et al. Report on surveillance of Xinjiang Hemorrhagic Fever in Bachu area of Xinjiang in 1988. Endemic Dis Bull. 2004; (S1): 84-7+125.

304. Feng CH, Liu XF, Gu Y, A DL, Liu Y, Fu JP. A survey on natural foci of Xinjiang hemorrhagic fever in Bachu county of Xinjiang. Endemic Dis Bull. 1988; (02): 66-9.

305. Feng CH, Li WH, Liu XF, An GT, Xiao CD, Zhang YZ, et al. A summary of the work on pathogeny of Xinjiang hemorrhagic fever in 1975. Endemic Dis Bull. 2004; (S1): 55-7.

306. Feng CH, Li F, Bai XH, Liu ZD, Liu HB, Zhang F. Investigation of hemorrhagic fever in Xinjiang border area of Ili River Valley. Endemic Dis Bull. 1994; (03): 2.

307. Feng CH, Bai XH, Liu HB, Li F, Gu Y. Discovery of natural foci of Xinjiang hemorrhagic fever in the southern Margin area of Junggar Basin, Xinjiang. Endemic Dis Bull. 1991; (01): 52-5.

308. Dong XM. Preliminary investigation on the carriage status of tick-arbovirus virus among the rodents and ticks in parts of Xinjiang area [Master Dissertation]. Urumchi(Xinjiang): Xinjiang University, **2015**.

309. Chen HX, Tang JQ, Liu RH, Ren Y, Wang HM, La Y, et al. The first detection of anti-Xinjiang hemorrhagic fever virus lgG antibody from sheep sera in Qinghai province. Chin J Vector Biol Control. 1991; (04): 267-9.

310. Chai JJ, Xiao CD, Liu YH, Zhang YH, Yu SD, Han YY, et al. Report of investigation on hemorrhagic fever in Bachu county, Xinjiang, in 1966 (¢ó- Investigation on epidemiology of hemorrhagic fever in Bachu). Endemic Dis Bull. 2004; (S1): 15-22+117-20.

311. Chai JJ, Han YY, Zhang YW. Report of five cases of viral hemorrhagic fever in Bachu area of Xinjiang. Chin J Infect. 1985; 3(4): 257-9.

312. Chai JJ, Feng CH, Yu SD, Li HG, Liu J. Natural infection of Xinjiang hemorrhagic fever virus in sheep. Endemic Dis Bull. 2004; (S1): 26-7.

313. Chai JJ. Preliminary report on survey of the distribution of hemorrhagic fever on the upper and middle reaches of the Tarim river. Endemic Dis Bull. 2004; (S1): 34-6.

314. Cai ZL, Lu ZX, Hu LM, Jin XT, He YX. Antibody to Xinjiang hemorrhagic fever virus detected in human serum in some areas of northeast China. Chin J Zoono. 1994; (05): 2.

315. Yang L, Zhao Z, Hou G, Zhang C, Liu J, Xu L, et al. Genomes and seroprevalence of severe fever with thrombocytopenia syndrome virus and Nairobi sheep disease virus in Haemaphysalis longicornis ticks and goats in Hubei, China. Virology. 2019; 529: 234-45.

316. Gong S, He B, Wang Z, Shang L, Wei F, Liu Q, et al. Nairobi sheep disease virus RNA in ixodid ticks, China, 2013. Emerg Infect Dis. 2015; 21(4): 718-20.

317. Li CX. Unprecedented genomic diversity of RNA viruses in arthropods reveals the ancestry of negative-sense RNA viruses [Ph.D Dissertation]. Beijing: Chinese Center for Disease Control and Prevention, **2015**.

318. Liu X, Zhang X, Wang Z, Dong Z, Xie S, Jiang M, et al. A tentative Tamdy orthonairovirus related to febrile illness in northwestern China. Clin Infect Dis. 2020; 70(10): 2155-60.

319. Dong Z, Yang M, Wang Z, Zhao S, Xie S, Yang Y, et al. Human Tacheng tick virus 2 infection, China, 2019. Emerg Infect Dis. 2021; 27(2): 594-8.

320. Wang YC, Wei Z, Lv X, Han S, Wang Z, Fan C, et al. A new nairo-like virus associated with human febrile illness in China. Emerg Microbes Infect. 2021; 10(1): 1200-8.

321. Ma J, Lv XL, Zhang X, Han SZ, Wang ZD, Li L, et al. Identification of a new *orthonairovirus* associated with human febrile illness in China. Nat Med. 2021; 27(3): 434-9.

322. Zhou H, Ma Z, Hu T, Bi Y, Mamuti A, Yu R, et al. Tamdy virus in *Ixodid* ticks infesting bactrian camels, Xinjiang, China, 2018. Emerg Infect Dis. 2019; 25(11): 2136-8.

323. Chen BQ, Liu QZ, Yang SC, Wang YL, Chang RX, Li ZL. Elevated Snowshoe hare virus antibody detected from the serum of patients with sporadic encephalitis in Guangzhou. Chin J Virol. 1985; (03): 283-5.

324. Gu HX, Spence L, Artsob H, Chia WK, Th'ng C, Lampotang V. Serological evidence of infection with California serogroup viruses (family *Bunyaviridae*) in residents of Long Hua, suburb of Shanghai, People's Republic of China. Trans R Soc Trop Med Hyg. 1984; 78(6): 780-1.

325. Gu HX, Na, Ren SL, Lin YZ, Zhou MD, Huang CH. Serological investigation on the infection of California group of viruses in China. Fudan University Journal of Medical Science. 1987; (02): 107-11.

326. Feng QM, Li CL, Zhang RZ, Zheng ZR. Preliminary report on sero-epidemiological investigation of Akabane disease. China Animal Health Inspection. 1991; (03): 3.

327. Feng Y, He B, Fu SH, Yang WH, Zhang YZ, Tu CC, et al. Isolation and identification of the Akabane virus from mosquitoes in Yunnan province, China. Chin J Virol. 2015; 31(01): 51-7.

328. Gu X, Zhai H, Liu DN, Li DY. Serological investigation on Akabane disease of dairy cows in Fengxian area. Animal husbandry and Veterinary Abstracts of China. 2016; 32(03): 118.

329. Li CL, Feng QM, Zhang RZ, Zheng ZR, Zhou LQ. Serological investigation of Akabane disease in China. China Animal Health Inspection. 1994; (02): 2.

330. Li QP, Zhou LQ, Yao LT, Cai YS, Chen XQ, Wei LX, et al. Isolation and preliminary identification of Akabane virus. China Animal Health Inspection. 2000; (07): 3.

331. Lin J, Qin SM, Bai AB, Cao YY, Rao GB, Wu JM. Survey of Akabane virus and bluetongue virus infections in Guangxi province. Chinese Journal of Preventive Veterinary Medicine. 2014; 36(10): 763-5.

332. Qiu Y, Zhu JM, Yu LJ. The detection of neutralizing antibodies against Akabane disease in Xinjiang cattle. Xinjiang Agricultural Sciences. 1997; (03): 139-40.

333. Ren PF, Wu JM, Lin J, Zhang YX, Dong YC, Qin SM. The seroepidemiology survey and influencing factors analysis of goats and cattle akabane disease in Guangxi province. Heilongjiang Animal Science and Veterinary Medicine. 2018; (20): 96-9.

334. Song S. Arbovirus isolation and identification of mosquitoes and midges collected in Guizhou and Sichuan province in China [Master Dissertation]. Qingdao(Shandong): Qingdao University, **2018**.

335. Tang HB, Chen F, Rao G, Bai A, Jiang J, Du Y, et al. Characterization of Akabane virus from domestic bamboo rat, Southern China. Vet Microbiol. 2017; 207: 280-5.

336. Wei LX, Yao LT, Gu BL. Serological investigation of Akabane disease in dairy cows. Shanghai Journal of Animal Husbandry and Veterinary Medicine. 1999; (05): 25.

337. Yang SY, Gao FK, Feng YZ. Preliminary report on epidemiological investigation of Akabane disease of cattle and sheep in Tianjin. China Animal Health Inspection. 1995; (01): 2.

338. Wu D, Zhang X, Zhang H, Tan QQ, Zhou HQ, Wang HY, et al. Detection and molecular characterization of the Akabane virus and Oya virus in Guangdong province, China. Chin J Virol. 2020; 36(01): 84-91.

339. Fu GH, Liu RC, Huang Y, Fu QL, Liu QH, Cheng LF, et al. Identification of Batai virus isolated from breeding Muscovy Duck. Chinese Journal of Preventive Veterinary Medicine. 2017; 39(4): 315-8.

340. Liu H, Hu B, Zhang L, Li XT, Deng XY, Yan XJ. Development and application of an indirect ELISA method for detection of antibody to cattle Batai virus. Special Wild Economic Animal and Plant Research. 2017; 39(4): 25-7.

341. Liu H, Shao XQ, Hu B, Zhao JJ, Zhang L, Zhang HL, et al. Isolation and complete nucleotide sequence of a Batai virus strain in Inner Mongolia, China. Virol J. 2014; 11: 138.

342. Liu RC, Huang Y, Lu RH, Fu GH, Fu QL, Shi SH, et al. Development of an indirect ELISA for detecting antibody against duck Batai virus (BATV) and preliminary survey of BATV in same regions. Chinese Journal of Preventive Veterinary Medicine. 2016; 38(12): 925-9.

343. Zhang HL, Tao SJ, Yang DR, Zhang YZ, Yang WH, Zhang YZ, et al. Isolation of Sindbis, Batai and Colti virus in Yunnan province. Chin J Zoono. 2005; 21(7): 548-51,57.

344. Zhang LJ, Zhang QS, Fu GH, Wang J, Hu XY, Huang Y, et al. Isolation and identification of a duck-origin Batai virus. Proceedings of the 16th Symposium of the Chinese Society of Animal Infectious Diseases. Jinan, China, **2015**:192-.

345. Zhang YZ, Zhang HL, Yang WH, Feng Y. Biological characters of Batai virus isolated formed from Yunnan province. Chin J Zoono. 2013; 29(6): 527-32.

346. Zhang J, Wang J, Wang L, Fu S, Li M, Zhao G, et al. Molecular characterization and seroprevalence in pigs of SC0806, a Cat Que virus isolated from mosquitoes in Sichuan province, China. Vector Borne Zoonotic Dis. 2015; 15(7): 423-31.

347. Cao Y, Fu S, Tian Z, Lu Z, He Y, Wang H, et al. Distribution of mosquitoes and mosquito-borne arboviruses in Inner Mongolia, China. Vector Borne Zoonotic Dis. 2011; 11(12): 1577-81.

348. Li W, Cao Y, Fu S, Wang J, Li M, Jiang S, et al. Tahyna virus infection, a neglected arboviral disease in the Qinghai-Tibet plateau of China. Vector Borne Zoonotic Dis. 2014; 14(5): 353-7.

349. Li WJ. Mosquitoes and mosquitoes-borne arboviruses and the relation with local human disease in Qinghai province [Ph.D Dissertation]. Jinan(Shandong): Shandong University, **2011**.

350. Li WJ, Wang JL, Li MH, Fu SH, Wang HY, Wang ZY, et al. Mosquitoes and mosquito-borne arboviruses in the Qinghai-Tibet plateau-focused on the Qinghai area, China. Am J Trop Med Hyg. 2010; 82(4): 705-11.

351. Lu Z, Fu SH, Wang FT, Nasci RS, Tang Q, Liang GD. Circulation of diverse genotypes of Tahyna virus in Xinjiang, People's Republic of China. Am J Trop Med Hyg. 2011; 85(3): 442-5.

352. Lu Z, Lu XJ, Fu SH, Zhang S, Li ZX, Yao XH, et al. Tahyna virus and human infection, China. Emerg Infect Dis. 2009; 15(2): 306-9.

353. Lv Z. Arboviruses investigation in Xinjiang [Ph.D Dissertation]. Beijing: Chinese Center for Disease Control and Prevention, **2009**.

354. Lv Z, Fu SH, Wang FT, Kosoy OL, Nasci RS, Liang GD. Investigation of Tahyna virus infection among unknown fever cases in Xinjiang, China. Chin J Virol. 2011; 27(01): 71-4.

355. Feng Y, Fu SH, Yang WH, Zhang YZ, He B, Tu CC, et al. Isolation and full-length genome analysis of mosquito-borne Manzanilla virus from Yunnan province, China. BMC Res Notes. 2015; 8: 255.

356. Zhang HL, Zhang YZ, Yang WH, Feng Y, Nasci RS, Yang J, et al. Mosquitoes of western Yunnan province, China: seasonal abundance, diversity, and arbovirus associations. PLoS One. 2013; 8(10): e77017.

357. Liu R, Zhang GL, Sun X, Zheng Z, Liu XM, Zhao Y, et al. Isolation and molecular characterization on Abbey Lake Orthobunyavirus (Bunyaviridae) in Xinjiang, China. Chin J Epidemiol. 2014; 35(8): 939-42.

358. Xia H, Liu R, Zhao L, Sun X, Zheng Z, Atoni E, et al. Characterization of Ebinur lake Virus and Its Human Seroprevalence at the China-Kazakhstan Border. Front Microbiol. 2019; 10: 3111.

359. Cai CL, Chen WS, Yang F, Zhang RL, Zhuo F, Gu WZ. Serological investigation of SFTS bunyavirus among possible epidemic hemorrhagic fever cases in Shenzhen. Chinese Journal of Health Laboratory Technology. 2015; 25(12): 2011-2+5.

360. Cai L, Zhang H, Gao LD, Hu SX, Xie LY, Zhan ZF, et al. Identification of the first case of SFTSV infection in the Hunan province of China and epidemiological surveillance in the locality. Ticks Tick Borne Dis. 2019; 10(2): 454-61.

361. Cui F, Cao HX, Wang L, Zhang SF, Ding SJ, Yu XJ, et al. Clinical and epidemiological study on severe fever with thrombocytopenia syndrome in Yiyuan county, Shandong province, China. Am J Trop Med Hyg. 2013; 88(3): 510-2.

362. Ding S, Yin H, Xu X, Liu G, Jiang S, Wang W, et al. A cross-sectional survey of severe fever with thrombocytopenia syndrome virus infection of domestic animals in Laizhou city, Shandong province, China. Jpn J Infect Dis. 2014; 67(1): 1-4.

363. Ding XL, Zhan JB. Survey on the infectious status of population with severe fever with thrombocytopenia syndrome bunyavirus in Macheng city, Hubei province in 2013. Practical Preventive Medicine. 2014; 21(06): 681-2.

364. Dong YH, Huang XY, Wang HF, You AG, Hu XN, Kang K, et al. Investigation of animals infected with novel bunyavirus In Xinyang city, Henan province, China. Chin J Zoono. 2014; 30(07): 766-8.

365. Du Y, Cheng N, Li Y, Wang H, You A, Su J, et al. Seroprevalance of antibodies specific for severe fever with thrombocytopenia syndrome virus and the discovery of asymptomatic infections in Henan province, China. PLoS Negl Trop Dis. 2019; 13(11): e0007242.

366. Ge JH, Pang WL, Sun JM, Zhou XB, Hu HY, Wang LY. Epidemiological characteristics of severe fever with thrombocytopenia syndrome and its inapparent infection inTiantai county, Zhejiang province. Chin J Zoono. 2017; 33(09): 848-52.

367. Gong L, Jiang M, Liu J, Han W, Liu J, Sun Z, et al. [Prevalence and homology analysis on human and animals severe fever with thrombocytopenia syndrome virus infection in Yantai of Shandong province]. Chin J Epidemiol. 2014; 35(5): 524-7.

368. Gu S, Wu X, Zhou B, Ling F, Zhang H, Huang Y, et al. [Epidemiological investigation on an outbreak of severe fever with thrombocytopenia syndrome in northwest Zhejiang province]. Chin J Epidemiol. 2015; 36(4): 364-7.

369. He WH, Guo F, Lian ZY, Jin XZ, Liu XH, Yin HC. Study on the epidemiological characteristics of fever accompanying by thrombocytopenia syndrome in Suizhou. Chinese Journal of Health Laboratory Technology. 2014; 24(01): 117-9.

370. Hu J, Li Z, Cai J, Liu D, Zhang X, Jiang R, et al. A Cluster of Bunyavirus-associated severe fever with thrombocytopenia syndrome cases in a coastal plain area in China, 2015: Identification of a previously unidentified endemic region for severe fever with thrombocytopenia Bunyavirus. Open Forum Infect Dis. 2019; 6(6): ofz209.

371. Hu XQ, Tian H, Ning B, Yu PB, Wei J, Sun YF, et al. Epidemiological investigation of the first case of severe fever with thrombocytopenia syndrome in Shaanxi province. Journal of Medical Pest Control. 2014; 30(09): 1041-2.

372. Hu XQ, Zeng LX, Tian H, Yu PB, Sun YF, Hu K, et al. Investigation of new bunyavirus infection in Baoji city. Journal of Xi'an Jiaotong University (Medical Sciences). 2015; 36(04): 571-3.

373. Huang WZ, Yu XH, Wu HZ, Zhang L, Miu ZP, Lv HK. Investigation and analysis on the transmission of severe fever with thrombocytopenia syndrome via contact with patient. Chin J Vector Biol Control. 2015; 26(02): 172-5.

374. Huang X, Zhang Z, Jin G, Wang X, Tan C, Yin H, et al. Presence of antibodies against severe fever with thrombocytopenia syndrome virus in non-endemic areas of China. Jpn J Infect Dis. 2017; 70(3): 248-51.

375. Huang XY, Du YH, Wang HF, You AG, Li Y, Su J, et al. Prevalence of severe fever with thrombocytopenia syndrome virus in animals in Henan province, China. Infect Dis Poverty. 2019; 8(1): 56.

376. Jiang XL, Wang XJ, Li JD, Ding SJ, Zhang QF, Qu J, et al. Isolation, identification and characterization of SFTS Bunyavirus from ticks collected on the surface of domestic animals. Chin J Virol. 2012; 28(03): 252-7.

377. Li J, Kelly P, Guo W, Zhang J, Yang Y, Liu W, et al. Molecular detection of Rickettsia, Hepatozoon, Ehrlichia and SFTSV in goat ticks. Vet Parasitol Reg Stud Reports. 2020; 20: 100407.

378. Li JX, Zhao X, Jin GX, Jin GJ, Ma GJ, Jin JN, et al. Investigation of free ticks carrying CRT and compound infection with SFTSV in Yanbian area of Jilin province. Chinese Journal of Disease Control & Prevention. 2019; 23(05): 561-6+72.

379. Li L, Zhang J, Zhong J, Zheng XS, Zhan L. An investigation on the serum epidemiology of the new-Bunyavirus among the blood-donors in Guiyang city. Int J Lab Med. 2017; 38(16): 2246-8.

380. Li Y, Lan YQ, Liu FM, Pan XM, Yu Y, Sun JM. Investigation of the first case of severe fever with thrombocytopenia syndrome caused by new Bunyavirus infection in Lishui city. Preventive<U+00A0>Medicine. 2018; 30(10): 1031-3+6.

381. Li Z, Bao C, Hu J, Liu W, Wang X, Zhang L, et al. Ecology of the tick-borne phlebovirus causing severe fever with thrombocytopenia syndrome in an endemic area of China. PLoS Negl Trop Dis. 2016; 10(4): e0004574.

382. Li Z, Hu J, Bao C, Li P, Qi X, Qin Y, et al. Seroprevalence of antibodies against SFTS virus infection in farmers and animals, Jiangsu, China. J Clin Virol. 2014; 60(3): 185-9.

383. Liang S, Bao C, Zhou M, Hu J, Tang F, Guo X, et al. Seroprevalence and risk factors for severe fever with thrombocytopenia syndrome virus infection in Jiangsu province, China, 2011. Am J Trop Med Hyg. 2014; 90(2): 256-9.

384. Lin TL, Ou SC, Maeda K, Shimoda H, Chan JP, Tu WC, et al. The first discovery of severe fever with thrombocytopenia syndrome virus in Taiwan. Emerg Microbes Infect. 2020; 9(1): 148-51.

385. Liu H, Li Z, Wang Z, He B, Wang S, Wei F, et al. The first molecular evidence of severe fever with thrombocytopenia syndrome virus in ticks in Jilin, northeastern China. Ticks Tick Borne Dis. 2016; 7(6): 1280-3.

386. Liu JW, Wen HL, Fang LZ, Zhang ZT, He ST, Xue ZF, et al. Prevalence of SFTSV among Asian house shrews and rodents, China, January-August 2013. Emerg Infect Dis. 2014; 20(12): 2126-8.

387. Liu Y, Zhang J, Wang B, Tian J, Mao LL, Sun YW, et al. Epidemic characteristics and biological features of severe fever with thrombocytopenia syndrome bunyavirus (SFTSV) found in Liaoning province. Chin J Publ Heal. 2013; 29(05): 721-3.

388. Liu ZD, Yin HY, Jiang YF, Jiang SX. Survey on antibody of new bunyavirus among healthy people and domestic animals Laizhou city 2011. Preventive Medicine Tribune. 2014; 20(04): 274-6.

389. Luo LM, Zhao L, Wen HL, Zhang ZT, Liu JW, Fang LZ, et al. Haemaphysalis longicornis ticks as reservoir and vector of severe fever with thrombocytopenia syndrome virus in China. Emerg Infect Dis. 2015; 21(10): 1770-6.

390. Mao L, Deng B, Liang Y, Liu Y, Wang Z, Zhang J, et al. Epidemiological and genetic investigation of a cluster of cases of severe fever with thrombocytopenia syndrome bunyavirus. BMC Infect Dis. 2020; 20(1): 346.

391. Meng K, Sun W, Cheng Z, Guo H, Liu J, Chai T. First detection of severe fever with thrombocytopenia syndrome virus in the tick species Haemaphysalis concinna in Shandong province, China. Parasitol Res. 2015; 114(12): 4703-7.

392. Ni H, Yang F, Li Y, Liu W, Jiao S, Li Z, et al. Apodemus agrarius is a potential natural host of severe fever with thrombocytopenia syndrome (SFTS)-causing novel bunyavirus. J Clin Virol. 2015; 71: 82-8.

393. Niu G, Li J, Liang M, Jiang X, Jiang M, Yin H, et al. Severe fever with thrombocytopenia syndrome virus among domesticated animals, China. Emerg Infect Dis. 2013; 19(5): 756-63.

394. Peng HY, Cui L, Cui LB, Hu JL, Bao CJ, Li Y, et al. Virus-carrying status of animals with fever and thrombocytopenia syndrome in some regions of Jiangsu province during 2012. Acta Med Univ Sci Technol Huazhong. 2015; 44(02): 235-8.

395. Qi R, Qin XR, Wang L, Han HJ, Cui F, Yu H, et al. Severe fever with thrombocytopenia syndrome can masquerade as hemorrhagic fever with renal syndrome. PLoS Negl Trop Dis. 2019; 13(3): e0007308.

396. Shen W, Lin H, Weng J, Hu Y, Liu Y, Li J, et al. Seroprevalence of severe fever with thrombocytopenia syndrome virus antibodies among inhabitants of Dachen Island, eastern China. Ticks Tick Borne Dis. 2019; 10(3): 647-50.

397. Song LH, Geng YZ, Pang W, Wu W, Liu DH, Zhou D, et al. Bunyaviruss detected from ticks positive for nucleic acid. China Tropical Medicine. 2012; 12(12): 1547-8.

398. Sun J, Tang Y, Ling F, Chang Y, Ye X, Shi W, et al. Genetic susceptibility is one of the determinants for severe fever with thrombocytopenia syndrome virus infection and fatal outcome: An epidemiological investigation. PLoS One. 2015; 10(7): e0132968.

399. Sun JM, Zhang YJ, Gong ZY, Zhang L, Lv HK, Lin JF, et al. Seroprevalence of severe fever with thrombocytopenia syndrome virus in southeastern China and analysis of risk factors. Epidemiol Infect. 2015; 143(4): 851-6.

400. Tan WW, Huang P, Hu JL, Zhou JG, Zhang HJ, Yu RB. Results of surveillance of severe fever with thrombocytopenia syndrome bunyavirus in Yixing. China Tropical Medicine. 2015; 15(03): 359-60.

401. Tian H, Yu P, Chowell G, Li S, Wei J, Tian H, et al. Severe fever with thrombocytopenia syndrome virus in humans, domesticated animals, ticks, and mosquitoes, Shaanxi province, China. Am J Trop Med Hyg. 2017; 96(6): 1346-9.

402. Wang GS, Wang JB, Tian FL, Zhang HJ, Yin FF, Xu C, et al. Severe fever with thrombocytopenia syndrome virus infection in minks in China. Vector Borne Zoonotic Dis. 2017; 17(8): 596-8.

403. Wang LY, Yang ZD, Sun Y, Zhuang L, Tang F, Cui N, et al. Survey and genetic analysis of severe fever with thrombocytopenia syndrome virus from Haemaphysalis longicornis. Journal of Parasitic Biology. 2014; 9(07): 629-32.

404. Wang QK, Ge HM, Hu JL, Zhang ZY, Wang YP, Jiao YJ, et al. Surveillance of vectors and host animals of severe fever with thrombocytopenia syndrome virus in Donghai, China in 2010-2011. Chin J Vector Biol Control. 2013; 24(04): 313-6.

405. Wang QK, Ge HM, Li ZF, Dan YF, Cui L, Wang YP. Vector research of severe fever with thrombocytopenia syndrome virus in gamasid mites and chigger mites. Chin J Vector Biol Control. 2012; 23(05): 452-4.

406. Wang S, Li J, Niu G, Wang X, Ding S, Jiang X, et al. SFTS virus in ticks in an endemic area of China. Am J Trop Med Hyg. 2015; 92(4): 684-9.

407. Wei J, Li S, Dong JH, Tian H, Chowell G, Tian HY, et al. The first human infection with severe fever with thrombocytopenia syndrome virus in Shaanxi province, China. Int J Infect Dis. 2015; 35: 37-9.

408. Xing X, Guan X, Liu L, Zhan J, Jiang H, Liu L, et al. Natural transmission model for severe fever with thrombocytopenia syndrome Bunyavirus in villages of Hubei province, China. Medicine (Baltimore). 2016; 95(4): e2533.

409. Xing Y, Liu J, Niu G, Ding S, Gong L, Liu J. [Epidemiological investigation of predominance tick and the infectious status of severe fever thrombocytopenia syndrome virus in Penglai and Laizhou counties, Shandong province]. Chinese Journal of Preventive Medicine. 2015; 49(11): 993-7.

410. Xing YF, Liu JY, Niu GY, Qin YJ, Ding SJ, Gong LF. Epidemiological investigation on dominant tick species and infection status of novel Bunyavirus in ticks in rural hilly areas of Yantai city. Industrial Competitiveness and Innovation Drive-Proceedings of the 2014 Shandong Association for Science and Technology Annual Conference. Zibo, China, **2014**:6.

411. Xiong JF, Zhan JB, Tan LF, Yue JL, Peng QH, Yao X. Survey on ticks and host animals of severe fever with thrombocytopenia syndrome virus in Huanggang, Hubei province. Chin J Vector Biol Control. 2016; 27(05): 504-5.

412. Xu B, Liu L, Huang X, Ma H, Zhang Y, Du Y, et al. Metagenomic analysis of fever, thrombocytopenia and leukopenia syndrome (FTLS) in Henan province, China: discovery of a new bunyavirus. PLoS Pathog. 2011; 7(11): e1002369.

413. Xu PP, Lv Y, Geng XY, Zhang LM, Qin W, Hu JY, et al. Seroepidemiology of severe fever with thrombocytopenia syndrome virus, Liu'an. Modern Preventive Medicine. 2015; 42(11): 1948-50.

414. Xu XH, Li JP, Yin HY, Liu GS, Jiang SX, Wang WQ, et al. Study on the classification of ticks and etiology of novel Bunyavirus inticks, Laizhou city. Preventive Medicine Tribune. 2015; 21(01): 1-3.

415. Xu ZP, Qian YH, Shi C, Tan WW, Zhou JG, Yu RB. Analysis on new bunia virus infections in Wuxi city. Chinese Journal of Disease Control & Prevention. 2014; 18(03): 239-42.

416. Ye L, Ren Y, Wang ZF, Wang CW, Li SB. Surveillance and analysis of new Bunia virus infections in Zhoushan island area during 2011-2012. Chin J Vector Biol Control. 2013; 24(05): 429-31.

417. Yi B, Gao L, Kong ZF, Chen Y, Li YD. The results of the epidemiological surveillance of severe fever with thrombocytopenia syndrome in Ningbo city. Zhejiang Journal of Preventive Medicine. 2015; 27(02): 127-30.

418. Yin XX, Li YG, Xin YQ, Jiang ZH, Wang B, Liang Z, et al. Seroepidemiologic distribution for infection of severe fever with thrombocytopenia syndrome bunyavirus among domestic animals in Pulandian district of Dalian city, 2017. Chin J Publ Heal. 2019; 35(02): 243-5.

419. Yu L, Yu P, Xiao B, Hou J, Chen YF. Fever with thrombocytopenia syndrome bunya virus surveillance analysis in Dalian during 2011-2013. Journal of Medical Pest Control. 2015; 31(04): 422-4+7.

420. Yu XJ, Liang MF, Zhang SY, Liu Y, Li JD, Sun YL, et al. Fever with thrombocytopenia associated with a novel bunyavirus in China. N Engl J Med. 2011; 364(16): 1523-32.

421. Zeng P, Ma L, Gao Z, Wang J, Liu J, Huang X, et al. A study of seroprevalence and rates of asymptomatic viremia of severe fever with thrombocytopenia syndrome virus among Chinese blood donors. Transfusion. 2015; 55(5): 965-71.

422. Zhan JB, Huo XX, Guan XH, Jiang YZ, Xu JQ, Li GM, et al. Analysis on antibody levels against severe fever with thrombocytopenia syndrome bunyavirus among healthy population in Hubei province. Chinese Journal of Health Laboratory Technology. 2013; 23(04): 992-3.

423. Zhang H, Sun Y, Jiang H, Huo X. Prevalence of severe febrile and thrombocytopenic syndrome virus, Anaplasma spp. and Babesia microti in hard ticks (Acari: Ixodidae) from Jiaodong peninsula, Shandong province. Vector Borne Zoonotic Dis. 2017; 17(2): 134-40.

424. Zhang HL, Sun Y, Wang JJ. Investigation on foci severe fever with thrombocytopenia syndrome and human granulocytic anaplasmosis in Lujiang county. Chinese Journal of Disease Control & Prevention. 2015; 19(03): 273-6.

425. Zhang L, Sun J, Yan J, Lv H, Chai C, Sun Y, et al. Antibodies against severe fever with thrombocytopenia syndrome virus in healthy persons, China, 2013. Emerg Infect Dis. 2014; 20(8): 1355-7.

426. Zhang QZ, Dou HJ, Guo JL, Zhao HD, Zhou W, Li XW, et al. Investigation and analysis of tick distribution and tick vector in Yunan district. Modern Preventive Medicine. 2015; 42(19): 3578-80.

427. Zhang QZ, Luo HL, Li XW, Dong ZM. Survey on ticks and pathogens in the serum of its host in Xinyang city, Henan province. Journal of Henan Normal University(Natural Science Edition). 2016; 44(05): 126-30.

428. Zhang WS, Zeng XY, Zhou MH, Jiao YJ, Wen T, Guo XL, et al. Seroepidemiology of severe fever with thrombocytopenia syndrome bunyavirus in Jiangsu province. Dis Surveill. 2011; 26(09): 676-8.

429. Zhang YZ, Zhou DJ, Xiong Y, Chen XP, He YW, Sun Q, et al. Hemorrhagic fever caused by a novel tick-borne Bunyavirus in Huaiyangshan, China. Chin J Epidemiol. 2011; 32(3): 209-20.

430. Zhao L, Zhai S, Wen H, Cui F, Chi Y, Wang L, et al. Severe fever with thrombocytopenia syndrome virus, Shandong province, China. Emerg Infect Dis. 2012; 18(6): 963-5.

431. Zhou SQ, Ning FJ, Meng SX, Ji JH, Liu SS, Ding SJ, et al. Serological investigation of the new virus Bunia infection among healthy population in Penglai city. Modern Preventive Medicine. 2015; 42(03): 476-8.

432. Zhou YJ, Zhu SW, Jiang NZ, Lin H. Serological investigation of the new bunyavirus infection among voluntary blood donors in Jiangning of Nanjing. Chinese Journal of Hygienic Insecticides & Equipments. 2019; 25(04): 357-9.

433. Zhuang L, Du J, Cui XM, Li H, Tang F, Zhang PH, et al. Identification of tick-borne pathogen diversity by metagenomic analysis in Haemaphysalis longicornis from Xinyang, China. Infect Dis Poverty. 2018; 7(1): 45.

434. Shi M, Lin XD, Tian JH, Chen LJ, Chen X, Li CX, et al. Redefining the invertebrate RNA virosphere. Nature. 2016; 540(7634): 539-43.

435. Shen S, Duan X, Wang B, Zhu L, Zhang Y, Zhang J, et al. A novel tick-borne phlebovirus, closely related to severe fever with thrombocytopenia syndrome virus and Heartland virus, is a potential pathogen. Emerg Microbes Infect. 2018; 7(1): 95.

436. Shao L, Pang Z, Fu H, Chang R, Lin Z, Lv A, et al. Identification of recently identified tick-borne viruses (Dabieshan tick virus and SFTSV) by metagenomic analysis in ticks from Shandong province, China. J Infect. 2020; 81(6): 973-8.

437. Zhu C, He T, Wu T, Ai L, Hu D, Yang X, et al. Distribution and phylogenetic analysis of Dabieshan tick virus in ticks collected from Zhoushan, China. J Vet Med Sci. 2020; 82(8): 1226-30.

438. Liu W, Sun FJ, Tong YG, Zhang SQ, Cao WC. Rift Valley fever virus imported into China from Angola. Lancet Infect Dis. 2016; 16(11): 1226.

439. Wang J, Fan N, Fu S, Cheng J, Wu B, Xu Z, et al. Isolation and characterization of Wuxiang virus from sandflies collected in Yangquan county, Shanxi province, China. Vector Borne Zoonotic Dis. 2021; 21(6): 446-57.

440. Wang J, Fu S, Xu Z, Cheng J, Shi M, Fan N, et al. Emerging sandfly-borne phlebovirus in China. Emerg Infect Dis. 2020; 26(10): 2435-8.

441. Wang Q, Fu S, Cheng J, Xu X, Wang J, Wu B, et al. Re-isolation of Wuxiang virus from wild sandflies collected from Yangquan county, China. Virol Sin. 2021:; 1-10.

442. Xu X, Cheng J, Fu S, Wang Q, Wang J, Lu X, et al. Wuxiang virus is a virus circulated naturally in Wuxiang county, China. Vector Borne Zoonotic Dis. 2021; 21(4): 289-300.

443. Abdelgadir DM, Bashab HM, Elhadi Mohamed RA, Abuelmaali SA. Risk factor analysis for outbreak of Rift Valley fever in Khartoum State of Sudan. Journal of Entomological Science. 2010; 45(3): 239-51.

444. Cooke FJ, Shapiro DS. Rift valley fever in Sudan. Int J Infect Dis. 2008; 12(1): 1-.

445. McIntosh B, Jupp, PG, Dos Santos, I., Rowe A. Field and laboratory evidence implicating Culex zombaensis and Aedes circumluteolus as vectors of Rift Valley fever virus in coastal South Africa. South African Journal of Science. 1983; 79(2): 61-4.

446. Mohamed RAEH, Mohamed N, Aleanizy FS, Alqahtani FY, Al Khalaf A, Al-Keridis LA. Investigation of hemorrhagic fever viruses inside wild populations of ticks: One of the pioneer studies in Saudi Arabia. Asian Pac J Trop Dis. 2017; 7(5): 299-303.

447. Salim RW, Khairalla KM, Eljamal AA, Karrar A, Aradaib I. A single tube RT-PCR amplification for detection of rift valley fever virus. Research Journal of Medical Sciences. 2010; 4(3): 146-51.

448. Sutherland LJ, Muiruri S, Muchiri EM, Gray LR, Zimmerman PA, Hise AG, et al. Prevalence and fine-scale distribution of Rift Valley fever virus and West Nile virus in mosquitoes during a Rift Valley fever outbreak in northeastern province, Kenya. Am J Trop Med Hyg. 2009; 81(5): 13-.

449. Wiwanitkit V. Emerging Rift Valley fever in China: What should be known? Asian Pac J Trop Biomed. 2016; 6(9): 727-9.

450. Munyua P, Murithi RM, Wainwright S, Githinji J, Hightower A, Mutonga D, et al. Rift Valley fever outbreak in livestock in Kenya, 2006-2007. Am J Trop Med Hyg. 2010; 83(2 Suppl): 58-64.

451. Tshilenge GM, Dundon WG, De Nardi M, Mulumba Mfumu LK, Rweyemamu M, Kayembe-Ntumba JM, et al. Seroprevalence of Rift Valley fever virus in cattle in the Democratic Republic of the Congo. Trop Anim Health Prod. 2019; 51(3): 537-43.

452. Fabiansen C, Thybo S. [Hemorrhagic Rift Valley fever]. Ugeskr Laeger. 2007; 169(26): 2537-8.

453. J. CF, S. SD. Rift Valley Fever in East Africa. Int J Infect Dis. 2007; 11(4): 287-8.

454. Abdel-Wahab KS, El Baz LM, El-Tayeb EM, Omar H, Ossman MA, Yasin W. Rift Valley fever virus infections in Egypt: pathological and virological findings in man. Trans R Soc Trop Med Hyg. 1978; 72(4): 392-6.

455. Abdo-Salem S, Gerbier G, Bonnet P, Al-Qadasi M, Tran A, Thiry E, et al. Descriptive and spatial epidemiology of Rift Valley fever outbreak in Yemen 2000-2001. Ann N Y Acad Sci. 2006; 1081: 240-2.

456. Ahmad K. More deaths from Rift Valley fever in Saudi Arabia and Yemen. Lancet. 2000; 356(9239): 1422.

457. Altmann M, Nahapetyan K, Asghar H. Identifying hotspots of viral haemorrhagic fevers in the Eastern Mediterranean region: perspectives for the emerging and dangerous pathogens laboratory network. East Mediterr Health J. 2019; 24(11): 1049-57.

458. Andriamandimby SF, Randrianarivo-Solofoniaina AE, Jeanmaire EM, Ravololomanana L, Razafimanantsoa LT, Rakotojoelinandrasana T, et al. Rift Valley fever during rainy seasons, Madagascar, 2008 and 2009. Emerg Infect Dis. 2010; 16(6): 963-70.

459. Anyamba A, Chretien JP, Small J, Tucker CJ, Formenty PB, Richardson JH, et al. Prediction of a Rift Valley fever outbreak. Proc Natl Acad Sci USA. 2009; 106(3): 955-9.

460. Aradaib IE, Erickson BR, Elageb RM, Khristova ML, Carroll SA, Elkhidir IM, et al. Rift Valley fever, Sudan, 2007 and 2010. Emerg Infect Dis. 2013; 19(2): 246-53.

461. Archer BN, Thomas J, Weyer J, Cengimbo A, Landoh DE, Jacobs C, et al. Epidemiologic investigations into outbreaks of Rift Valley fever in humans, South Africa, 2008-2011. Emerg Infect Dis. 2013; 19(12): 1918-25.

462. Archer BN, Weyer J, Paweska J, Nkosi D, Leman P, Tint KS, et al. Outbreak of Rift Valley fever affecting veterinarians and farmers in South Africa, 2008. S Afr Med J. 2011; 101(4): 263-6.

463. Ba Y, Sall AA, Diallo D, Mondo M, Girault L, Dia I, et al. Re-emergence of Rift Valley fever virus in Barkedji (Senegal, West Africa) in 2002-2003: identification of new vectors and epidemiological implications. J Am Mosq Control Assoc. 2012; 28(3): 170-8.

464. Baudin M, Jumaa AM, Jomma HJE, Karsany MS, Bucht G, N<U+00E4>slund J, et al. Association of Rift Valley fever virus infection with miscarriage in Sudanese women: a cross-sectional study. Lancet Glob Health. 2016; 4(11): e864-e71.

465. Bird BH, Githinji JW, Macharia JM, Kasiiti JL, Muriithi RM, Gacheru SG, et al. Multiple virus lineages sharing recent common ancestry were associated with a large Rift Valley fever outbreak among livestock in Kenya during 2006-2007. J Virol. 2008; 82(22): 11152-66.

466. Bob NS, Ba H, Fall G, Ishagh E, Diallo MY, Sow A, et al. Detection of the northeastern African Rift Valley fever virus lineage during the 2015 outbreak in Mauritania. Open Forum Infect Dis. 2017; 4(2): ofx087.

467. Boushab BM, Fall-Malick FZ, Ould Baba SE, Ould Salem ML, Belizaire MR, Ledib H, et al. Severe human illness caused by Rift Valley fever virus in Mauritania, 2015. Open Forum Infect Dis. 2016; 3(4): ofw200.

468. Boushab MB, Savadogo M, Sow MS, Fall-Malick FZ, Seydi M. [Severe hemorrhagic form of Rift Valley fever in Mauritania]. Bull Soc Pathol Exot. 2015; 108(2): 102-6.

469. Butenko AM. [Arbovirus circulation in the Republic of Guinea]. Med Parazitol (Mosk). 1996; (2): 40-5.

470. C¨ºtre-Sossah C, Zeller H, Grandadam M, Caro V, Pettinelli F, Bouloy M, et al. Genome analysis of Rift Valley fever virus, Mayotte. Emerg Infect Dis. 2012; 18(6): 969-71.

471. Capobianco Dondona A, Aschenborn O, Pinoni C, Di Gialleonardo L, Maseke A, Bortone G, et al. Rift Valley fever virus among wild ruminants, Etosha National Park, Namibia, 2011. Emerg Infect Dis. 2016; 22(1): 128-30.

472. Carroll SA, Reynes JM, Khristova ML, Andriamandimby SF, Rollin PE, Nichol ST. Genetic evidence for Rift Valley fever outbreaks in Madagascar resulting from virus introductions from the east African mainland rather than enzootic maintenance. J Virol. 2011; 85(13): 6162-7.

473. Chengula AA, Kasanga CJ, Mdegela RH, Sallu R, Yongolo M. Molecular detection of Rift Valley fever virus in serum samples from selected areas of Tanzania. Trop Anim Health Prod. 2014; 46(4): 629-34.

474. De St Maurice A, Harmon J, Nyakarahuka L, Balinandi S, Tumusiime A, Kyondo J, et al. Rift valley fever viral load correlates with the human inflammatory response and coagulation pathway abnormalities in humans with hemorrhagic manifestations. PLoS Negl Trop Dis. 2018; 12(5): e0006460.

475. De St Maurice A, Nyakarahuka L, Purpura L, Ervin E, Tumusiime A, Balinandi S, et al. Notes from the field: Rift Valley fever response - Kabale district, Uganda, March 2016. MMWR Morb Mortal Wkly Rep. 2016; 65(43): 1200-1.

476. Diallo M, Lochouarn L, Ba K, Sall AA, Mondo M, Girault L, et al. First isolation of the Rift Valley fever virus from Culex poicilipes (Diptera: Culicidae) in nature. Am J Trop Med Hyg. 2000; 62(6): 702-4.

477. Diallo M, Nabeth P, Ba K, Sall AA, Ba Y, Mondo M, et al. Mosquito vectors of the 1998-1999 outbreak of Rift Valley fever and other arboviruses (Bagaza, Sanar, Wesselsbron and West Nile) in Mauritania and Senegal. Med Vet Entomol. 2005; 19(2): 119-26.

478. Durand JP, Bouloy M, Richecoeur L, Peyrefitte CN, Tolou H. Rift Valley fever virus infection among French troops in Chad. Emerg Infect Dis. 2003; 9(6): 751-2.

479. El Imam M, El Sabiq M, Omran M, Abdalkareem A, El Gaili Mohamed MA, Elbashir A, et al. Acute renal failure associated with the Rift Valley fever: a single center study. Saudi J Kidney Dis Transpl. 2009; 20(6): 1047-52.

480. El Mamy AB, Baba MO, Barry Y, Isselmou K, Dia ML, El Kory MO, et al. Unexpected Rift Valley fever outbreak, northern Mauritania. Emerg Infect Dis. 2011; 17(10): 1894-6.

481. El Mamy AB, Lo MM, Thiongane Y, Diop M, Isselmou K, Doumbia B, et al. Comprehensive phylogenetic reconstructions of Rift Valley fever virus: the 2010 northern Mauritania outbreak in the Camelus dromedarius species. Vector Borne Zoonotic Dis. 2014; 14(12): 856-61.

482. Fafetine JM, Coetzee P, Mubemba B, Nhambirre O, Neves L, Coetzer JA, et al. Rift Valley fever outbreak in livestock, Mozambique, 2014. Emerg Infect Dis. 2016; 22(12): 2165-7.

483. Faye O, Ba H, Ba Y, Freire CC, Faye O, Ndiaye O, et al. Reemergence of Rift Valley fever, Mauritania, 2010. Emerg Infect Dis. 2014; 20(2): 300-3.

484. Faye O, Diallo M, Diop D, Bezeid OE, Ba H, Niang M, et al. Rift Valley fever outbreak with East-Central African virus lineage in Mauritania, 2003. Emerg Infect Dis. 2007; 13(7): 1016-23.

485. Fontenille D, Traore-Lamizana M, Diallo M, Thonnon J, Digoutte JP, Zeller HG. New vectors of Rift Valley fever in West Africa. Emerg Infect Dis. 1998; 4(2): 289-93.

486. Fu X, Wang L, Fang B, Ma R, Zheng Y, Huang S, et al. Import of Rift Valley fever to China: a potential new threat. Virol Sin. 2016; 31(5): 454-6.

487. Gear J, De Meillon B, Le Roux AF, Kofsky R, Innes RR, Steyn JJ, et al. Rift valley fever in South Africa; a study of the 1953 outbreak in the Orange Free state, with special reference to the vectors and possible reservoir hosts. S Afr Med J. 1955; 29(22): 514-8.

488. Georges TM, Justin M, Victor M, Marie KJ, Mark R, L¨¦opold MMK. Seroprevalence and virus activity of Rift Valley fever in cattle in eastern region of Democratic Republic of the Congo. J Vet Med. 2018; 2018: 4956378.

489. Grolla A, Mehedi M, Lindsay R, Bosio C, Duse A, Feldmann H. Enhanced detection of Rift Valley fever virus using molecular assays on whole blood samples. J Clin Virol. 2012; 54(4): 313-7.

490. Guillebaud J, Bernardson B, Randriambolamanantsoa TH, Randrianasolo L, Randriamampionona JL, Marino CA, et al. Study on causes of fever in primary healthcare center uncovers pathogens of public health concern in Madagascar. PLoS Negl Trop Dis. 2018; 12(7): e0006642.

491. Hanafi HA, Fryauff DJ, Saad MD, Soliman AK, Mohareb EW, Medhat I, et al. Virus isolations and high population density implicate Culex antennatus (Becker) (Diptera: Culicidae) as a vector of Rift Valley fever virus during an outbreak in the Nile Delta of Egypt. Acta Trop. 2011; 119(2-3): 119-24.

492. Haneche F, Leparc-Goffart I, Simon F, Hentzien M, Martinez-Pourcher V, Caumes E, et al. Rift Valley fever in kidney transplant recipient returning from Mali with viral RNA detected in semen up to four months from symptom onset, France, autumn 2015. Euro Surveill. 2016; 21: (18).

493. Henderson BE, Mccrae AW, Kirya BG, Ssenkubuge Y, Sempala SD. Arbovirus epizootics involving man, mosquitoes and vertebrates at Lunyo, Uganda 1968. Ann Trop Med Parasitol. 1972; 66(3): 343-55.

494. Hightower A, Kinkade C, Nguku PM, Anyangu A, Mutonga D, Omolo J, et al. Relationship of climate, geography, and geology to the incidence of Rift Valley fever in Kenya during the 2006-2007 outbreak. Am J Trop Med Hyg. 2012; 86(2): 373-80.

495. Hoogstraal H, Meegan JM, Khalil GM, Adham FK. The Rift Valley fever epizootic in Egypt 1977-78. 2. Ecological and entomological studies. Trans R Soc Trop Med Hyg. 1979; 73(6): 624-9.

496. Imam IZ, Darwish MA, El-Karamany R. An epidemic of Rift Valley fever in Egypt. 1. Diagnosis of Rift Valley fever in man. Bull World Health Organ. 1979; 57(3): 437-9.

497. Imam IZ, El-Karamany R, Darwish MA. An epidemic of Rift Valley fever in Egypt. 2. Isolation of the virus from animals. Bull World Health Organ. 1979; 57(3): 441-3.

498. J<U+00E4>ckel S, Eiden M, El Mamy BO, Isselmou K, Vina-Rodriguez A, Doumbia B, et al. Molecular and serological studies on the Rift Valley fever outbreak in Mauritania in 2010. Transbound Emerg Dis. 2013; 60 Suppl 2: 31-9.

499. Jost CC, Nzietchueng S, Kihu S, Bett B, Njogu G, Swai ES, et al. Epidemiological assessment of the Rift Valley fever outbreak in Kenya and Tanzania in 2006 and 2007. Am J Trop Med Hyg. 2010; 83(2 Suppl): 65-72.

500. Jouan A, Le Guenno B, Digoutte JP, Philippe B, Riou O, Adam F. An RVF epidemic in southern Mauritania. Ann Inst Pasteur Virol. 1988; 139(3): 307-8.

501. Jupp PG, Kemp A, Grobbelaar A, Lema P, Burt FJ, Alahmed AM, et al. The 2000 epidemic of Rift Valley fever in Saudi Arabia: mosquito vector studies. Med Vet Entomol. 2002; 16(3): 245-52.

502. Kifaro EG, Nkangaga J, Joshua G, Sallu R, Yongolo M, Dautu G, et al. Epidemiological study of Rift Valley fever virus in Kigoma, Tanzania. Onderstepoort J Vet Res. 2014; 81(2): E1-5.

503. Konstantinov OK, Diallo SM, Inapogi AP, Ba A, Kamara SK. [The mammals of Guinea as reservoirs and carriers of arboviruses]. Med Parazitol (Mosk). 2006; (1): 34-9.

504. Labeaud AD, Sutherland LJ, Muiruri S, Muchiri EM, Gray LR, Zimmerman PA, et al. Arbovirus prevalence in mosquitoes, Kenya. Emerg Infect Dis. 2011; 17(2): 233-41.

505. Lagare A, Fall G, Ibrahim A, Ousmane S, Sadio B, Abdoulaye M, et al. First occurrence of Rift Valley fever outbreak in Niger, 2016. Vet Med Sci. 2019; 5(1): 70-8.

506. Lee VH. Isolation of viruses from field populations of culicoides (Diptera: Ceratopogonidae) in Nigeria. J Med Entomol. 1979; 16(1): 76-9.

507. Lichoti JK, Kihara A, Oriko AA, Okutoyi LA, Wauna JO, Tchouassi DP, et al. Detection of Rift Valley fever virus interepidemic activity in some hotspot areas of kenya by sentinel animal surveillance, 2009-2012. Vet Med Int. 2014; 2014: 379010.

508. Linthicum KJ, Davies FG, Kairo A, Bailey CL. Rift Valley fever virus (family Bunyaviridae, genus Phlebovirus). Isolations from Diptera collected during an inter-epizootic period in Kenya. J Hyg (Lond). 1985; 95(1): 197-209.

509. Liu J, Sun Y, Shi W, Tan S, Pan Y, Cui S, et al. The first imported case of Rift Valley fever in China reveals a genetic reassortment of different viral lineages. Emerg Microbes Infect. 2017; 6(1): e4.

510. Lutomiah J, Omondi D, Masiga D, Mutai C, Mireji PO, Ongus J, et al. Blood meal analysis and virus detection in blood-fed mosquitoes collected during the 2006-2007 Rift Valley fever outbreak in Kenya. Vector Borne Zoonotic Dis. 2014; 14(9): 656-64.

511. Madani TA, Al-Mazrou YY, Al-Jeffri MH, Mishkhas AA, Al-Rabeah AM, Turkistani AM, et al. Rift Valley fever epidemic in Saudi Arabia: epidemiological, clinical, and laboratory characteristics. Clin Infect Dis. 2003; 37(8): 1084-92.

512. Mbanzulu KM, Wumba R, Mukendi JK, Zanga JK, Shija F, Bobanga TL, et al. Mosquito-borne viruses circulating in Kinshasa, Democratic Republic of the Congo. Int J Infect Dis. 2017; 57: 32-7.

513. Meegan JM, Khalil GM, Hoogstraal H, Adham FK. Experimental transmission and field isolation studies implicating Culex pipiens as a vector of Rift Valley fever virus in Egypt. Am J Trop Med Hyg. 1980; 29(6): 1405-10.

514. Miller BR, Godsey MS, Crabtree MB, Savage HM, Al-Mazrao Y, Al-Jeffri MH, et al. Isolation and genetic characterization of Rift Valley fever virus from Aedes vexans arabiensis, Kingdom of Saudi Arabia. Emerg Infect Dis. 2002; 8(12): 1492-4.

515. Mohamed M, Mosha F, Mghamba J, Zaki SR, Shieh WJ, Paweska J, et al. Epidemiologic and clinical aspects of a Rift Valley fever outbreak in humans in Tanzania, 2007. Am J Trop Med Hyg. 2010; 83(2 Suppl): 22-7.

516. Mohamed N, Magzoub M, Mohamed REH, Aleanizy FS, Alqahtani FY, Nour BYM, et al. Prevalence and identification of arthropod-transmitted viruses in Kassala state, Eastern Sudan. Libyan J Med. 2019; 14(1): 1564511.

517. Monaco F, Pinoni C, Cosseddu GM, Khaiseb S, Calistri P, Molini U, et al. Rift Valley fever in Namibia, 2010. Emerg Infect Dis. 2013; 19(12): 2025-7.

518. Mwaengo D, Lorenzo G, Iglesias J, Warigia M, Sang R, Bishop RP, et al. Detection and identification of Rift Valley fever virus in mosquito vectors by quantitative real-time PCR. Virus Res. 2012; 169(1): 137-43.

519. Nabeth P, Kane Y, Abdalahi MO, Diallo M, Ndiaye K, Ba K, et al. Rift Valley fever outbreak, Mauritania, 1998: seroepidemiologic, virologic, entomologic, and zoologic investigations. Emerg Infect Dis. 2001; 7(6): 1052-4.

520. Nderitu L, Lee JS, Omolo J, Omulo S, O'guinn ML, Hightower A, et al. Sequential Rift Valley fever outbreaks in eastern Africa caused by multiple lineages of the virus. J Infect Dis. 2011; 203(5): 655-65.

521. Ndiaye EH, Diallo D, Fall G, Ba Y, Faye O, Dia I, et al. Arboviruses isolated from the Barkedji mosquito-based surveillance system, 2012-2013. BMC Infect Dis. 2018; 18(1): 642.

522. Nguku PM, Sharif SK, Mutonga D, Amwayi S, Omolo J, Mohammed O, et al. An investigation of a major outbreak of Rift Valley fever in Kenya: 2006-2007. Am J Trop Med Hyg. 2010; 83(2 Suppl): 5-13.

523. Nicolas G, Durand B, Rakotoarimanana TT, Lacote S, Chevalier V, Marianneau P. A 3-year serological and virological cattle follow-up in Madagascar highlands suggests a non-classical transmission route of Rift Valley fever virus. Am J Trop Med Hyg. 2014; 90(2): 265-6.

524. Organization., Health. W. An outbreak of Rift Valley fever, eastern Africa, 1997-1998. Wkly Epidemiol Rec. 1998; 73(15): 105-9.

525. Organization., Health. W. Outbreak of Rift Valley fever, Yemen, August-October 2000. Wkly Epidemiol Rec. 2000; 75(48): 392-5.

526. Organization., Health. W. Outbreak news. Rift Valley fever, Kenya. Wkly Epidemiol Rec. 2007; 82(3): 17-8.

527. Organization., Health. W. Outbreak news. Rift Valley fever, United Republic of Tanzania. Wkly Epidemiol Rec. 2007; 82(14): 117-8.

528. Organization., Health. W. Outbreaks of Rift Valley fever in Kenya, Somalia and United Republic of Tanzania, December 2006-April 2007. Wkly Epidemiol Rec. 2007; 82(20): 169-78.

529. Organization., Health. W. Outbreak news. Rift Valley fever, Madagascar. Wkly Epidemiol Rec. 2008; 83(18): 157.

530. Organization., Health. W. Rift Valley fever, South Africa - update. Wkly Epidemiol Rec. 2010; 85(21): 185-6.

531. Organization., Health. W. Outbreak news. Rift Valley fever, Mauritania. Wkly Epidemiol Rec. 2012; 87(45): 438.

532. Prevention CfDCA. Rift Valley Fever-East Africa, 1997-1998. MMWR Morb Mortal Wkly Rep. 1998; 47(13): 261-4.

533. Prevention CfDCA. Outbreak of Rift Valley fever--Saudi Arabia, August-October, 2000. JAMA. 2000; 284(18): 2310-1.

534. Prevention CfDCA. Update: outbreak of Rift Valley fever--Saudi Arabia, August-November 2000. JAMA. 2000; 284(23): 2989-90.

535. Prevention CfDCA. Rift Valley fever outbreak-Kenya, November 2006-January 2007. MMWR Morb Mortal Wkly Rep. 2007; 56(4): 73-6.

536. Rakotoarivelo RA, Andrianasolo R, Razafimahefa SH, Randremandranto Razafimbelo NS, Randria MJ. [Severe presentations of Rift Valley fever in Madagascar]. Med Mal Infect. 2011; 41(6): 318-21.

537. Ratovonjato J, Olive MM, Tantely LM, Andrianaivolambo L, Tata E, Razainirina J, et al. Detection, isolation, and genetic characterization of Rift Valley fever virus from Anopheles (Anopheles) coustani, Anopheles (Anopheles) squamosus, and Culex (Culex) antennatus of the Haute Matsiatra region, Madagascar. Vector Borne Zoonotic Dis. 2011; 11(6): 753-9.

538. Raveloson NE, Ramorasata JC, Rasolofohanitrininosy R, Rakotoarivony ST, Andrianjatovo JJ, Sztark F. [Fatal haemorrhagic rift valley fever: a case at Madagascar]. Med Trop (Mars). 2010; 70(2): 177-9.

539. Sabirovic M, Raw L, Hall S, Elliott H, Coulson N. International disease surveillance. International disease monitoring, January to March 2008. Vet Rec. 2008; 162(22): 705-8.

540. Salem ML, Baba Sel W, Fall-Malick FZ, Boushab BM, Ghaber SM, Mokhtar A. [Severe hemorrhagic forms of Rift Valley fever: about 5 cases]. Pan Afr Med J. 2016; 24: 73.

541. Sall AA, De AZPM, Zeller HG, Digoutte JP, Thiongane Y, Bouloy M. Variability of the NS(S) protein among Rift Valley fever virus isolates. J Gen Virol. 1997; 78 ( Pt 11): 2853-8.

542. Sall AA, Zanotto PM, Sene OK, Zeller HG, Digoutte JP, Thiongane Y, et al. Genetic reassortment of Rift Valley fever virus in nature. J Virol. 1999; 73(10): 8196-200.

543. Sang R, Kioko E, Lutomiah J, Warigia M, Ochieng C, O'guinn M, et al. Rift Valley fever virus epidemic in Kenya, 2006/2007: the entomologic investigations. Am J Trop Med Hyg. 2010; 83(2 Suppl): 28-37.

544. Seufi AM, Galal FH. Role of Culex and Anopheles mosquito species as potential vectors of Rift Valley fever virus in Sudan outbreak, 2007. BMC Infect Dis. 2010; 10: 65.

545. Shraim MA, Eid R, Radad K, Saeed N. Ultrastructural pathology of human liver in Rift Valley fever. BMJ Case Rep. 2016; 2016.

546. Sissoko D, Giry C, Gabrie P, Tarantola A, Pettinelli F, Collet L, et al. Rift Valley fever, Mayotte, 2007-2008. Emerg Infect Dis. 2009; 15(4): 568-70.

547. Soumar¨¦ PO, Freire CC, Faye O, Diallo M, De Oliveira JV, Zanotto PM, et al. Phylogeography of Rift Valley fever virus in Africa reveals multiple introductions in Senegal and Mauritania. PLoS One. 2012; 7(4): e35216.

548. Sow A, Faye O, Ba Y, Ba H, Diallo D, Faye O, et al. Rift Valley fever outbreak, southern Mauritania, 2012. Emerg Infect Dis. 2014; 20(2): 296-9.

549. Sow A, Faye O, Ba Y, Diallo D, Fall G, Faye O, et al. Widespread Rift Valley fever emergence in Senegal in 2013-2014. Open Forum Infect Dis. 2016; 3(3): ofw149.

550. Sow A, Faye O, Faye O, Diallo D, Sadio BD, Weaver SC, et al. Rift Valley fever in Kedougou, southeastern Senegal, 2012. Emerg Infect Dis. 2014; 20(3): 504-6.

551. Sow A, Loucoubar C, Diallo D, Faye O, Ndiaye Y, Senghor CS, et al. Concurrent malaria and arbovirus infections in Kedougou, southeastern Senegal. Malar J. 2016; 15: 47.

552. Subudhi S, Dakouo M, Sloan A, Stein DR, Grolla A, Jones S, et al. Seroprevalence of Rift Valley fever virus antibodies in cattle in Mali, 2005-2014. Am J Trop Med Hyg. 2018; 98(3): 872-4.

553. Taha HA, Shoman SA, Alhadlag NM. Molecular and serological survey of some haemoprotozoan, rickettsial and viral diseases of small ruminants from Al-Madinah Al Munawarah, KSA. Trop Biomed. 2015; 32(3): 511-23.

554. Thiongane Y, Thonnon J, Zeller H, Lo MM, Faty A, Diagne F, et al. [Recent data on Rift Valley fever epidemiology in Senegal]. Dakar Med. 1996: 1-6.

555. Traor¨¦-Lamizana M, Fontenille D, Diallo M, Ba Y, Zeller HG, Mondo M, et al. Arbovirus surveillance from 1990 to 1995 in the Barkedji area (Ferlo) of Senegal, a possible natural focus of Rift Valley fever virus. J Med Entomol. 2001; 38(4): 480-92.

556. Woods CW, Karpati AM, Grein T, Mccarthy N, Gaturuku P, Muchiri E, et al. An outbreak of Rift Valley fever in northeastern Kenya, 1997-98. Emerg Infect Dis. 2002; 8(2): 138-44.

557. Youssef BZ. Application of reverse transcriptase-polymerase chain reaction for detection of Rift Valley fever viral antigen from mosquito. J Egypt Public Health Assoc. 2001; 76(3-4): 297-308.

558. Youssef BZ, Donia HA. The potential role of rattus rattus in enzootic cycle of Rift Valley fever in Egypt 2-application of reverse transcriptase polymerase chain reaction (RT-PCR) in blood samples of Rattus rattus. J Egypt Public Health Assoc. 2002; 77(1-2): 133-41.

559. Youssouf H, Subiros M, Dennetiere G, Collet L, Dommergues L, Pauvert A, et al. Rift Valley fever outbreak, Mayotte, France, 2018-2019. Emerg Infect Dis. 2020; 26(4): 769-72.

560. Zeller HG, Fontenille D, Traore-Lamizana M, Thiongane Y, Digoutte JP. Enzootic activity of Rift Valley fever virus in Senegal. Am J Trop Med Hyg. 1997; 56(3): 265-72.
